# Pre-assembled Nuclear Pores Insert into the Nuclear Envelope during Early Development

**DOI:** 10.1016/j.cell.2016.06.015

**Published:** 2016-07-28

**Authors:** Bernhard Hampoelz, Marie-Therese Mackmull, Pedro Machado, Paolo Ronchi, Khanh Huy Bui, Nicole Schieber, Rachel Santarella-Mellwig, Aleksandar Necakov, Amparo Andrés-Pons, Jean Marc Philippe, Thomas Lecuit, Yannick Schwab, Martin Beck

**Affiliations:** 1 European Molecular Biology Laboratory, Structural and Computational Biology Unit, 69117 Heidelberg, Germany; 2 European Molecular Biology Laboratory, Electron Microscopy Core Facility, 69117 Heidelberg, Germany; 3 Aix-Marseille Université, CNRS, IBDM UMR 7288, 13009 Marseille, France; 4 European Molecular Biology Laboratory, Cell Biology and Biophysics Unit, 69117 Heidelberg, Germany

## Abstract

Nuclear pore complexes (NPCs) span the nuclear envelope (NE) and mediate nucleocytoplasmic transport. In metazoan oocytes and early embryos, NPCs reside not only within the NE, but also at some endoplasmic reticulum (ER) membrane sheets, termed annulate lamellae (AL). Although a role for AL as NPC storage pools has been discussed, it remains controversial whether and how they contribute to the NPC density at the NE. Here, we show that AL insert into the NE as the ER feeds rapid nuclear expansion in *Drosophila* blastoderm embryos. We demonstrate that NPCs within AL resemble pore scaffolds that mature only upon insertion into the NE. We delineate a topological model in which NE openings are critical for AL uptake that nevertheless occurs without compromising the permeability barrier of the NE. We finally show that this unanticipated mode of pore insertion is developmentally regulated and operates prior to gastrulation.

## Highlights

• Annulate lamellae (AL) NPCs insert into the nuclear envelope during interphase
• AL-NPCs are pore scaffolds devoid of most transport channel nucleoporins
• NE-openings enable AL insertion, yet the permeability barrier remains unperturbed
• AL-NPC insertion operates only before gastrulation

## Graphical Abstract

**Figure:**
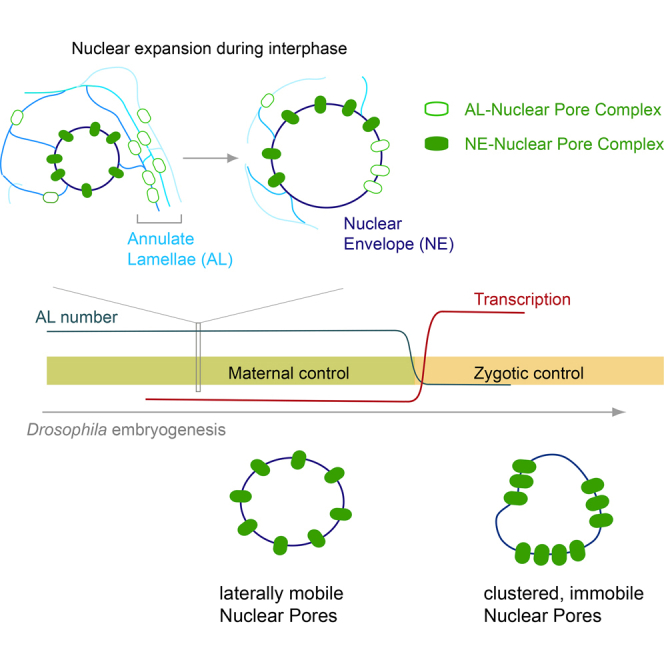

## Teaser

Rapidly growing embryos meet the challenge of stocking the nuclear envelope with nuclear pore complexes by keeping a ready store of pores in stacked membranous rings that feed into the envelope without puncturing it.

## Introduction

**Figure S1:**
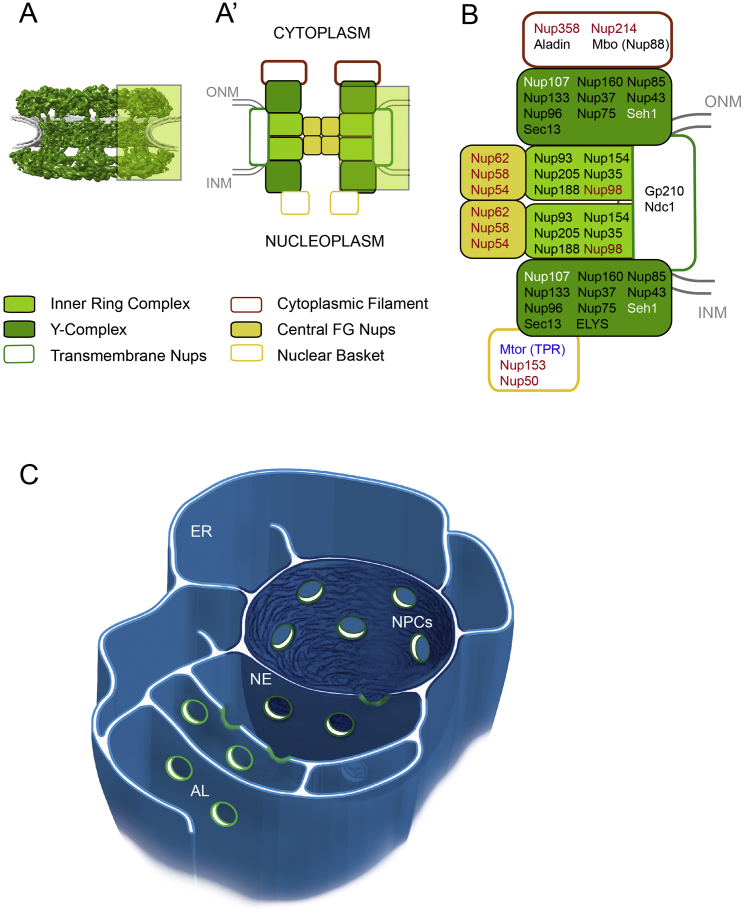
NPC Composition, Related to Figure 1 (A) Cryo-EM structure of the human NPC (von Appen et al., 2015) together with nuclear membranes (gray). (A′) Schematic representation of NPC sub-complexes. Transmembrane Nups, inner ring- and Y-complex are symmetric while distinct sub-complexes create asymmetry across the nuclear envelope. (B) Constituents of the *Drosophila* NPC. Nups that were used in imaging experiments in this study are highlighted in white (Y-complex Nups), red (FG-Nups) and blue (Mtor). (C) Nuclear envelope (NE), endoplasmic reticulum (ER) and annulate lamellae (AL) constitute one membrane continuum and share a common lumen.

In eukaryotes, the double membranous nuclear envelope (NE) encloses the nucleoplasm and separates it from the cytoplasm. The inner nuclear membrane (INM) provides contact with chromatin and the outer nuclear membrane (ONM) is continuous with the endoplasmic reticulum (ER). The two bilayers are fused at nuclear pore complexes (NPCs) that form aqueous channels through which regulated transport of macromolecules occurs. NPCs consist of multiple copies of ∼30 different nucleoporins (Nups) that are organized into biochemically distinct sub-complexes (Figures S1A, S1A′, and S1B). Two such modules, the inner ring complex (also called Nup93 complex) and the Y-complex (also called Nup107 complex) constitute the NPC scaffold that is symmetric across the NE plane. FG-Nups (containing phenylalanine-glycine rich intrinsically disordered protein domains) dock onto the scaffold. They constitute the permeability barrier and interact with translocating cargo complexes. Some of them (e.g., Nup214/88, Nup358 [RanBP2], and Nup153) introduce asymmetry by specifically binding to the cytoplasmic or nuclear face of the NPC, respectively (reviewed in Grossman et al., 2012) (Figure S1B).

Obviously, the sheer size and compositional complexity of NPCs renders its assembly and membrane insertion a very intricate task. Two distinct NPC assembly pathways that are temporally separated during the cell cycle have been described. First, during interphase, NPCs are assembled de novo onto an enclosed NE (D’Angelo et al., 2006). Interphase assembly occurs ubiquitously throughout eukaryotes and strictly requires the fusion of the INM and ONM by a mechanism that is only partially understood (Doucet and Hetzer, 2010). Second, no membrane fusion is required for NPC assembly at mitotic exit. This so-called postmitotic assembly mode is restricted to eukaryotes that disassemble their NPCs during mitosis into soluble sub-complexes after phosphorylation by mitotic kinases (Laurell et al., 2011). In anaphase, de-phosphorylation of Nups is thought to trigger the ordered re-assembly onto the separated chromatids before or while membranes enclose daughter nuclei (Doucet et al., 2010, Dultz and Ellenberg, 2010, Dultz et al., 2008). Both insertion mechanisms rely on the stepwise recruitment of pre-assembled sub-complexes. An insertion of pre-assembled NPCs into the NE has (to the best of our knowledge) not yet been described.

NPCs not only reside within the NE but are also found in stacked cytoplasmic membranes termed annulate lamellae (AL) that are a subdomain of the ER (Figure S1C) (Cordes et al., 1996, Daigle et al., 2001). Based on two-dimensional (2D) transmission electron micrographs these membrane stacks have been perceived as parallel membrane sheets decorated with NPCs (hereafter called AL-NPCs) that morphologically appear similar to their counterparts on the nuclear envelope (NE-NPCs) (Kessel, 1983). AL appear in some but not all transformed cell lines (Cordes et al., 1996, Daigle et al., 2001) and are highly abundant in germ cells and early embryos throughout animal phyla, including *Xenopus*, *Caenorhabditis elegans*, sea urchin, *Drosophila*, and also humans (Soupart and Strong, 1974). A role of AL as a storage compartment for maternally deposited Nups that can be made available for meiosis and the rapid cell cycles during early embryogenesis has been suggested (Lénárt and Ellenberg, 2003, Longo and Anderson, 1968, Spindler and Hemleben, 1982) but not experimentally proven. Despite these fundamental and long-standing pretensions the function of AL remains elusive and controversial, primarily for two reasons: (1) it has been difficult to conceive how the insertion of parallel stacked membrane sheets containing pre-assembled and possibly pre-oriented NPCs is topologically possible; and (2) direct experimental evidence for a contribution of AL-NPCs to the pool of NE-NPCs has never been obtained. On the contrary a previous study in *Drosophila* embryos has detected large soluble pools of transport channel Nups and concluded that NPC insertion likely proceeds from soluble cytosolic Nups (Onischenko et al., 2004).

**Figure S2:**
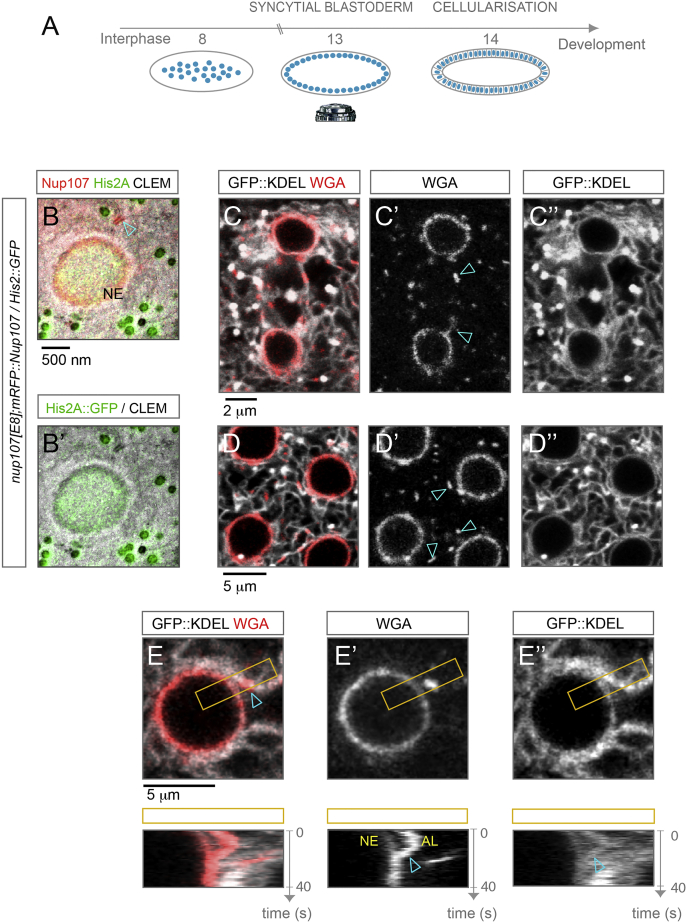
AL-NPCs Insert into the Nuclear Envelope, Related to Figure 1 (A) Schematic representation of early *Drosophila* embryogenesis. The somatic nuclei undergo 13 mitotic cycles in a common cytoplasm before formation of the first epithelial cell layer (cellularization) in the prolonged 14th interphase. Most of the presented experiments are performed in the syncytial blastoderm stage, where nuclei can be easily imaged by confocal microscopy due to their proximity to the microscope’s objective adjacent to the surface of the embryo. (B) Correlative light and electron microscopy (CLEM) of an embryo of the indicated genotype (see also Figures 1B–1B″). RFP::Nup107 fluorescence is concentrated along the NE and at AL-NPCs (arrowhead in B). Light microscopy images and electron micrographs of the same section are correlated by His2A::GFP in the nucleus and the auto-fluorescence of mitochondria in the GFP-channel (B′). (C–E) AL-NPC’s localize and insert along ER membranes. (C and D) Top view stills from a time lapse movie recording blastoderm embryos expressing the ER resident fusion protein GFP::KDEL (C″and D″), injected with WGA-Alexa555 labeling FG Nups (C′ and D′) in telophase (C–C″) or interphase (D–D″). AL-NPC reform on membranes at the spindle region in telophase (arrowheads in C′) and co-localize to ER membranes during interphase (arrowheads in D′). See also Movie S1. (E) AL-NPCs insert to the NE along ER membranes. Top view still and kymograph covering the boxed region of interest (ROI) in (E–E″) from a movie of a KDEL::GFP expressing embryo injected with fluorescently labeled WGA. WGA labeled AL-NPCs insert along ER membrane into the NE (arrowheads). See also Movie S3.

Here, we address the function of AL in the physiological context of the *Drosophila* blastoderm embryo that is rich in AL, while it undergoes a series of 13 synchronized mitoses in a syncytium (Figure S2A). Subsequently, the plasma membranes enclose the cortically aligned somatic nuclei in the extended 14^th^ interphase, forming the first epithelial cell layer before the embryo initiates gastrulation (Schejter and Wieschaus, 1993). This occurs concomitantly with the broad onset of transcriptional activity on the zygotic genome, a major developmental transition present in all metazoan (Newport and Kirschner, 1982). In the syncytial blastoderm, cell-cycle progression is very rapid, with interphase durations of ∼10 min during the early cell cycles. At least in mammalian cells, de novo NPC interphase assembly has been described to proceed with markedly slower kinetics (Dultz and Ellenberg, 2010). This led us to hypothesize that NPC assembly into a closed NE in *Drosophila* embryos might occur by a different, faster mechanism. By tracking NPCs in living embryos, we demonstrate direct uptake of AL-NPCs into the NE, as the ER feeds nuclear expansion. We derive a topological model that explains how the INM becomes continuous with inserting membrane sheets from the ER. We conclude that AL insertion to the NE is a previously unanticipated mode of NPC insertion that relies on pre-assembled, yet immature NPC scaffolds and operates prior to gastrulation.

## Results

### Nuclear Pores Insert from the ER into the NE

**Figure 1:**
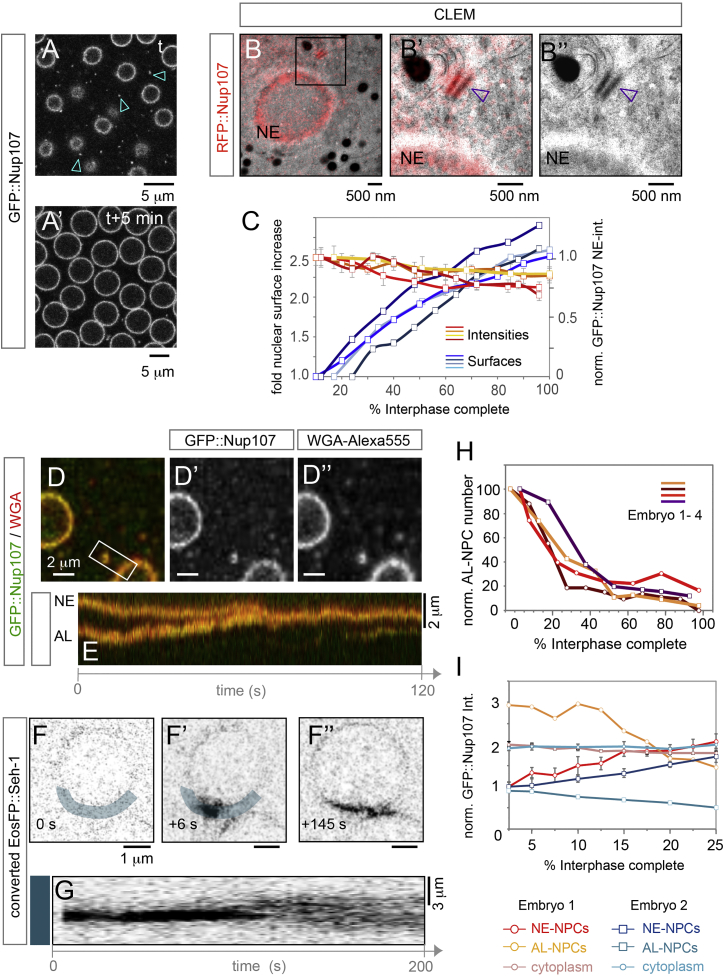
AL-NPCs Insert into the Nuclear Envelope (A and A′) Nuclear growth and NPC distribution during interphase in the *Drosophila* syncytium: Stills from a time-lapse movie recorded from a GFP::Nup107 expressing embryo imaged right after interphase onset (A) and 5 min later (A′). GFP::Nup107 localizes to the NE and to cytoplasmic foci (arrowheads) in (A), which disappear at t + 5 min (A′). (B–B″) Cytoplasmic foci of Nup107 fluorescence localize to AL-NPCs. Correlative light and electron microscopy (CLEM) of a RFP::Nup107 expressing interphase embryo. RFP::Nup107 is concentrated along the NE and at foci (boxed in B), that correspond to NPCs along ER membranes (arrowheads in B′ and B″). (C) NPC density stays constant as nuclei grow. Quantification of normalized nuclear surface increase (blue curves) and the mean GFP::Nup107 fluorescence intensity ± SD at the NE (red curves) during interphases. Values on both graphs are normalized to the earliest measured time point for each movie (n = 71 nuclei in four embryos). (D–E) The Y-complex protein Nup107 and WGA-labeled transport Nups co-localize at the NE and at AL-NPCs that insert to the NE. Top view still (D–D″) and kymograph (E) from a time-lapse movie imaging a WGA-Alexa555 injected syncytial blastoderm embryo expressing GFP::Nup107 (see also Movie S2). Insertion is captured in the kymograph (E) that spans the region of interest (ROI) boxed in (D). (F–G) Stills (F–F″) and kymograph (G) of the blue shaded ROI from a time-lapse movie recording photo-converted EosFP::Seh-1 before (0 s, F) or after (6 s, F′, and 145 s, F″) photo-conversion of AL-NPCs adjacent to the NE. NPC transfer from AL to the NE is documented by the lateral dispersion of the converted signal, which starts after ∼100 s (G). (H) AL-NPC number drops during early interphases. Quantification of the relative AL-NPC number inferred from GFP::Nup107 fluorescence for four embryos live-imaged over interphases. AL-NPCs were counted from 1 μm distant z sections spanning nuclear height in a constant field of view comprising ∼10 nuclei for each embryo. (I) GFP::Nup107 fluorescence intensity shifts from AL to the NE in the first 25% of interphases, when AL number drops the most (H). Fluorescence intensities were integrated over consecutive confocal slices covering the entire nuclear height at the NE (NE-NPCs) and at AL (AL-NPCs). Cytoplasmic GFP::Nup107 was determined from the mean fluorescence intensity. Analysis was done on two embryos (n = 13 and 11 nuclei, respectively). See Figure 1A for representative image; error bars represent ± SD over multiple ROIs. All images in Figure 1 are acquired from embryos in cycles 10–13 of the syncytial blastoderm stage. See also Figure S1 and Movies S2 and S4.

To investigate whether AL-NPCs contribute to the pool of NE-NPCs, we conducted live-imaging experiments in *Drosophila* blastoderm embryos before formation of the first somatic cell layer (Figure S2A). During this stage of development, AL are abundant and thus could potentially serve as a reservoir for NE-NPCs. To track NPCs throughout early embryogenesis, we expressed functional GFP or RFP fusions of Nup107 (Katsani et al., 2008) to image scaffold Nups and injected sub-critical concentrations of the fluorescently labeled lectin wheat germ agglutinin (WGA) to label FG-Nups. In interphase, GFP::Nup107 localized to the NE and to prominent foci throughout the cytoplasm (Figure 1A), similar to structures that were previously characterized as AL (Cordes et al., 1996, Daigle et al., 2001, Onischenko et al., 2004, Onischenko et al., 2005). As expected these foci also stained positive for transport channel Nups (Figures 1D–1D″) and always localized to membranes (Figures S2C–S2E″). To further confirm that these foci are morphologically identical to AL, we performed correlative light and electron microscopy. RFP::Nup107 fluorescence was strongly enriched along the NE and at AL (Figures 1B–1B″, S2B, and S2B′). We conclude that fluorescence imaging in life embryos is well-suited to study the spatiotemporal dynamics of annulate lamellae.

Image quantification revealed a 2.5- to 3-fold increase in nuclear surface during interphases, indicating a considerable uptake of ER membranes within a few minutes (Figures 1A, 1A′, and 1C). Thus, the surface area of the nucleus just before division is more than twice as large as the combined surfaces of the two daughter nuclei after division. This finding implies an excess of nucleoporins with respect to the available nuclear surface after mitosis. Both NE-NPCs and AL-NPCs disassemble during mitosis (Cordes et al., 1996, Onischenko et al., 2005, Stafstrom and Staehelin, 1984b). This leaves a fenestrated NE that, unlike in vertebrates, stays throughout mitosis around the separating sister chromatids and the mitotic spindle, except at centrosomes (Movie S1). Simultaneous with NE-NPC re-assembly at the daughter nuclei in late mitosis, AL-NPCs appeared first at membranes at the spindle (Figures S2C–S2C″; Movie S1), consistent with the idea of excess nucleoporins at late mitosis/early interphase.

To address if AL-NPCs contribute to the NE-NPC pool during the following interphase, we first determined if NE-NPC density decreases during nuclear surface expansion. We quantified the mean fluorescence intensities of GFP::Nup107 at the NE and found that it stayed almost constant (Figure 1C). As a consequence, NPCs have to insert constantly into the NE as its surface increases. Conversely, AL-NPCs were highly abundant in early but not late interphase (Figures 1A and 1A′). We therefore investigated their fate during interphase progression by tracking AL-NPCs in living embryos and found that they insert into the NE (Figures 1D–1G and S2E–S2E″; Movies S2 and S3), along ER membranes (Figures S2E–S2E″; Movie S3). To directly confirm the transfer of Nups from the observed cytoplasmic foci to the NE, we imaged embryos expressing photo-convertible Seh1-EosFP. After photo-conversion of a fluorescent spot close to a nucleus, the signal remained locally constrained for ∼100 s before it laterally resolved into the proximal NE over roughly the same time frame (Figures 1F–1F″ and 1G; Movie S4), suggesting a critical event prior to lateral diffusion. We conclude that AL-NPCs insert into the NE during blastoderm interphases.

Previous EM-based morphometry suggested that the total number of AL-NPCs stays constant in the syncytial blastoderm, i.e., during the first 90 min of *Drosophila* embryogenesis (Onischenko et al., 2004). However, if AL-NPCs considerably contribute to the pool of NE-NPCs by inserting into the NE while it expands, their number should decrease at least temporarily on much shorter timescales, namely during the ∼10 min of each interphase. Indeed, we found that AL-NPCs diminished as interphases progressed (Figure 1H). This reduction was particularly strong in the first half of interphase, when the rate of nuclear growth and thus NPC insertion was highest (Figure 1C). To estimate if the reduction of AL-NPC number reflects NPC redistribution from AL to the NE, we measured the respective GFP::Nup107 fluorescence levels at both compartments in three dimensions over time. For all quantified nuclei, the integrated NE fluorescence intensities of GFP::Nup107 increased between 70% and 100% within the first quarter of interphase, while inversely intensities at AL strongly decreased, mirroring AL disappearance (Figure 1I). This supports a scenario in which the pool of NE-NPCs is predominantly fed by integration of AL-NPCs. Alternatively, AL could disassemble and soluble Nups could add to pore formation at the growing NE from the cytoplasm. Yet this is unlikely since the GFP::Nup107 background intensity in the cytoplasm remained constant as AL disassembled (Figure 1I). We conclude that our data rather support a scenario in which pre-assembled NPCs insert from AL into the NE.

There are, however, major impediments that challenge the notion that intact NPC can insert into the NE: first, NPCs have an inherent compositional directionality across the NE plane (Figure S1B). If AL-NPCs were identical to NE-NPCs, they would be assembled asymmetrically in the absence of a nuclear compartment providing a directionality cue. They also had to be inserted into the NE in the correct orientation. Second, the integration of NPC-containing ER sheets into an intact NE poses striking topological obstacles. In particular, how an AL membrane sheet can become continuous with the INM of a sealed, intact NE is far from obvious. How is AL-NPC insertion thus possible?

### AL-NPCs Resemble Pore Scaffolds

**Figure 2:**
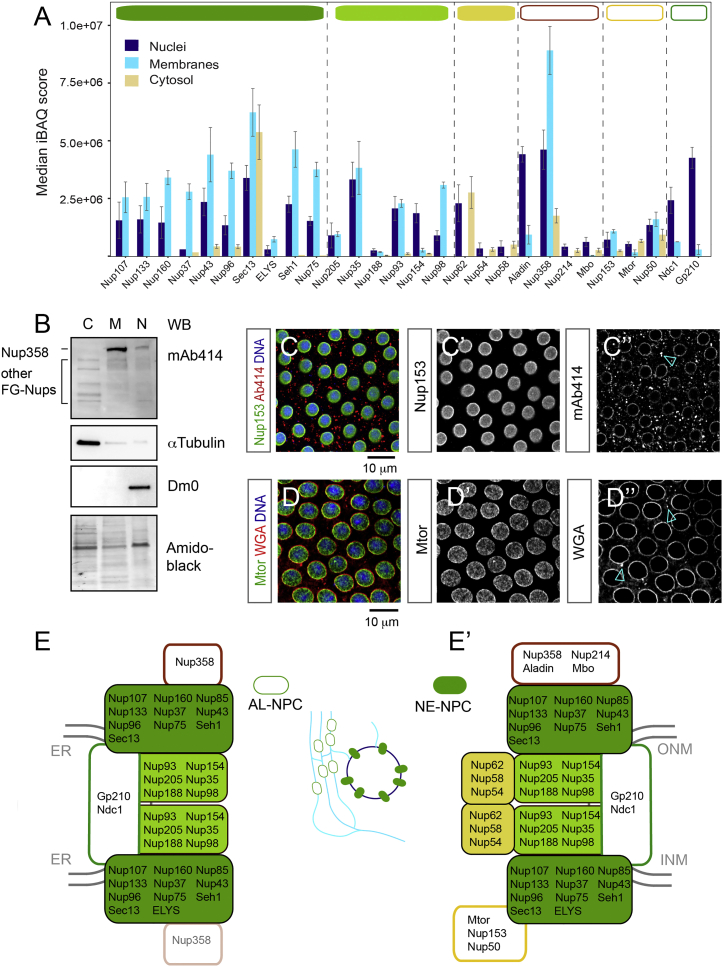
AL-NPCs Resemble Pore Scaffolds (A) Composition of NE-NPCs and AL-NPCs. Median intensity-based absolute quantification (iBAQ) scores of Nups detected in nuclei containing NE-NPCs, microsomal membranes containing AL-NPCs and cytosol after fractionation of *Drosophila* embryos in the syncytial blastoderm stage (n = 3 biological replicates). Nups are grouped into known subcomplexes and color-coded as represented in (E′). (B) Western blot analysis of fractionated *Drosophila* syncytial blastoderm embryos. The Lamin Dm0 is exclusively nuclear (N), while α-tubulin is strongly enriched in the cytoplasm (C), confirming fractionation quality. Detection with mAb414 recognizing a panel of FG-Nups reveals Nup358 predominantly in membranes (M) and nuclei but absent from the cytosol. Other FG Nups are mostly soluble (see text). Amido black shows equal loading. (C–D″) Top views onto a fixed *Drosophila* syncytial blastoderm embryo in interphase. The nuclear basket components Nup153 (C and C′) and Mtor (D and D′) are absent from AL-NPCs (arrowheads in C″, D″), which stain positive for mAb414 (C and C″) or WGA (D and D″), both labeling FG-Nups (including Nup358). (E and E′) AL-NPCs (E) are pore scaffolds made of transmembrane Nups, the inner ring, and Y-complex nucleoporins, extended by Nup358, which might or might not be attached symmetrically across ER membranes. NE-NPCs (E′) recruit soluble Nups to construct the mature pore that is asymmetric across the NE. See also Figures S3 and S4.

**Figure S3:**
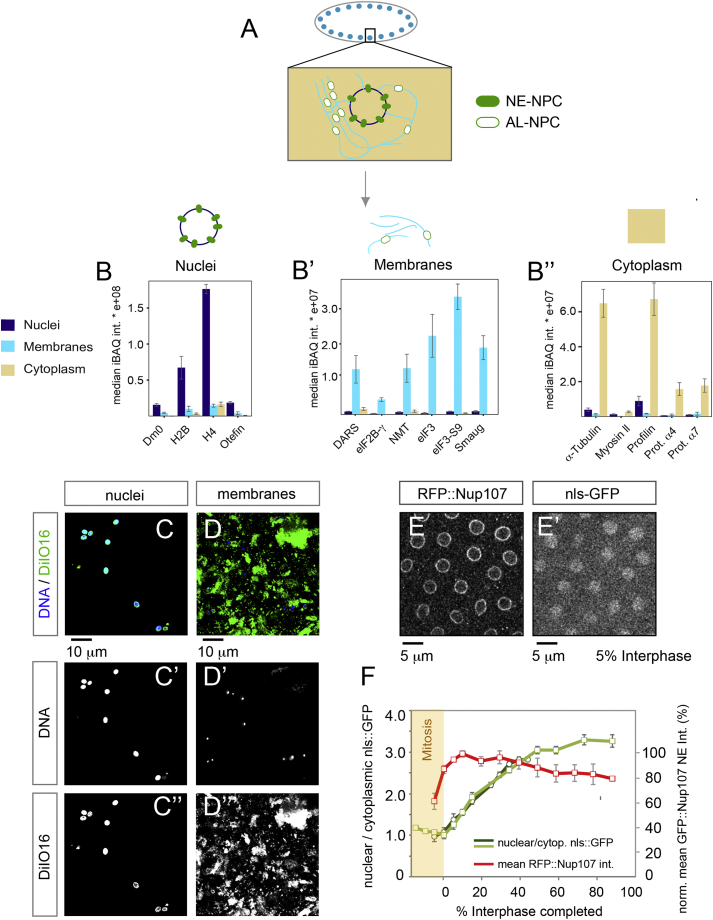
Sub-cellular Fractionation of Embryos, Related to Figure 2 (A) Biochemical fractionation of syncytial blastoderm *Drosophila* embryos. (B–D) Quality controls of fractionation. (B–B″) Median intensity based absolute quantification (iBAQ) scores of selected control proteins representative for either nuclei, membranes or cytoplasm, measured in the three respective fractions (n = 3 biological replicates). (C and D) Immunofluorescence of nuclear and membrane fractions, stained for DNA and with the membrane dye DiIO16, respectively. Nuclear integrity is preserved in the nuclear fraction (C and C′), where DNA is enclosed by DiIO16 labeled NE. No further membranes are attached to nuclei (C″). No nuclei are in the membrane fraction (D and D′). (E and F) Nuclear import of nls-GFP is delayed compared to NPC scaffold accumulation at the NE. Early interphase stills from a time lapse movie recording RFP::Nup107 (E) and nls-GFP (E′). (F) Mean fluorescence intensities of nuclear/cytoplasmic nls-GFP and of RFP::Nup107 at the NE were quantified in regions of interest (ROIs) on images as in (E and E′). Mean Intensities ± STDV (n = 23 nuclei in 2 embryos) are plotted as function of % interphase progression.

**Figure S4:**
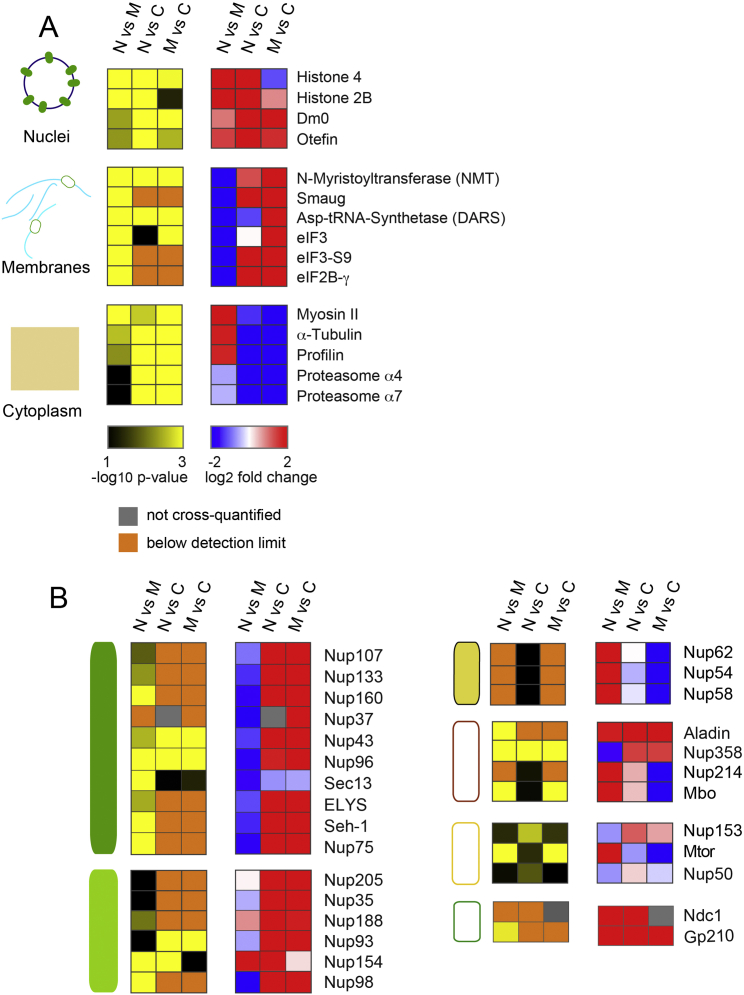
AL-NPCs Resemble Pore Scaffolds, Related to Figure 2 Alternative analysis and representation of the mass spectrometry data shown in Figures 2A and S3B–S3B″. Protein abundance fold-changes and respective p-values for control proteins (A) or Nups (B) are shown color-coded (n = 3 biological replicates). Orange color refers to cases in which the protein was consistently detected in three biological replicates of one fraction but was below the detection limit in all replicates of the other fraction (here, the fold change was set to 2). Nups are shown grouped into known subcomplexes; color-code according to Figure 2A.

The asymmetry of NPCs derives from sets of FG-Nups that are found exclusively either at the nucleoplasmic or cytoplasmic side of the NPC, in contrast to the symmetrically embedded scaffold Nups of the inner ring and Y-complexes (Figures S1A and S1B). We therefore explored if NPC composition was preserved in AL. We subjected blastoderm embryos to subcellular fractionation and comparatively analyzed fractions enriched for nuclei, microsomal membranes containing AL (devoid of the NE) and soluble cytosolic proteins by quantitative mass spectrometry (Figures S3A–S3D″, S4A, and S4B). We found that AL-NPCs contain the full set of NPC scaffold components, namely all the members of the inner ring and Y-complexes (Figures 2A and S4B). In contrast, their levels were low or undetectable in the cytosol, with the exception of Sec13, a known member of the cytosolic coatomer complex (Fath et al., 2007) (Figures 2A and S4B). These data further support the above-proposed scenario in which soluble pools of scaffold Nups cannot significantly contribute to the maintenance of NE-NPC number during interphase (see also Figure 1I).

The FG-Nups 358 and 98 displayed a subcellular distribution that was similar to scaffold Nups (Figures 2A and S4B). Both have been recently shown to critically contribute to NPC scaffold formation (Fischer et al., 2015, Stuwe et al., 2015, von Appen et al., 2015). Notably, the presence of Nup98 in AL-NPCs, a protein that is the essential constituent of the NPC permeability barrier (Hülsmann et al., 2012), suggests that NPCs are impermeable for larger molecules at all times. In contrast, the members of the Nup62/58/54 and Nup214/88 (the latter called Mbo in flies) complexes as well as the nuclear basket components Tpr (called Mtor in flies) and Nup153 were absent in AL-NPCs (Figures 2A–2D and S4B). Instead, the Nup214/88 and Nup62/58/54 complexes were highly abundant in the cytosol (Figures 2A, 2B, and S4B), in agreement with previous biochemical results that identified certain FG-Nups to be predominantly soluble and excluded from ER-membranes (Onischenko et al., 2004). One might thus surmise that NPC assembly is completed after insertion into the NE by recruiting soluble Nups from the cytosol in order to establish directionality and transport competence. Indeed, the nuclear accumulation of nls::GFP was delayed as compared to the burst phase of AL-NPC insertion (Figures S3E and S3F). We conclude that AL-NPCs are pore scaffolds devoid of most FG-Nups and all nuclear basket components. With the exception of Nup358, Nups that asymmetrically distribute across the NE-plane in NE-NPCs are absent from AL-NPCs (Figures 2E and 2E′).

### Topology of AL Insertion

**Figure 3:**
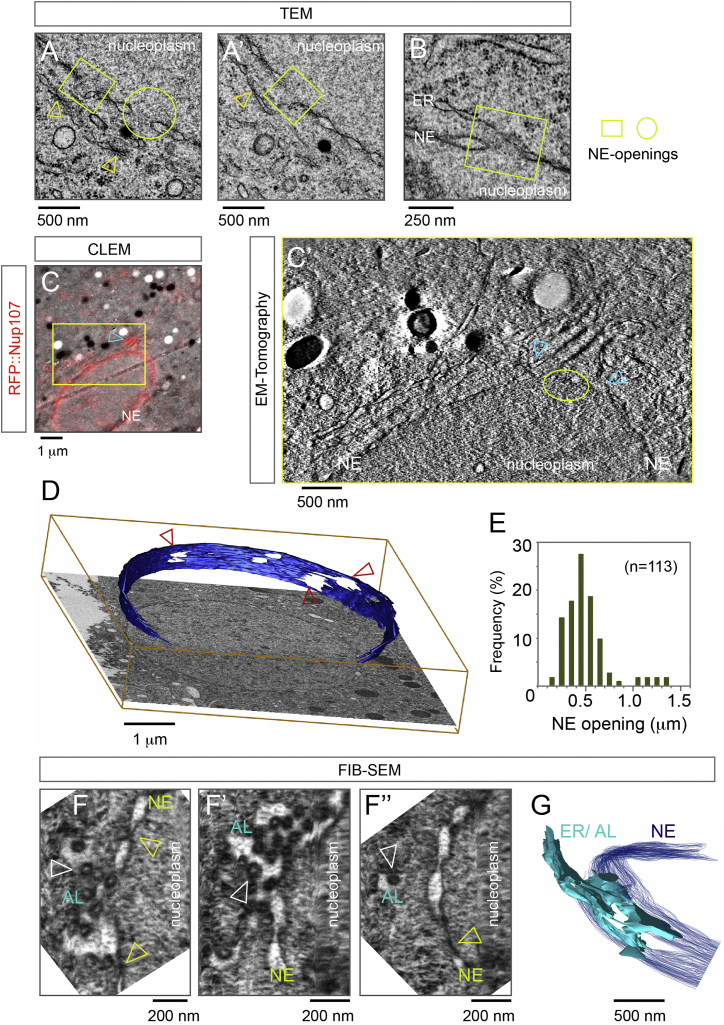
Topology of AL Insertion (A–B) NE-openings accompany AL insertions. (A and A′) Transmission electron micrographs (TEM) of two consecutive serial sections of a nucleus with closely aligned AL-NPCs (arrowheads). Note the clear INM-ONM connection of the encircled opening (A), which is replaced by intact NE in (A′), while the boxed opening is seen in both sections. (B) Electron micrograph of a nucleus where the NE is opened (boxed) and continuous with an ER stretch. (C) Capture of an AL-insertion into the NE by correlative light and electron microscopy (CLEM) and tomography (C′). (C) RFP::Nup107 localizes to the NE and is enriched at proximal AL-NPCs (arrowhead). (C′) Single slice through a tomogram recorded at the region depicted by the yellow box in (C). Arrowheads point to a membrane connection of the inserting AL to the NE, adjacent to a NE-opening. (D) Multiple NE openings (arrowheads) are apparent in a volume containing a nucleus that was obtained by focused ion beam-scanning electron microscopy (FIB-SEM; a single slice is shown superimposed with the NE that is isosurface-rendered in blue). (E) Histogram of NE-opening diameters measured in TEMs as represented in (A, A′, and B). Most NE-openings are in the range of 400–800 nm. (F–G) FIB-SEM visualizes the continuity of AL membrane sheets with the NE. Slices through a FIB-SEM volume tangential to the nucleus (F–F″) and isosurface rendering of the same region (G). (F–F″) Slices at slightly different angles. Note that cross-sectioned NE with NPCs in side view (yellow arrowheads in F–F″) are perpendicular to an AL membrane sheet that has a branched topology and contains multiple NPC in top view (white arrowheads in F–F″). See also Figures S5 and S6.

**Figure S5:**
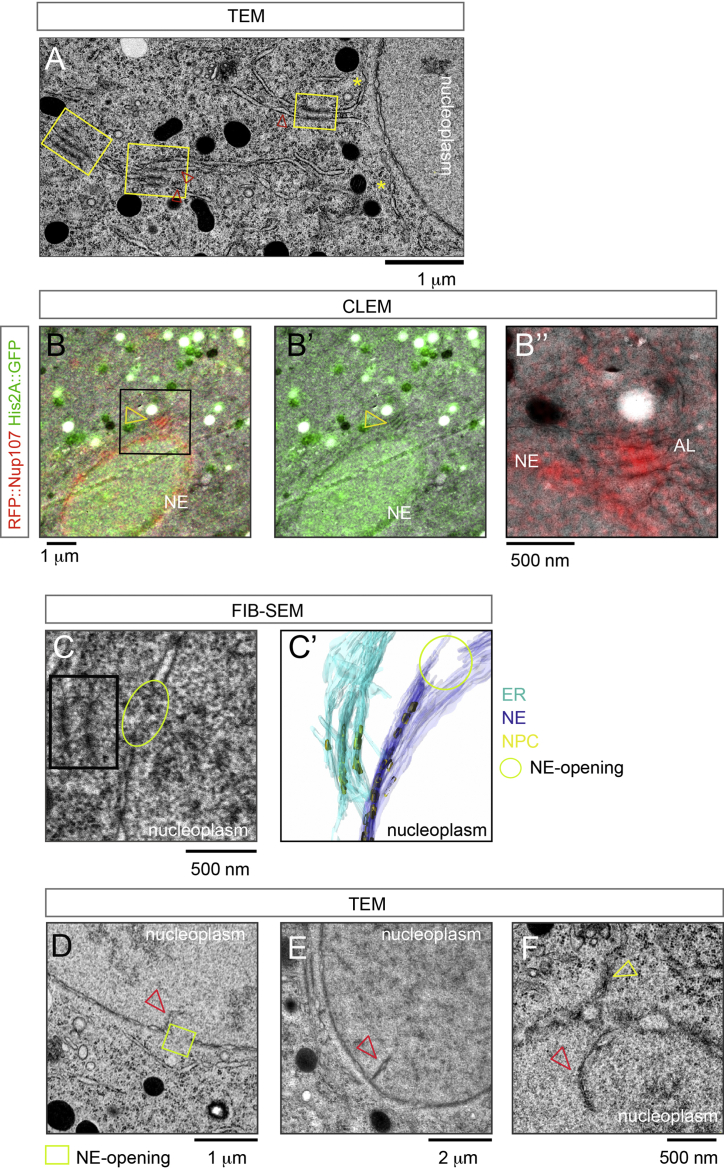
Topology of AL-Insertion, Related to Figure 3 (A) Transmission electron micrograph (TEM) of a *Drosophila* syncytial blastoderm embryo in interphase, showing the NE and adjacent AL (yellow boxes). AL appear as ribosome decorated (arrowheads) parallel ER stretches containing NPCs that morphologically resemble NPCs at the NE at the given resolution. (B–B″) Overview (B and B′) and higher magnification (B″) micrographs obtained by correlative light and electron microscopy (CLEM) showing AL-insertion into the NE (see also Figure 3C). RFP::Nup107 fluorescence is concentrated along the NE and at AL-NPCs (arrowhead in B and B′, AL in B″). Light microscopy images and electron micrographs on the same section are correlated by His2A::GFP in the nucleus and the auto-fluorescence of mitochondria in the GFP-channel (B′). (C–C′) Single slice (C) and isosurface-rendering (C′) of a volume obtained by Focused Ion Beam-Scanning Electron Microscopy (FIB-SEM), showing interconnectivity of stacked, AL membrane sheets (black box in C) that are oriented parallel to the NE (C and C′). AL-membranes connect to the NE adjacent to NE-openings. (D–F) TEM-sections depicting redundant NE membrane patches that are branched to the nuclear interior (red arrowheads in D–F). Note the redundant NPC containing membrane sheet close to an NE opening in (D). In (F) an inserting NPC containing ER stretch (yellow arrowhead) is close to redundant NE branching to the nucleoplasm (red arrowhead).

**Figure S6:**
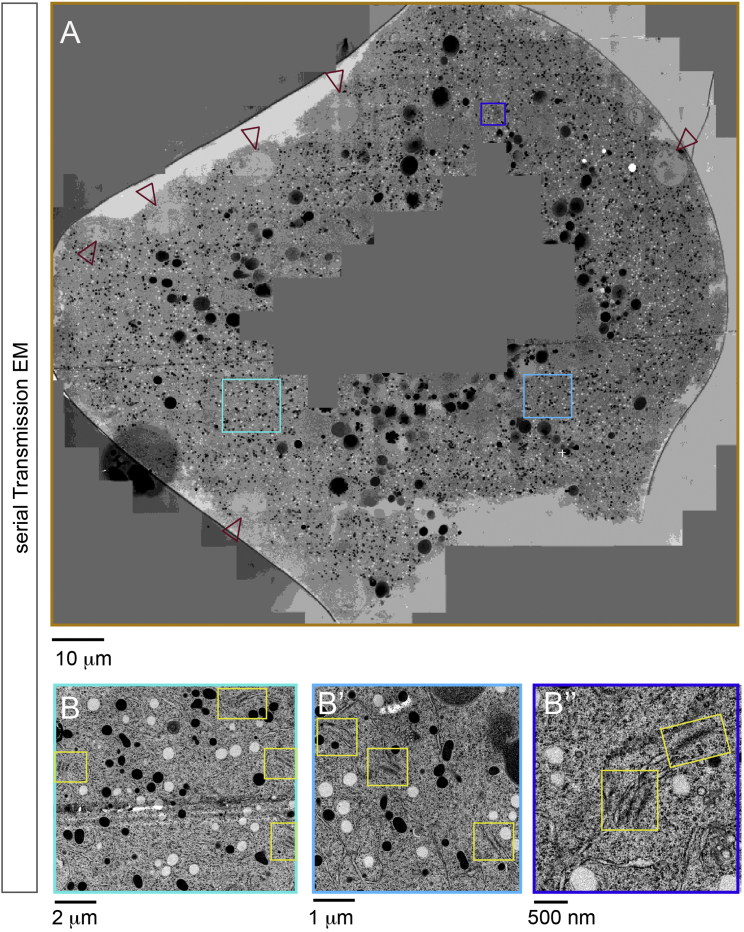
Annulate Lamellae Occur throughout the Embryo, Related to Figure 3 (A) Stitched Transmission electron micrographs (TEM) of an entire *Drosophila* syncytial blastoderm embryo in interphase, showing peripheral nuclei (arrowheads) and interior regions close to the yolk nuclei. AL occur close to cortical nuclei but also distant from dividing cortical nuclei (blue boxes). (B–B″) Higher magnifications of the blue-boxed areas in (A), showing AL with NPCs on parallel membrane sheets (yellow boxes).

To address how AL-NPC insertion is topologically possible, we sought to identify putative steps of AL insertion by ultrastructural analysis. We first analyzed sections through staged blastoderm embryos by transmission electron microscopy after high-pressure freezing and freeze substitution. AL were apparent in the cytoplasm throughout the embryo as interconnected stacks of membranes containing NPCs (Figures S5A, S6A, and S6B–S6B″). AL that were close to the NE and thus potentially could engage in an insertion event, often appeared continuous with the NE (Figures 3A, 3B, S5B″, and S5C′). Strikingly, as evident in multiple sections, these AL-NE fusion sites often were adjacent to apparent openings within the NE (Figures 3A and 3B). At the edges of these openings, the INM and ONM were seen to be fused in the electron micrographs, emphasizing that the gaps are not sample preparation artifacts (Figures 3A and 3B). We used correlative light and electron microscopy as described above and recorded an electron tomogram at a site where a fluorescent spot of RFP::Nup107 close to the NE indicated a potentially ongoing insertion event (Figures 3C, 3C′, and S5B–S5B″). Although the observed membrane topology in this region was complex, it clearly showed the critical features: a patch of AL engaged with the NE in direct proximity to NE openings. In conclusion, the unanticipated discovery of NE openings offers a topological explanation for how the INM becomes continuous with AL (see below).

A limitation of the aforementioned analysis is that it resolves membranes only when they are roughly aligned parallel to the electron optical axis and thus manifest as a projection in the electron micrographs. Because AL assume various orientations with respect to the NE it is relatively unlikely to capture both in a favorable two-dimensional projection. To better resolve inserting AL sheets in 3D, we used the slice and view technique, in which a low angle focused ion beam is used to mill away thin (5–10 nm) layers of an embedded specimen alternating with image acquisition by focused ion beam-scanning electron microscopy (FIB-SEM). The resulting volumes have an almost isotropic resolution and can be virtually rotated to obtain slices in basically any spatial direction. We first confirmed that the parallel AL-NPC decorated ER sheets are indeed highly interconnected in three dimensions and link to the NE close to openings of the nuclear membrane (Figure S5C″). NE-openings were frequent and surprisingly large (Figures 3D and 3E). By tangentially slicing the NE in volumes obtained by FIB-SEM, we could resolve inserting AL-sheets as part of an ER compartment that enclosed large parts of the respective NE-opening and contained NPCs (Figures 3F–3F″ and 3G). We conclude that AL insertion typically occurs in proximity to NE openings.

**Figure 4:**
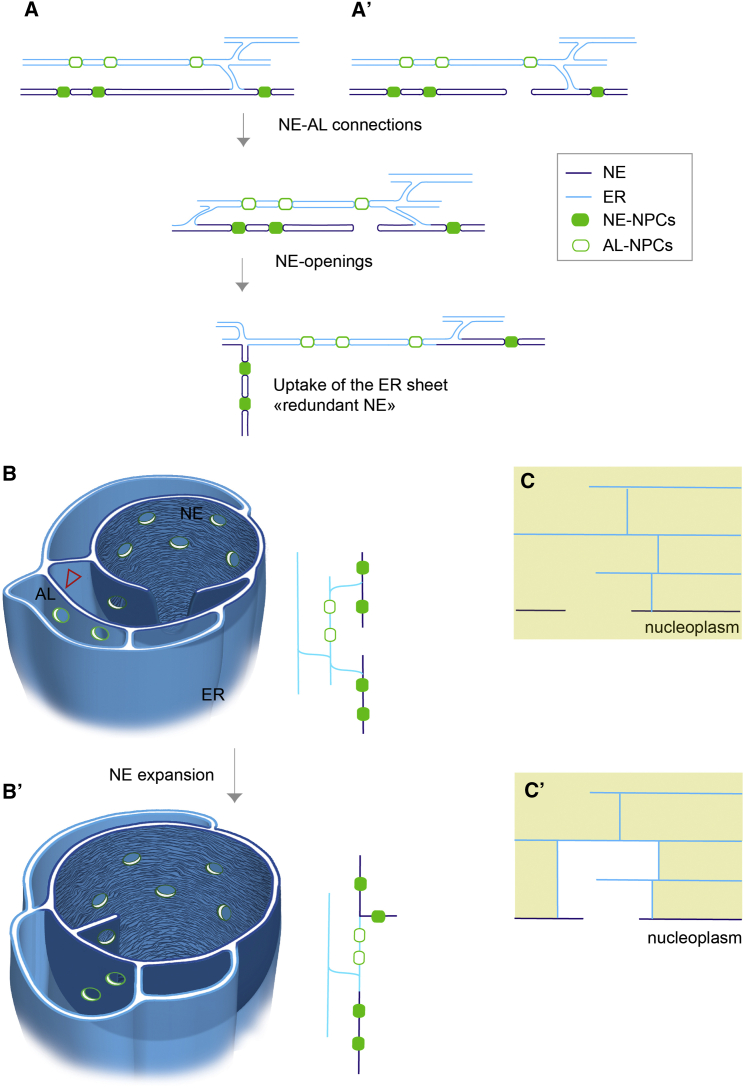
Model of AL-NPC Insertion (A–B′) Planar (A and A′) or three-dimensional (B and B′) model for AL-NPC insertion, as inferred from electron micrographs. Insertion involves the establishment of membrane connections from the inserting ER sheet containing AL-NPCs to the NE and opening of the NE adjacent to the connected ER sheet. NE openings could emerge de novo by an unknown mechanism (A) or alternatively could remain from the previous round of mitosis (A′). The connected ER sheet (arrowhead in B) becomes part of the NE while nuclear surface increases by lateral dissipation within linked membrane planes. Because pores limit lateral membrane dissipation, NE-NPCs located in between the NE opening and the AL-sheet connection predict the formation of transient redundant NE stretches (A and B′). (C and C′) The permeability barrier of the NE would be compromised if inserting ER sheets fail to entirely surround (or sufficiently “shield”) the NE opening against the cytoplasm (C). Alternatively no cytoplasmic influx through the NE opening would occur in a concealed compartment (C′).

The three-dimensional data allowed us to deduce putative topological intermediates of AL-insertion. Those involve establishing membrane connections from the adjacent sheet to the nuclear membranes and in proximity to openings of the existing NE. NE-openings could either form de novo or persist from the previous mitosis (Figures 4A and 4A′). Importantly their existence suggests a model for AL uptake that elegantly resolves the topological puzzle: NE openings link the INM to the inserting sheet and convert the latter into NE (Figures 3F, 3G, and 4A). Driven by nuclear expansion, both the adopted “novel” and the underlying “previously present” NE sheet laterally slide away from each other and augment nuclear surface (Figures 4A and 4B′). These topological intermediates can be viewed as part of a spatiotemporal continuum of AL and NE membranes. This model would predict that redundant pieces of NE should result from AL insertion (Figures 4A and 4B′). We indeed could confirm the existence of NPC-decorated, redundant NE membranes in both micrographs (Figures S5D–S5F) as well as nucleoplasmic GFP::Nup107 foci in live microscopy (Figure 6C). Such redundant NE could be resolved either by fission or re-insertion in an equivalent way as AL insertion from the cytoplasm.

### The Permeability Barrier of the NE Is Maintained during AL Insertion

**Figure 5:**
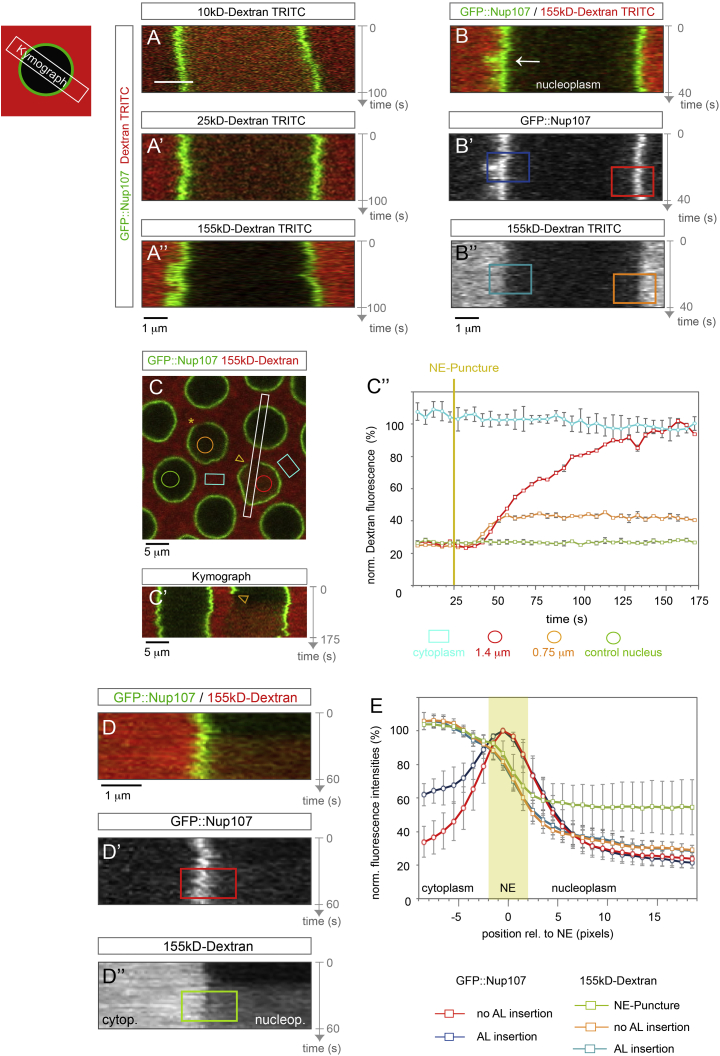
The Permeability Barrier of the NE Is Maintained during AL Insertion (A) Kymographs from time-lapse movies recorded in syncytial blastoderm embryos expressing GFP::Nup107 injected with fluorescently labeled dextrans of different molecular weight. Regions of interest (ROIs) for kymographs span entire nuclei as schematically depicted. The NE is permeable for 10 kDa (A) and 25 kDa dextran (A′), but not for 155 kDa dextran (A″). (B–B″) The NE stays impermeable upon AL-insertion. Kymographs (scheme) of a time-lapse movie imaging an embryo expressing GFP::Nup107 (B and B′) injected with dextran-155 kDa-TRITC (B and B″). Dextran stays cytoplasmic upon insertion of GFP::Nup107-labeled AL-NPCs (arrow). Colored ROIs refer to the graph in (E). (C–E) Laser puncture of the NE compromises its permeability barrier. (C) Top view still of a time-lapse movie imaging a GFP::Nup107 expressing syncytial embryo injected with dextran-155 kDa-TRITC, where the NE of two nuclei was laser punctured simultaneously (indicated by the arrowhead and asterisk, respectively). (C′) Kymograph of the respective movie along the ROI (white box in C). Dextran leaks into the nucleoplasm of the punctured nucleus (arrowheads in C and C′), but not in the neighboring control nucleus. (C″) Quantification of the mean dextran-TRITC fluorescence intensities ± SD for the respective ROIs color-coded in (C). Dextran accumulates in the nucleoplasm of nuclei within ∼20 s after puncture with an initial kinetics that is independent of the size of the punctured region. (D) Representative kymograph for GFP::Nup107 (D′) and dextran-155 kDa-TRITC (D″) after NE-puncture; colored ROIs refer to the graph in (E). (E) Dextran leaks into the nucleoplasm upon NE puncture, but not when AL insert to the NE. Quantitation of dextran influx upon NE-puncture and AL insertion. GFP::Nup107 and 155 kDa-dextran-TRITC levels were inferred from their respective fluorescence intensities, determined from line scans on ROIs in kymographs as exemplified in (B′), (B″), (D′), and (D″). Mean fluorescence intensities ± SD are plotted as a function of the distance from the NE for control nuclei (no insertion, n = 10 nuclei), nuclei with AL-NPC insertions (n = 7) or punctured nuclei (n = 7). Experiments were aligned by the respective maximal GFP::Nup107 intensity value, delineating the position of the NE. See also Movies S5 and S6.

The apparent NE openings suggest a compromised permeability barrier between the nucleoplasm and the cytosol, except that the NE openings would reside in a compartment that is entirely surrounded by ER-membranes (Figures 4C and 4C′). The electron microscopy data (Figures 3C′ and 3G) highlight the topological complexity of AL insertion and indicate that the same event might span a considerable fraction of the nuclear surface area. As such, it is not ultimately possible to conclude from the three-dimensional data whether or not the NE remains topologically closed during AL insertion. We therefore set out to experimentally test if the NE permeability can be maintained despite AL fusion. We imaged GFP::Nup107-expressing blastoderm embryos that were injected with fluorescently labeled dextrans of different molecular weight (Lénárt and Ellenberg, 2006). Small dextrans of 10 and 25 kDa were not excluded by NPCs and diffused into the nucleoplasm during interphase (Figures 5A and 5A′). In contrast, nuclei were impermeable to 155 kDa dextran (Figure 5A″), suggesting an intact barrier. Importantly, 155 kDa dextran did not even leak into the nucleoplasm as AL inserted to the NE (Figures 5B–5B″ and 5E; Movie S5), demonstrating that insertion of AL does not interfere with the permeability barrier of the nuclear membranes.

To test if an NE opening of the observed size would in principle cause dextran influx, we artificially ruptured the nuclear membranes by performing laser nano-surgery on the NE. Using GFP::Nup107, we targeted the NE with a 950 nm Titan Sapphire Laser and punctured the nuclear membranes (Figures 5C–5C″; Movie S6). Successful puncture was reflected by a strong mechanical response of the entire nucleus apparent as NE folding and tumbling (Movie S6). Strikingly, 155 kDa-dextran accumulated in the nucleoplasm of punctured nuclei within tens of seconds (Figures 5C′ and 5C″; Movie S6) with kinetics that did not depend on the size of the punctured region (Figure 5C″). These experiments conclusively demonstrate that the permeability barrier of the NE was disrupted after laser-induced rupture while it was not impaired when AL inserted (Figure 5E), despite comparable dimensions of the respective NE openings apparent in electron micrographs (Figure 3E). These findings are in line with our topological model and suggest that the NE-openings are entirely surrounded by ER membrane sheets.

### NPC Organization and Insertion Mode Change during Development

**Figure 6:**
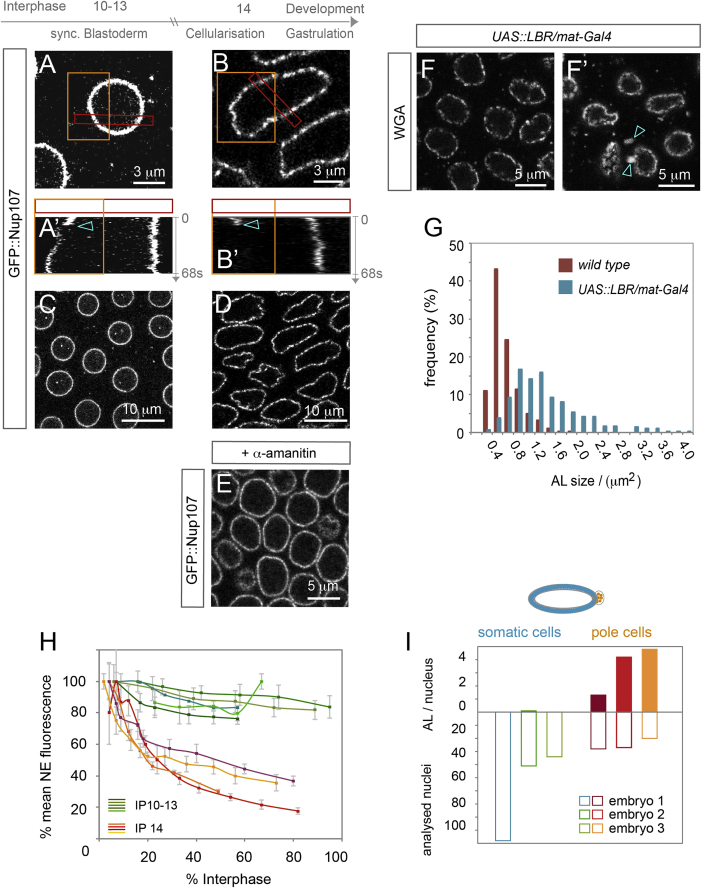
Developmental Regulation of NPC Organization and Insertion (A–D) Lateral mobility and organization of NPCs change during development. Representative top view stills (A–D) and kymographs (A′ and B′) of time-lapse movies imaging GFP::Nup107 in syncytial embryos (A, A′, and C) or before gastrulation onset in interphase 14 (B, B′, and D). GFP::Nup107 at the NE was photo-bleached (arrowheads in A′ and B′) at the depicted orange regions of interest (ROIs) (A and B) and recovered at syncytial blastoderm nuclei (A′) but not at nuclei from interphase 14 embryos (B′). For both kymographs the respective ROIs are boxed in red in (A) and (B). (C and D) GFP::Nup107 distributes evenly along the NE at the spherical nuclei of syncytial embryos (C) but appears clustered at the rim of the irregularly shaped nuclei at gastrulation onset (D). (E) Pore clustering is zygotically induced. Top view still from a movie recording a GFP::Nup107 expressing embryo in interphase 14, injected with α-amanitin, where nuclei stay round and pores fail to cluster due to inhibition of zygotic gene activation. (F–G) The zygotically induced gene LBR is sufficient to cluster NPCs and increase AL size. (F and F′) Top view stills from two syncytial blastoderm embryos where LBR expression was maternally induced. WGA labeled NPCs appeared clustered along the NE (F), similar to wild-type embryos in interphase 14 (B and D) and accumulate in larger AL-NPCs (F′) (arrowheads). (G) Histogram of AL-NPC sizes measured from images as shown in (C and F′). AL-NPCs are larger in blastoderm embryos where LBR expression was ectopically induced (n = 282 AL), compared to control embryos (n = 282). (H) The mode of NPC insertion is developmentally controlled. NPC density along the NE was inferred from the mean fluorescence intensities of GFP::Nup107 or fluorescently labeled WGA, which were normalized and plotted as a function of interphase progression. NPC densities stayed almost constant in syncytial interphases but strongly decayed in interphase 14 (see also Figure 1C). Plotted are mean fluorescence intensities ± SD for ∼10 nuclei from each imaged syncytial blastoderm (n = 5) or cellularizing (n = 4) embryo. (I) During interphase 14, AL-NPCs were diminished from the somatic nuclear layer at the embryo’s cortex but not from pole cells. See also Figure S7.

**Figure 7:**
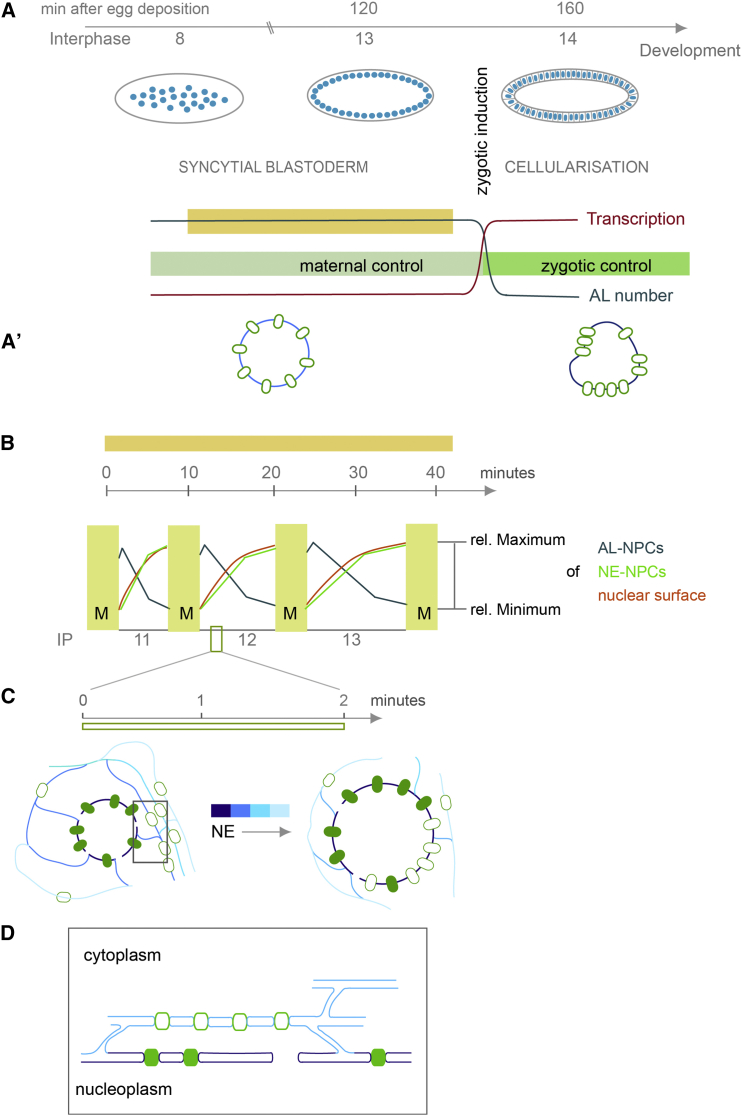
NPC Insertion in the Context of Embryonic Development (A) AL are abundant throughout the ∼120 min of syncytial development but diminish from the cortical layer in interphase 14, concomitant with cellularization and the transcriptional activation at zygotic induction. (A′) NE-NPCs are laterally mobile and all along the NE in the syncytial blastoderm, but immobilize and cluster at the NE starting with cellularization. (B) In each precedent interphase of the syncytial blastoderm, AL number oscillates within the cortical nuclear layer on a timescale of ∼10 min, with increasingly longer interphases in each cycle. Inverse to AL-NPC number, which decreases in each interphase, NE-NPC number increases together with nuclear surface expansion. (C and D) AL-NPCs insertion to the NE occurs in the range of 1–2 min. It involves an open NE and batch insertion of AL-NPCs within a proximal ER sheet (D). Lateral mobility allows NE-redistribution of inserted NPCs. Insertion of subsequent ER sheets augments nuclear surface (C).

In mammalian cell lines, NPCs are stationary embedded within the NE but mobile along ER membranes in AL (Daigle et al., 2001). Our results demonstrate frequent AL insertions in blastoderm embryos and indicate that AL-NPCs predominantly contribute to an increased NE-NPC number during nuclear expansion. Lateral mobility of NPCs within the NE could facilitate their re-distribution following AL insertion. We thus performed FRAP experiments on GFP::Nup107 expressing embryos to test whether AL-NPC became immobile upon NE insertion. Strikingly, we observed fast recovery of GFP::Nup107 all along the rim after photobleaching (Figures 6A and 6A′). Together with our finding that NPC material laterally dispersed after AL insertion to the NE (Figures 1F–1F″), we conclude that in the *Drosophila* syncytial blastoderm NPCs are mobile within the NE. In contrast, GFP::Nup107 fluorescence did not recover after photo-bleaching of nuclei at the onset of gastrulation, suggesting that dispersion of pores within the envelope was abolished (Figures 6B and 6B′). The impaired NE-NPC mobility in those nuclei was reflected by distinct principles of pore organization along the NE when compared to nuclei in younger embryos. GFP::Nup107 distributed uniformly along the NE of blastoderm embryos but appeared clustered into distinct steady foci just before embryos started to gastrulate (Figures 6C and 6D). The switch in NPC mobility and organization coincides with the activation of the zygotic genome (Figure 7A). Thus pore organization at the NE could be controlled by zygotic genes or by transcription-associated changes in chromatin. Consistent with both hypotheses, injection of the RNA polymerase inhibitor α-amanitin prevented clustering of NPCs on later stage nuclei (Figure 6E).

**Figure S7:**
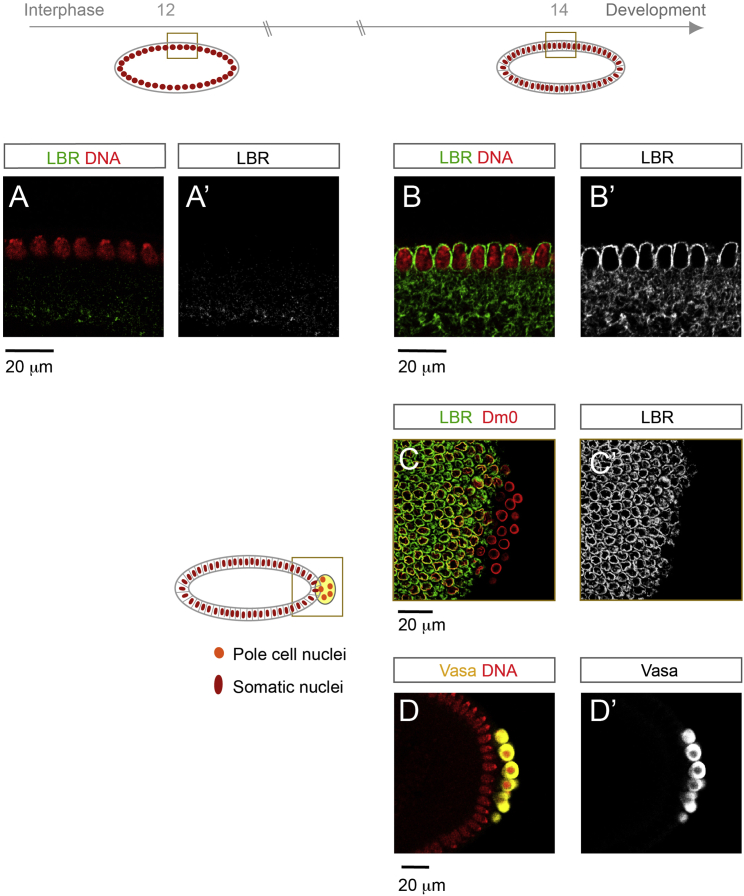
NE Targeting of LBR Is Developmentally Regulated, Related to Figure 6 Sagittal views (A, A′, B, B′, D, and D′) or optical cross section (C and C′) of fixed *Drosophila* embryos in the syncytial blastoderm stage (A and A′) or during cellularization in interphase 14 (B–D′). LBR does localize to the NE in the elongated prospective somatic nuclei during interphase 14 (B and B′) but not before (A and A′). (C and D) LBR targeting to the NE is cell type specific. Optical cross section of a fixed interphase 14 embryo stained with antibodies against the *Drosophila* Lamin Dm0 and LBR. Dm0 localizes to the periphery of somatic nuclei and the posteriorly located nuclei of pole cells, the germ cell progenitors. LBR is excluded from pole cell nuclei. Posterior pole cells stain positive for the Vasa protein, a specific pole cell marker (D and D′). Posterior is to the right in (C and D).

The nuclear lamina, a meshwork of intermediate filament proteins that underlies the NE and projects into the nucleoplasm in metazoa is critical for NPC organization and mobility within the NE (Daigle et al., 2001), but other NE proteins could also be important for NPC mobility. Comparative analysis of the proteomes of isolated nuclei from either blastoderm or gastrulating embryos revealed a significant (p = 0.00024) ∼2.5-fold enrichment of a very prominent INM protein, lamin B receptor (LBR), in older nuclei (data not shown). In mammalian cells LBR is recruited by the Y-complex member ELYS/Mel28 to specific NE-subdomains and could thus directly link to NPC distribution (Clever et al., 2012). Strikingly, LBR was absent from the NE in syncytial embryos but became localized to the rim of somatic nuclei during cellularization in interphase 14 (Figures S7A and S7B). Notably, the protein remained undetectable at the NE of nuclei from pole cells, which are the posteriorly localized germ cell progenitors (Figures S7C and S7D). To address whether LBR is sufficient to alter NPC organization at the NE, we ectopically expressed the protein in WGA-injected syncytial blastoderm embryos. In these embryos pores appeared clustered within the NE and nuclei acquired an irregular morphology (Figure 6F). Both phenotypes are reminiscent of wild-type nuclei at gastrulation onset. Strikingly, ectopic expression of LBR in the early embryo also induced larger AL sizes (Figures 6F′and 6G), implying that LBR expression and NPC clustering counteracts AL-insertion.

At last, we detected striking differences in the NPC insertion mode between the different developmental stages. In contrast to the early embryo (compare to Figure 1C), the mean fluorescent intensities of either GFP::Nup107 or fluorescently labeled WGA at the nuclear rim strongly decreased as interphase 14 proceeded (Figure 6H), while nuclei significantly increase their surface area (Fullilove and Jacobson, 1971). Interestingly, the switch in NPC organization and insertion is concomitant with the reported (Onischenko et al., 2004, Stafstrom and Staehelin, 1984a) and observed (Figures 6D and 6I) disappearance of AL-NPCs from the cortical nuclei layer at early gastrulation. At the same time AL remained abundant in pole cells (Figure 6I), suggesting that nuclei from the prospective soma and germline have different NE organizations, compatible with our results on differential LBR localization (Figure S7). Jointly, our data suggest that AL insertion to the NE is an “early developmental” program that is reduced or lost as the embryo matures (Figure 7).

## Discussion

Collectively, the following scenario emerges from our data. AL are abundant in early *Drosophila* embryos and predominantly contribute to maintain the constant NE-NPC density in the expanding NE during interphase. The abundance of AL at the cortical nuclei layer thereby oscillates together with the progression of the consecutive interphases until the start of global transcription when AL disappear and the mode of NPC insertion changes (Figures 7A and 7B). During each onset of early interphases, AL-NPCs are assembled similarly to NE-NPCs but since the combined nuclear surface of the two daughter nuclei is smaller as compared to the parental nucleus, they remain in the cytoplasm. As interphases progress, AL-NPCs feed into the pool of NE-NPCs alongside ER membranes that augment NE surface during rapid nuclear expansion (Figure 7C). AL insertion is enabled by NE openings that might either persist from previous mitosis or form de novo by an unknown mechanism (Figure 7D). Upon AL insertion, the NE permeability barrier remains unperturbed, likely because the NE openings are entirely surrounded by the ER network. The inserting NPCs comprise pre-assembled NPC scaffolds that recruit the full set of Nups only subsequent to insertion and only then establish transport competence.

Why do the expanding nuclei of the syncytial blastoderm maintain a constant number of NPCs per surface area despite their transcriptional inactivity? One might surmise that this is due to mechanical properties but also temporal constraints. The insertion of NPCs might be crucial to enable the massive influx of material into the nucleoplasm during nuclear expansion (volume increase). Indeed, the strained configuration of nuclei is reflected by their strong mechanical response (NE tumbling) upon disruption of the NE and permeability barrier after laser puncture. Second, the batch transfer of entire NPC scaffolds as inherent parts of membrane sheets overcomes the described kinetic constrains of interphase assembly in mammalian cells, that are not compatible with the short interphases in the *Drosophila* syncytium (Dultz and Ellenberg, 2010). Given the abundance of AL-NPCs and the reported high insertion rate of NPCs into the NE of *Xenopus leavis* oocytes (D’Angelo et al., 2006) it appears likely that similar mechanisms operate in vertebrates. It remains unclear how sufficient amounts of AL are generated to globally feed nuclear surface expansion over multiple cell cycles until the start of transcription. However, Nups are maternally provided and AL are abundant not only at the cortical layer of nuclei but also within the interior of the embryo (Figures S6A and S6B–S6B″). Therefore, a possibility that needs to be considered is that a source of AL-NPCs already generated during oogenesis feeds nuclear growth throughout the syncytial blastoderm.

In addition to their eminent role in transport, NE-NPCs organize the nuclear periphery by delineating zones of active euchromatin as compared to transcriptionally repressed heterochromatin in between pores (Ptak et al., 2014). Crucial to this is that NPCs are laterally immobile within the NE, which was shown to depend on the nuclear lamina (Daigle et al., 2001). Lamins are nuclear intermediate filament proteins and come in two major types: B-type Lamins are ubiquitous, while A-type Lamins are expressed exclusively when cells differentiate. Both proteins engage in distinct meshworks and also impact on NPC insertion rate (Lenz-Böhme et al., 1997, Liu et al., 2000). Our work puts NPC organization and the mode of pore insertion into a developmental context. We propose that in *Drosophila* AL insertion is innate to earliest embryogenesis and diminishes when pores get laterally restricted and cluster at the NE. There are no A-type lamins expressed at that stage, and specifically expressed INM proteins could be crucial. Intriguingly, the formation of immobile pore clusters coincides with the transcriptional upregulation of hundreds of genes at zygotic induction, a developmental transition present in all metazoan that is accompanied by characteristic changes in chromatin signatures (Rudolph et al., 2007, Vastenhouw et al., 2010). We reveal that the zygotically upregulated INM protein LBR, a developmentally controlled INM tether of peripheral heterochromatin (Solovei et al., 2013), is sufficient to prematurely aggregate NPCs in blastoderm interphases, when artificially expressed earlier in embryogenesis. This also leads to larger AL likely because LBR counteracts AL insertion for which lateral NPC mobility is required. Our data suggest a zygotically induced regulation that links pore insertion and organization, NE composition and ultimately also chromatin organization at the nuclear periphery. All of these events eventually contribute to the commitment of originally pluripotent somatic nuclei into distinct lineages.

## Experimental Procedures

Detailed experimental procedures are available in the Supplemental Information.

### Embryo Injections, Live Imaging, and Immunostainings

Staged embryos were treated according to standard protocols and injected with Alexa488 or Alexa555 conjugated WGA (100 μg/ml, Life Technologies), α-amanitin (100 μg/ml, Sigma) or TRITC/FITC conjugated dextrans of different molecular weight (Lénárt and Ellenberg, 2006). Subsequently embryos were imaged on an inverted Zeiss LSM780 confocal microscope equipped with a 63×/1,4 NA oil immersion objective. For immunostainings, embryos were fixed in 4% formaldehyde and processed according to standard protocols.

### Sub-cellular Fractionation and Protein Identification by Mass Spectrometry

Dechorionated embryos were lysed and nuclei were isolated by centrifugation at 5,000 rpm for 13 min and stripped from attached membranes by centrifugation (45 min, 12,000 rpm) through a 1 M sucrose cushion. Microsomal membranes were isolated by spinning the supernatant from the nuclear precipitate at 40,000 rpm for 45 min. Samples were further processed and analyzed by shotgun mass spectrometry as previously described (Mackmull et al., 2015). Raw files for quantitative label free analysis were analyzed using MaxQuant (Cox and Mann, 2008) and the MS/MS spectra were searched against the *Drosophila* Swiss-Prot entries using the Andromeda search engine (Cox et al., 2011). Protein differential expression was evaluated using the Limma package. Differences in protein abundances were statistically determined using the Student’s t test moderated by the empirical Bayes method. Significant regulated proteins were defined by a cut-off of log2 fold change ≤−1 or ≥1 and p value ≤ 0.01.

### Transmission Electron Micrograph, FIB-SEM, and Correlative Light and Electron Microscopy Imaging

Embryos were high pressure frozen, freeze-substituted and infiltrated with resin. Blocks were subsequently trimmed for FIB-SEM or cut into 300-nm sections for correlative light and electron microscopy (CLEM) analysis using an ultramicrotome. For serial transmission electron microscopy (TEM), the resin-embedded embryos were trimmed and consecutive 100 nm distant sections were obtained with a section thickness of ∼80 nm. TEM imaging was carried out on a FEI Tecnai F30 equipped with Gatan US4000 CCD camera, operated at 300 kV or a FEI Biotwin equipped with an Olympus Keen View G2 camera operated at 120 kV, respectively. The fluorescence microscopy (FM) imaging was carried out as previously described (Avinoam et al., 2015, Kukulski et al., 2011). Tomography was performed in 1° increments at 4,700× magnification on a FEI Tecnai F30 electron microscope. FIB-SEM imaging was carried out on an Auriga 60 (Zeiss) using the Atlas3D software. Datasets were acquired with 5 nm pixel size and 5 nm steps in z and aligned using TrakEM (Fiji).

## Author Contributions

B.H. conceived the project, designed and performed experiments, analyzed data, and wrote the manuscript. M.T.M. designed and performed experiments and analyzed data. P.M., P.R., K.H.B., N.S., R.S.M., A.A.P., A.N., and J.M.P. performed experiments. T.L. designed experiments. Y.S. designed experiments and oversaw the project. M.B. conceived the project, designed experiments, analyzed data, oversaw the project, and wrote the manuscript.

## Accession Numbers

The accession number for the mass spectrometry proteomics data reported in this paper is ProteomeXchange Consortium: PXD004120.

## Supplemental Information

Document S1. Supplemental Experimental Procedures

Movie S1. AL Assemble along ER Membranes, Related to Figure 1 Syncytial blastoderm *Drosophila* embryo expressing KDEL::GFP, injected with Alexa555-WGA labeling FG-Nups, imaged from the top during mitosis. WGA becomes soluble as NPCs dis-assemble in prophase and re-concentrates around daughter nuclei and at AL-NPCs along ER membranes in telophase. One frame was imaged every 6 seconds.

Movie S2. AL-NPCs Insert to the NE during Interphase, Related to Figure 1 Top-view time lapse movie of a syncytial blastoderm *Drosophila* embryo expressing GFP::Nup107 injected with Alexa555-WGA. The Y-complex protein Nup107 and FG Nups co-localize at AL-NPCs as they insert to the NE (arrow). Stills and a kymograph of this movie are shown in Figures 1E and 1F. The movie was imaged at 3 frames / second.

Movie S3. AL-NPCs Insert along Membranes to the NE, Related to Figure 1 Top-view time lapse movie of a syncytial blastoderm *Drosophila* embryo expressing GFP::Nup107 injected with Alexa555-WGA. AL-NPCs insertion to the NE (arrow) occurs on ER membranes. The embryo was imaged at 1 frame / second.

Movie S4. Uptake of AL-NPCs to the NE, Related to Figure 1 Time lapse movie of a syncytial blastoderm *Drosophila* embryo expressing EosFP::Seh1 during interphase. Photo-conversion was performed at an AL-NPC spot adjacent to the NE, 6 seconds after start of the movie. Panels show the combined channels (left) and the photoconverted channel (right). Note that lateral dissipation of the converted EosFP::Seh1 starts only about 100 seconds after photo-conversion. Stills and a kymograph of this movie are shown in Figures 1G–1G″. The embryo was imaged at 1 frame every seconds.

Movie S5. The Permeability Barrier of the NE Is Maintained upon AL Insertion, Related to Figure 5 Top view time lapse movie of a syncytial blastoderm *Drosophila* embryo expressing GFP::Nup107 injected with TRITC conjugated Dextran-155kD during interphase. Dextran-155kD is excluded from nuclei, as GFP::Nup107 labeled AL-NPCs insert to the NE (arrow). The movie was acquired at 1 frame / second.

Movie S6. Laser Puncture of the NE Impairs Its Permeability Barrier, Related to Figure 5 Top view time lapse movie of a syncytial blastoderm *Drosophila* embryo expressing GFP::Nup107 injected with TRITC conjugated Dextran-155kD during interphase. Dextran is excluded from nuclei, but fills the nucleoplasm after laser-induced puncture of the NE of two nuclei 25 seconds after the movie starts. Targeted nuclei are indicated by an arrow and an arrowhead, respectively. Quantitative analysis of this movie is shown in Figures 5C–5C″. Note the strong mechanical response of nuclei after puncture, indicated by irregular NE jiggling. A frame was imaged every 5 seconds.

Document S2. Article plus Supplemental Information
