# Supplementary material for: Pre-assembled Nuclear Pores Insert into the Nuclear Envelope during Early Development
Source: Cell. 2016 Jul 28;166(3):664–78. doi: 10.1016/j.cell.2016.06.015 (PMC4967450; doi:10.1016/j.cell.2016.06.015)
Supplement: Document S1. Supplemental Experimental Procedures [file mmc1.pdf]

Cell, Volume *166*

Supplemental Information

Pre-assembled Nuclear Pores Insert

into the Nuclear Envelope

during Early Development

Bernhard Hampoelz, Marie-Therese Mackmull, Pedro Machado, Paolo Ronchi, Khanh Huy Bui, Nicole Schieber, Rachel Santarella-Mellwig, Aleksandar Necakov, Amparo Andrés-Pons, Jean Marc Philippe, Thomas Lecuit, Yannick Schwab, and Martin Beck

Supplemental Experimental Procedures

Fly strains and antibodies

The following fly lines were used in this study: $w^{*}wg^{Sp\text{-}1}$*/CyO; P{mGFP-Nup107.K}9.1 (Katsani et al, 2008)* (BL-35514); $w^{*}wg^{Sp\text{-}1}$*/CyO; P{mRFP-Nup107.K}7.1 (Katsani et al., 2008)* (BL-35517); w^{*}*; Nup107*^{E8}*/CyO; P{mRFP-Nup107.K}7.1, P{His2Av*$^{T:Avic\backslash GFP\text{-}S65T}$*}62A (Katsani et al., 2008),* (BL-35518); w^{118}*; P{*w^{+}*,UASp-Lys::GFP-KDEL}; P{*w^{+}*, nanosGal4:VP16}*; $y^{1}w^{67c23}$ *, P{*w^{+mC}*=Ubi-GFP.nls}ID2; P{Ubi-GFP.nls}ID3* (DGRC-106455). *yw* flies were used as wild type controls.

Embryo injections, Live Imaging and Immunostainings

For live-imaging analysis, staged syncytial blastoderm embryos of the respective genotype were treated as described (Cavey and Lecuit, 2008) and injected with Alexa488 or Alexa555 conjugated WGA (100 µg/ml, Life Technologies), α-amanitin (100 µg/ml, Sigma) or TRITC/FITC conjugated Dextrans of different molecular weight (Lenart and Ellenberg, 2006). Subsequently embryos were imaged on an inverted Zeiss LSM780 confocal microscope equipped with a 63x/1.4 NA oil immersion objective. Photo-bleaching was performed on GFP::Nup107 expressing embryos with a 488 nm Argon laser (100%, 5.2 mW photonic output) and 10 iterations. For laser-ablation of the NE, syncytial blastoderm embryos expressing GFP::Nup107 injected with 155kD-Dextran-TRITC were mounted and imaged on an inverted Zeiss LSM 780 NLO confocal microscope. Embryos were imaged using a 63x /1.3 NA oil objective and ROIs along the NE of individual nuclei were punctured by targeting a 950 nm Two Photon Laser at 100% laser power with one iteration. For photo-conversion

experiments, staged embryos expressing Eos-FP::Seh1 were processed in the dark and mounted on a Zeiss LSM510 inverted confocal microscope. Photo-conversion was done with a 405 nm laser with 5% laser power with 40 iterations. For Immunofluorescence experiments, staged embryos were dechorionated and fixed with 4 % Formaldehyde (FA) for 20 min, followed by Methanol de-vitellinization. Fixed embryos were proceeded for incubation with the following primary antibodies: mouse anti-Dm0 (1:500, DSHB), rat anti-Vasa (1:2000), rat anti-Mtor (1:500) (Mendjan et al., 2006), rabbit anti-Nup153 (1:500) (Mendjan et al., 2006), guinea pig anti-LBR (1:1000) (Wagner et al., 2004), mouse-anti Ab414 (1:500, Covance). Secondary antibodies were Alexa conjugates (1:500, Life Technologies), and DNA was stained with Hoechst dye (1:1000) Mounted stained embryos were imaged on Zeiss LSM780 or LSM510 confocal microscopes respectively. For Western Blot analysis antibodies were used in the following concentrations: mouse-anti Dm0 (1:100, DSHB), mouse-anti Ab414 (1:1000, Covance), mouse-anti α-Tubulin (1:5000, Sigma). To control the quality of biochemical fractionation, 10 ml of separated nuclear or membrane fraction respectively were incubated with Hoechst (1:5000) and DiIO16 (0.03 μg/ml, Thermo Fisher) for 10 min on poly-Lysin coated coverslips and imaged on a Zeiss LSM780 inverted confocal microscope.

Image analysis and quantifications

Imaging data was processed and analyzed using ImageJ or Fiji respectively. Kymographs were generated in Fiji. Generally, due to the increasing durations of interphases in the syncytial blastoderm stage, time courses were converted to and plotted as '% interphase completed'. Nuclear surfaces were determined as ellipsoidal approximations by measuring the maximal length of the axis and the nuclear height for each nucleus at every imaged time point for each respective movie. Mean fluorescence intensities of GFP::Nup107, RFP::Nup107 or

fluorescently labeled WGA was determined from manually designed ROIs around the NE or in the cytoplasm in consecutive z-planes of confocal stacks for multiple nuclei in an imaged embryo. Most apical and most basal sections were eliminated for quantifications and the residual planes were used to calculate the mean NE intensities for every nucleus at any given time point. To investigate the potential transfer of fluorescent material from AL-NPCs to NE-NPCs, integrated GFP::Nup107 intensities at the NE or in AL foci respectively were summed over consecutive, 1 μm distant z planes comprising ~10 nuclei. The field of view was kept constant over the time course of recording the respective embryo. Import capacity of nuclei was determined from averaging the ratios of nucleoplasmic to cytoplasmic mean GFP intensities measured in manually designed ROIs from multiple nuclei and adjacent cytoplasmic regions in embryos expressing Ubi::GFP.nls. To quantitatively assess the NE's permeability barrier, kymographs spanning the NE were generated from movies recorded in embryos expressing GFP::Nup107 that have been injected with TRITC labeled Dextran-155kD. Same-length linescans were computed using a custom Fiji plugin on the separated channels of the kymographs. The maximum value of the GFPNup107 along the linescan, representing the position of the NE, was used to align multiple ROIs relative to each other. NE-permeability was then assessed from the dextran-TRITC intensities within 20 pixels nucleoplasmic to the NE. To correct for differences in ROIs or embryos, these intensities were normalized by the mean of the Dextran fluorescence in the 10 pixels cytoplasmic to the NE for each respective measurement. For all quantifications pooled data was presented as means +/- STDV.

Constructs and generation of transgenic flies

To generate EosFP::Seh1 transgenes, the ORF of *Drosophila* Seh1 (Nup44) (CG8722) was cloned into pDONR221 (Life Technologies) using Gateway. A pUASp destination vector containing an EGFP tag upstream a Gateway cassette, obtained from Terence Murphy (Carnegie Institute), was modified by replacing EGFP with mEos-FP. The pUASp-EosFP-Seh1 cassette was used for standard *Drosophila* transgenesis and F_2 transgenes were identified by eye color. Maternal expression was driven by crossing EosFP::Seh1 males to *matatu>GAL4VP16* females. The cDNA from *Drosophila* LBR (CG17952) was obtained from the EST pOT2-LD38760 and cloned into pDONR221 (Life Technologies) and subsequently to pUASp using Gateway. Transgenes were generated using pUASp-LBR in standard transgenesis.

Sub-cellular fractionation and protein identification by mass spectrometry

Approximately 300 mg wet staged *Drosophila* embryos were dechorionated washed and lysed in Lysis buffer (200 mM Sucrose, 10 mM Tris pH 7.5, 25 mM NaCl, 5 mM $MgCl_2$). Nuclei were isolated by centrifugation at 5000 rpm for 13 min and stripped from attached membranes by centrifugation (45 min, 12000 rpm) through a 1 M Sucrose cushion. Microsomal membranes were isolated by spinning the supernatant from the nuclear precipitate at 40000 rpm for 45 min. The remaining supernatant was used as cytosolic fraction. Subsequently, fractions were lysed or supplemented by addition of Rapigest (Waters) and urea to a final concentration of 0.2% (v/v) and 4 M, respectively, and sonicated for 3x 30 sec. Samples were stored at -80°C before being further processed and analyzed by shot-gun mass spectrometry as previously described (Mackmull et al., 2015). The intensity-based absolute quantification (iBAQ) score of the proteinGroups.txt output of MaxQuant was used for further analysis. All comparative analyses were performed using R version 3.0.1. (The R Development Core Team

(2012) R: A Language and Environment for Statistical Computing, http://www.R-project.org/ Ed). Only proteins identified in at least 2 replicates were considered when comparing protein abundances between different fractions. To reduce technical variation data was quantile-normalized using the preprocessCore library (Gentleman et al., 2004). Protein differential expression was evaluated using the Limma package. Differences in protein abundances were statistically determined using the Student's t-test moderated by the empirical Bayes method. Significant regulated proteins were defined by a cut-off of log2 fold change ≤ -1 or ≥ 1 and p-value ≤ 0.01. The proteomics data have been deposited to the ProteomeXchange Consortium (http://proteomecentral.proteomexchange.org) (Vizcaino et al., 2014) via the PRIDE partner repository (Vizcaino et al., 2013) with the dataset identifier PXD004120.

Focused Ion Beam Scanning Electron Microscopy (FIB-SEM)

Dechorionated *Drosophila* embryos were staged by morphological criteria, cryo-immobilized by high-pressure freezing using a HPM010 (Abra Fluid) and freeze substituted at -90°C for 48 h using an EM-AFS2 (Leica Microsystems, Vienna, Austria) in a solution of 1% osmium tetroxide, 0.1% uranyl acetate and 5% water dissolved in anhydrous acetone. The temperature was increased 5°C per hour up to -30°C and the samples incubated for 3 h, followed by warming 5°C per hour up to 20°C and subsequent 5 h incubation. Samples were rinsed in acetone and stepwise infiltrated in durcupan resin (Sigma). Polymerization was done for 48 h at 60°C. Embedded embryos were trimmed and sectioned for TEM and inspected by EM to select ROIs in embryos of the correct cell cycle stage. Positions of ROIs relative to the section were noted and the sample was then trimmed for FIB-SEM. The TEM imaged surface becomes the imaging surface in the FIB-SEM, however the top surface of the sample where the platinum will be deposited needed to also be trimmed (Maco et al., 2014). Using the

position of the ROI relative to the section shape, the block was trimmed with a 90° diamond knife to position the ROI to within 3-10 µm of the block surface. The surface opposite to the exposed ROI was also trimmed to be parallel for mounting to the SEM stub (Agar Scientific). The sample was gold sputter coated (Quorum) and placed into the FIB-SEM for acquisition (Zeiss, Auriga 60). After ROI location a protective 1µm layer of platinum was deposited on the top face above the ROI using a gas deposit system operated under SmartSEM (Carl Zeiss Microscopy GmbH). Acquisition was performed with Atlas3D software (Fibics). Datasets were acquired with 5 nm pixel size and 5 nm steps in z and aligned using TrakEM (ImageJ). Segmentation and isosurface rendering were done in 3dmod 4.5.8.

Correlative Light and Electron Microscopy (CLEM)

For CLEM analysis, the embryos were high-pressure frozen (HPM010 AbraFluid), using 20% dextran as cryoprotectant. The embryos were pierced with a needle in a cryo-microtome chamber (Leica EM FC6) at -160˚C to facilitate freeze substitution (Eltsov et al., 2015). Embryos were then freeze-substituted (EM-AFS2 - Leica Microsystems) with 0.1% Uranyl Acetate (UA) in acetone at -90˚C for 48h. The temperature was then raised to -45˚C at 3.5˚C/h and samples were further incubated for 5h. After rinsing in acetone, the samples were infiltrated in Lowicryl HM20 resin, while raising the temperature to -25˚C and left to polymerize under UV light for 48 hours at -25°C and for further 9 hours while the temperature was gradually raised to 20˚C (5˚C/h). Thick sections (300 nm) were cut from the polymerized resin block and picked up on carbon coated mesh grids. Tomography was performed in 1degree increments at 4700x magnification on a FEI Tecnai F30 electron microscope.

The fluorescence microscopy (FM) imaging of the sections was carried out as previously described (Avinoam et al., 2015; Kukulski et al., 2011) using a widefield fluorescence microscope (Olympus IX81) equipped with an Olympus PlanApo 100X 1.40 NA oil immersion objective. Images were collected with mcherry-specific settings as well as in the green channel, to visualize the autofluorescence of heavily UA-stained mitochondria in the sample, which will serve as anchor points for the correlation.

TEM images were acquired with a FEI CM120 electron microscope. Correlation between light and electron micrographs was carried out with the plugin ec-CLEM (http://icy.bioimageanalysis.org/plugin/ec-CLEM) of the software platform Icy (de Chaumont et al., 2012). The position of mitochondria was manually assigned by clicking on the FM (green channel) and EM images. The coordinate of pairs in the two imaging modalities were used to calculate a linear transformation, which allowed to map the coordinates of the fluorescent spot of interest (red channel) and to overlay it on the electron micrograph.

Transmission Electron Microscopy (TEM)

Drosophila embryos were dechorionated, staged by morphology and cryo-immobilized by high-pressure freezing using a HPM010 (Abra Fluid). Freeze substitution of the embryos was done using a EM-AFS2 (Leica Microsystems, Vienna, Austria) in a solution of 0.3% glutaraldehyde, 0.3% uranyl acetate and 3% water dissolved in anhydrous acetone. The substitution was done at -90°C for 48 h. The temperature was increased at a 5°C per hour up to -45°C and the samples incubated for 16 h. The samples were rinsed in acetone and infiltrated in a graded series of acetone and HM20 resin, polymerization was done under UV light at -45°C for 48 h. The temperature was increased at a 5°C per hour up to 20°C and the polymerization was finished for 24h. For serial EM, the resin-embedded embryos were

trimmed and consecutive 100 nm distant sections were obtained with a section thickness of ~80 nm. Electron micrographs of these sections were recorded on a FEI Tecnai F30 equipped with Gatan US4000 CCD camera, operated at 300 kV or a FEI Biotwin equipped with an Olympus Keen View G2 camera operated at 120kV, respectively.

Supplementary References

Avinoam, O., Schorb, M., Beese, C.J., Briggs, J.A., and Kaksonen, M. (2015). ENDOCYTOSIS. Endocytic sites mature by continuous bending and remodeling of the clathrin coat. Science *348*, 1369-1372.

Cavey, M., and Lecuit, T. (2008). Imaging cellular and molecular dynamics in live embryos using fluorescent proteins. Methods Mol Biol *420*, 219-238.

de Chaumont, F., Dallongeville, S., Chenouard, N., Herve, N., Pop, S., Provoost, T., Meas-Yedid, V., Pankajakshan, P., Lecomte, T., Le Montagner, Y.*, et al.* (2012). Icy: an open bioimage informatics platform for extended reproducible research. Nat Methods *9*, 690-696.

Eltsov, M., Dube, N., Yu, Z., Pasakarnis, L., Haselmann-Weiss, U., Brunner, D., and Frangakis, A.S. (2015). Quantitative analysis of cytoskeletal reorganization during epithelial tissue sealing by large-volume electron tomography. Nat Cell Biol *17*, 605-614.

Gentleman, R.C., Carey, V.J., Bates, D.M., Bolstad, B., Dettling, M., Dudoit, S., Ellis, B., Gautier, L., Ge, Y., Gentry, J.*, et al.* (2004). Bioconductor: open software development for computational biology and bioinformatics. Genome Biol *5*, R80.

Katsani, K.R., Karess, R.E., Dostatni, N., and Doye, V. (2008). In vivo dynamics of Drosophila nuclear envelope components. Mol Biol Cell *19*, 3652-3666.

Kukulski, W., Schorb, M., Welsch, S., Picco, A., Kaksonen, M., and Briggs, J.A. (2011). Correlated fluorescence and 3D electron microscopy with high sensitivity and spatial precision. J Cell Biol *192*, 111-119.

Lenart, P., and Ellenberg, J. (2006). Monitoring the permeability of the nuclear envelope during the cell cycle. Methods (San Diego, Calif) *38*, 17-24.

Mackmull, M.T., Iskar, M., Parca, L., Singer, S., Bork, P., Ori, A., and Beck, M. (2015). Histone Deacetylase Inhibitors (HDACi) Cause the Selective Depletion of Bromodomain Containing Proteins (BCPs). Mol Cell Proteomics *14*, 1350-1360.

Maco, B., Cantoni, M., Holtmaat, A., Kreshuk, A., Hamprecht, F.A., and Knott, G.W. (2014). Semiautomated correlative 3D electron microscopy of in vivo-imaged axons and dendrites. Nat Protoc *9*, 1354-1366.

Mendjan, S., Taipale, M., Kind, J., Holz, H., Gebhardt, P., Schelder, M., Vermeulen, M., Buscaino, A., Duncan, K., Mueller, J.*, et al.* (2006). Nuclear pore components are involved in the transcriptional regulation of dosage compensation in Drosophila. Mol Cell *21*, 811-823.

Vizcaino, J.A., Cote, R.G., Csordas, A., Dianes, J.A., Fabregat, A., Foster, J.M., Griss, J., Alpi, E., Birim, M., Contell, J.*, et al.* (2013). The PRoteomics IDEntifications (PRIDE) database and associated tools: status in 2013. Nucleic Acids Res *41*, D1063-1069.

Vizcaino, J.A., Deutsch, E.W., Wang, R., Csordas, A., Reisinger, F., Rios, D., Dianes, J.A., Sun, Z., Farrah, T., Bandeira, N., *et al.* (2014). ProteomeXchange provides globally coordinated proteomics data submission and dissemination. Nat Biotechnol *32*, 223-226.
von Appen, A., Kosinski, J., Sparks, L., Ori, A., DiGuilio, A.L., Vollmer, B., Mackmull, M.T., Banterle, N., Parca, L., Kastritis, P., *et al.* (2015). In situ structural analysis of the human nuclear pore complex. Nature *526*, 140-143.
Wagner, N., Weber, D., Seitz, S., and Krohne, G. (2004). The lamin B receptor of Drosophila melanogaster. J Cell Sci *117*, 2015-2028.
