# Supplementary figures and images for: Pre-assembled Nuclear Pores Insert into the Nuclear Envelope during Early Development
Source: Cell. 2016 Jul 28;166(3):664–78. doi: 10.1016/j.cell.2016.06.015 (PMC4967450; doi:10.1016/j.cell.2016.06.015)

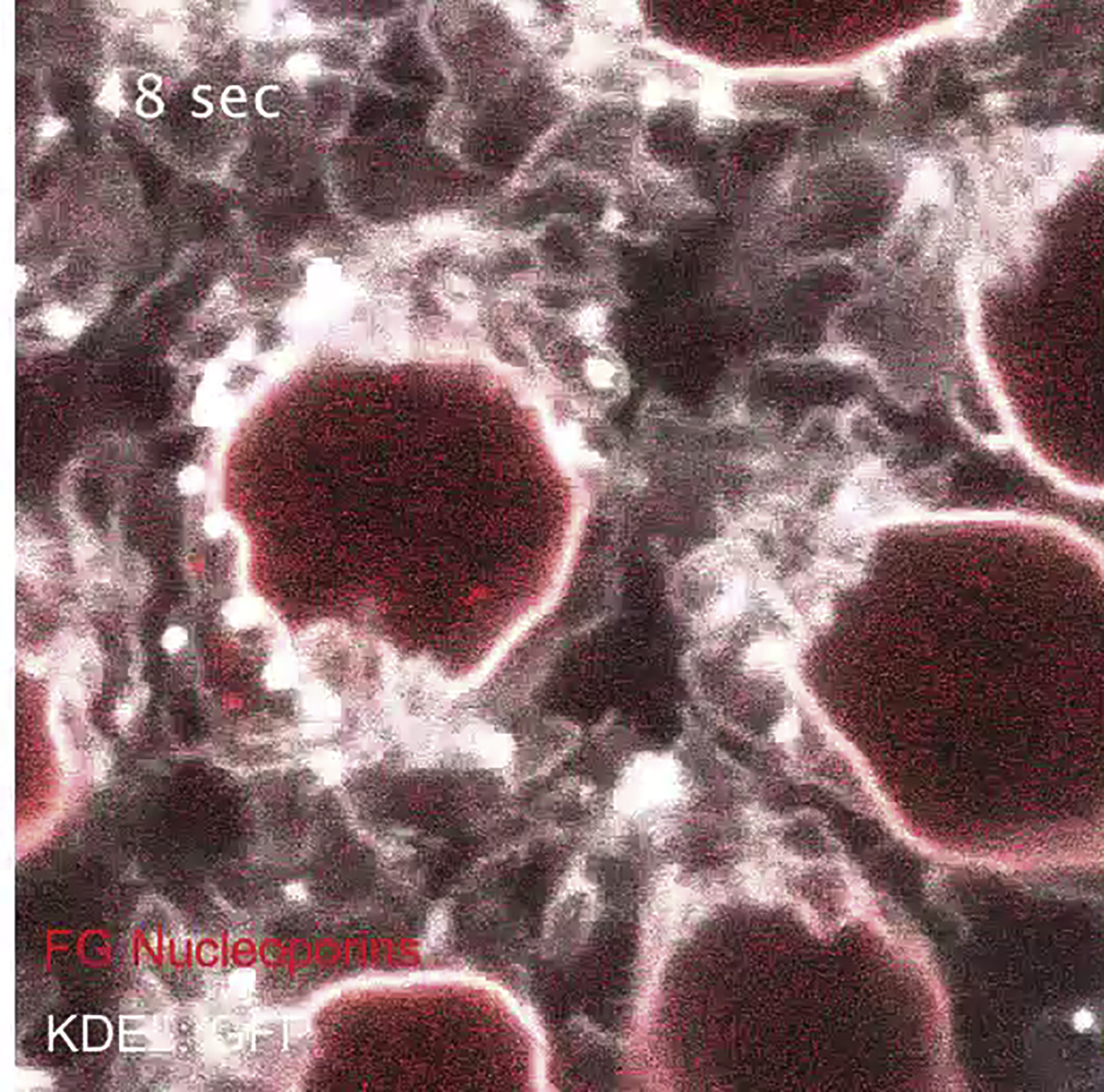

Supplement: Movie S1. AL Assemble along ER Membranes, Related to Figure 1 — Syncytial blastoderm Drosophila embryo expressing KDEL::GFP, injected with Alexa555-WGA labeling FG-Nups, imaged from the top during mitosis. WGA becomes soluble as NPCs dis-assemble in prophase and re-concentrates around daughter nuclei and at AL-NPCs along ER membranes in telophase. One frame was imaged every 6 seconds. [file mmc2.jpg]

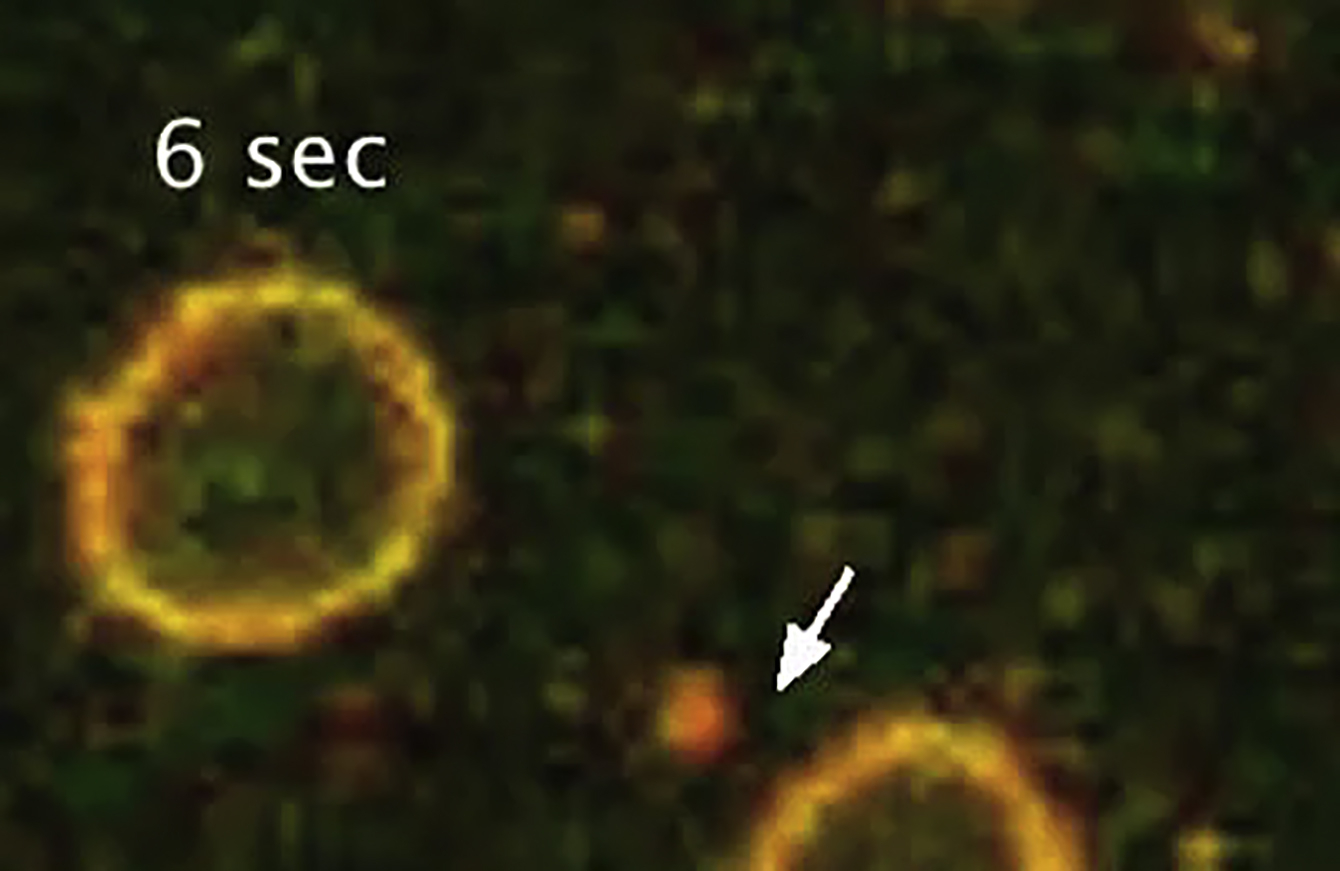

Supplement: Movie S2. AL-NPCs Insert to the NE during Interphase, Related to Figure 1 — Top-view time lapse movie of a syncytial blastoderm Drosophila embryo expressing GFP::Nup107 injected with Alexa555-WGA. The Y-complex protein Nup107 and FG Nups co-localize at AL-NPCs as they insert to the NE (arrow). Stills and a kymograph of this movie are shown in Figures 1E and 1F. The movie was imaged at 3 frames / second. [file mmc3.jpg]

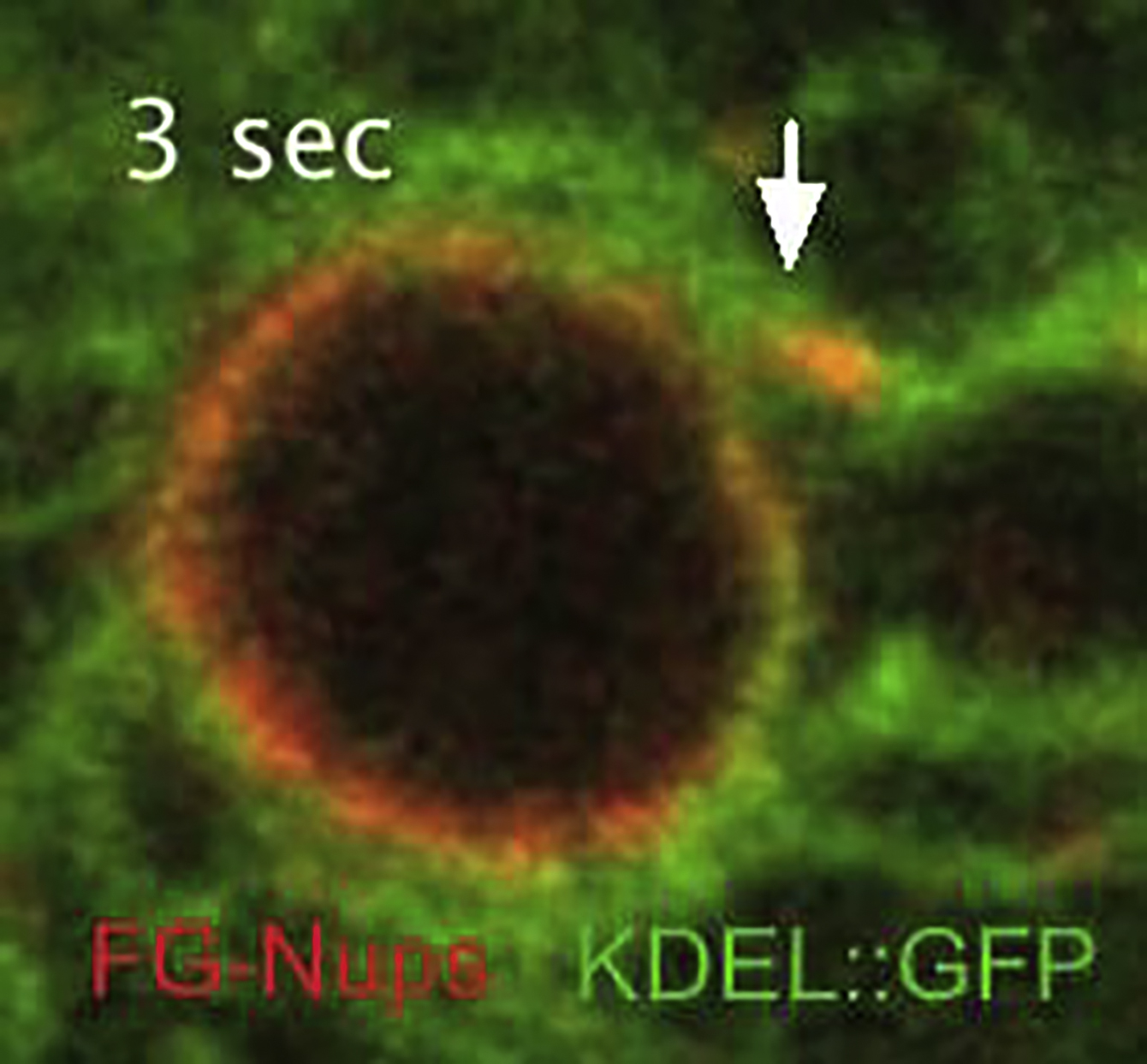

Supplement: Movie S3. AL-NPCs Insert along Membranes to the NE, Related to Figure 1 — Top-view time lapse movie of a syncytial blastoderm Drosophila embryo expressing GFP::Nup107 injected with Alexa555-WGA. AL-NPCs insertion to the NE (arrow) occurs on ER membranes. The embryo was imaged at 1 frame / second. [file mmc4.jpg]

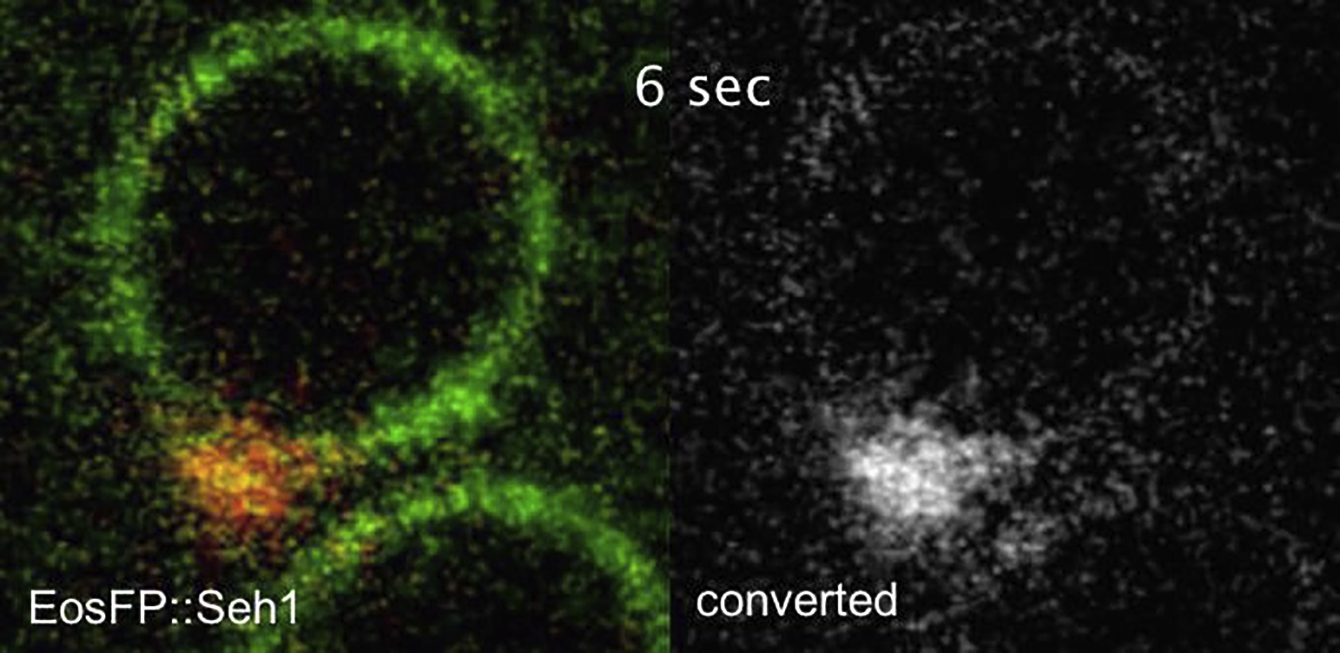

Supplement: Movie S4. Uptake of AL-NPCs to the NE, Related to Figure 1 — Time lapse movie of a syncytial blastoderm Drosophila embryo expressing EosFP::Seh1 during interphase. Photo-conversion was performed at an AL-NPC spot adjacent to the NE, 6 seconds after start of the movie. Panels show the combined channels (left) and the photoconverted channel (right). Note that lateral dissipation of the converted EosFP::Seh1 starts only about 100 seconds after photo-conversion. Stills and a kymograph of this movie are shown in Figures 1G–1G″. The embryo was imaged at 1 frame every seconds. [file mmc5.jpg]

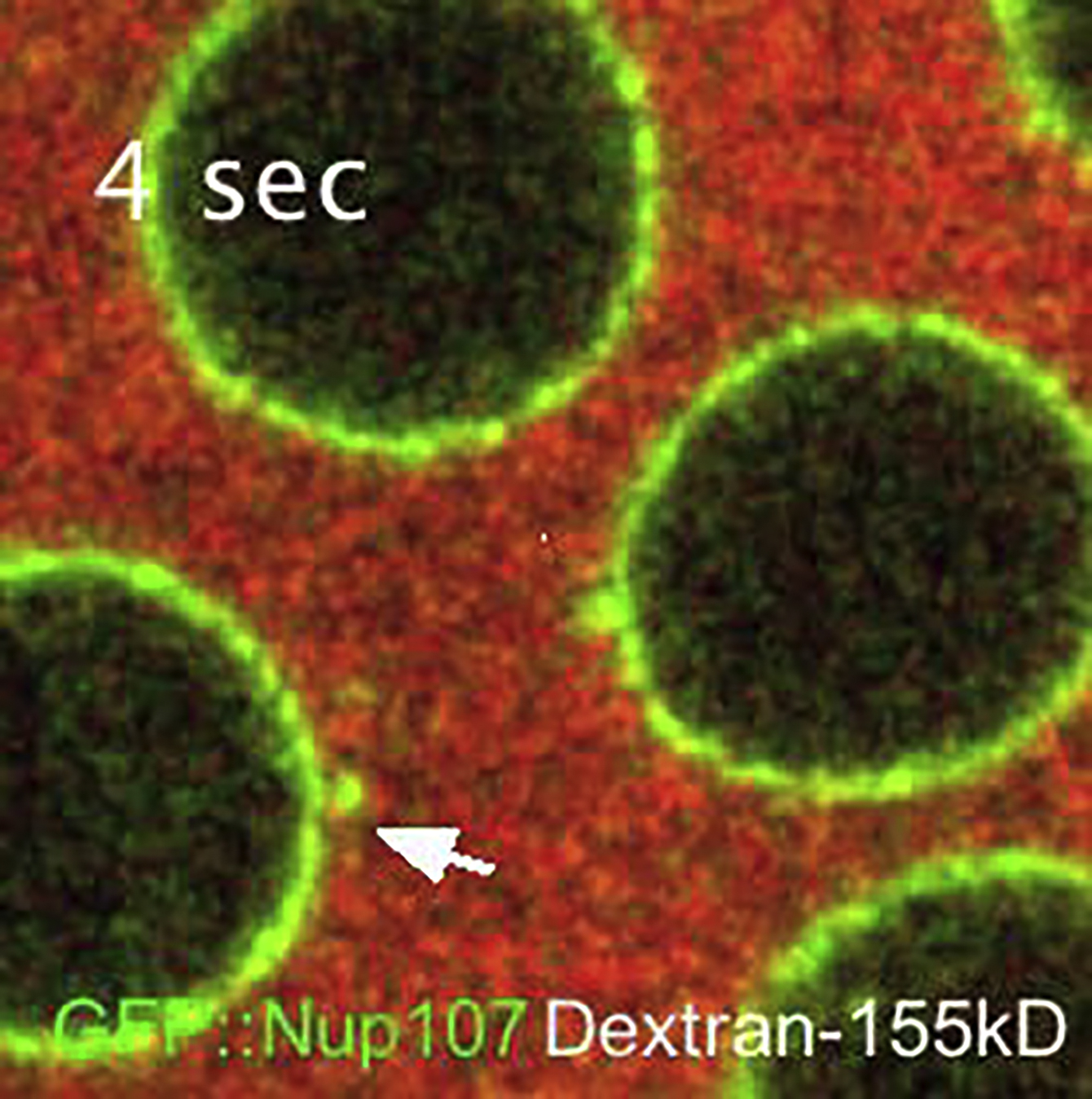

Supplement: Movie S5. The Permeability Barrier of the NE Is Maintained upon AL Insertion, Related to Figure 5 — Top view time lapse movie of a syncytial blastoderm Drosophila embryo expressing GFP::Nup107 injected with TRITC conjugated Dextran-155kD during interphase. Dextran-155kD is excluded from nuclei, as GFP::Nup107 labeled AL-NPCs insert to the NE (arrow). The movie was acquired at 1 frame / second. [file mmc6.jpg]

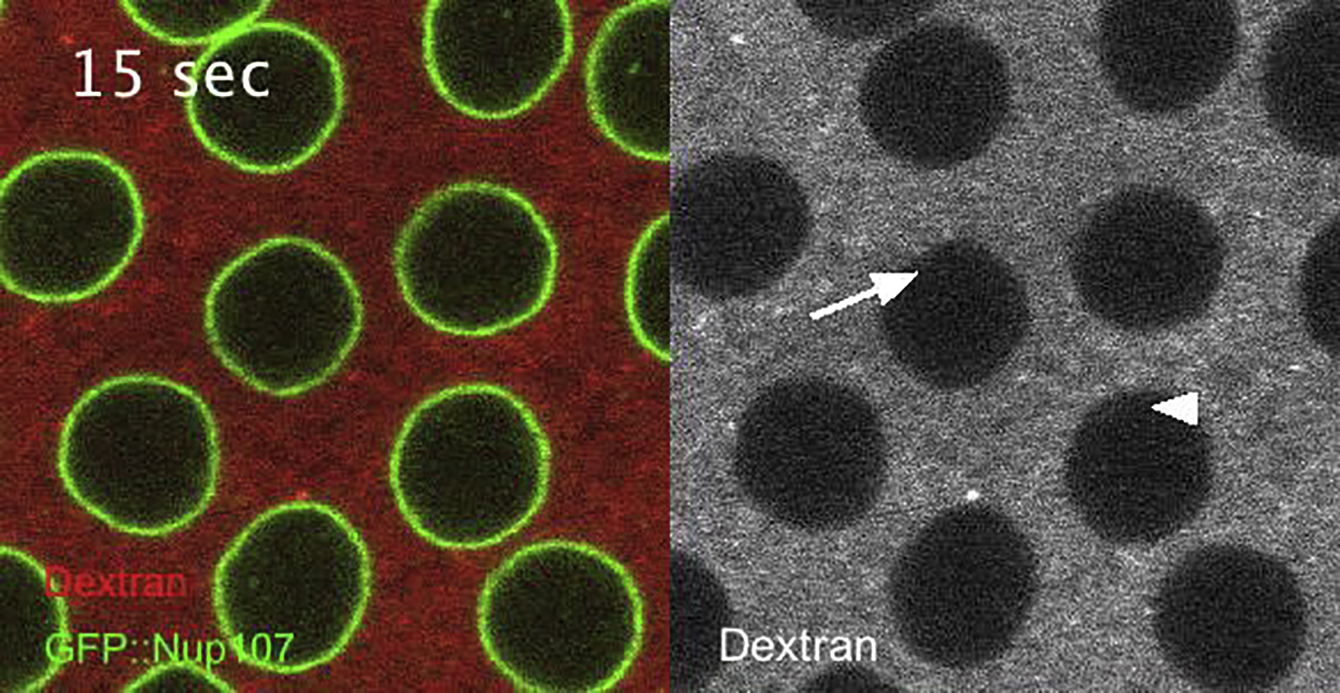

Supplement: Movie S6. Laser Puncture of the NE Impairs Its Permeability Barrier, Related to Figure 5 — Top view time lapse movie of a syncytial blastoderm Drosophila embryo expressing GFP::Nup107 injected with TRITC conjugated Dextran-155kD during interphase. Dextran is excluded from nuclei, but fills the nucleoplasm after laser-induced puncture of the NE of two nuclei 25 seconds after the movie starts. Targeted nuclei are indicated by an arrow and an arrowhead, respectively. Quantitative analysis of this movie is shown in Figures 5C–5C″. Note the strong mechanical response of nuclei after puncture, indicated by irregular NE jiggling. A frame was imaged every 5 seconds. [file mmc7.jpg]
