# Supplementary material for: Pre-assembled Nuclear Pores Insert into the Nuclear Envelope during Early Development
Source: Cell. 2016 Jul 28;166(3):664–78. doi: 10.1016/j.cell.2016.06.015 (PMC4967450; doi:10.1016/j.cell.2016.06.015)
Supplement: Document S2. Article plus Supplemental Information [file mmc8.pdf]

Cell

Article

Pre-assembled Nuclear Pores Insert into the Nuclear Envelope during Early Development

Graphical Abstract

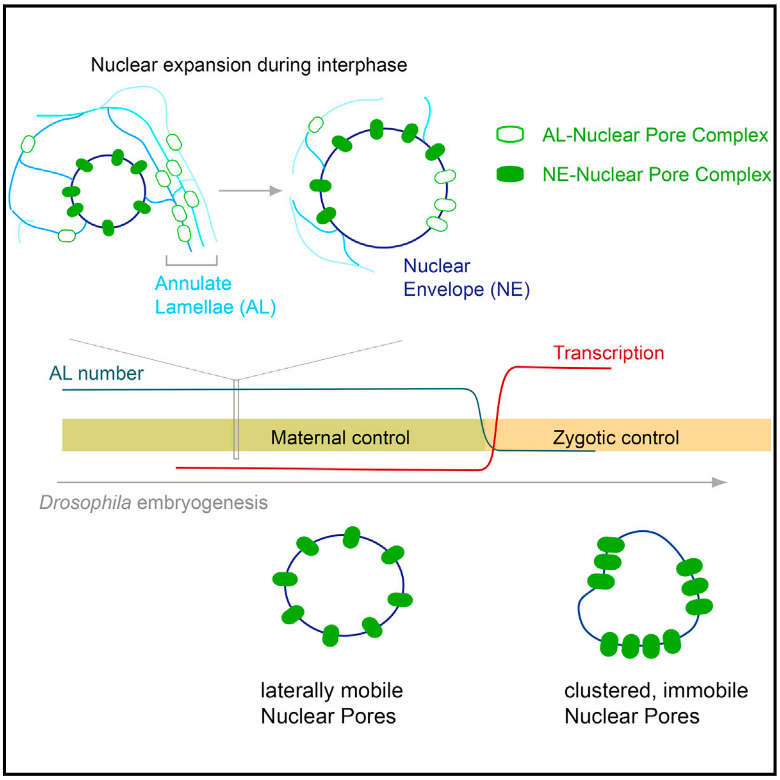

Authors

Bernhard Hampoelz, Marie-Therese Mackmull, Pedro Machado, ..., Thomas Lecuit, Yannick Schwab, Martin Beck

Correspondence

martin.beck@embl.de

In Brief

Rapidly growing embryos meet the challenge of stocking the nuclear envelope with nuclear pore complexes by keeping a ready store of pores in stacked membranous rings that feed into the envelope without puncturing it.

Highlights

- Annulate lamellae (AL) NPCs insert into the nuclear envelope during interphase
- AL-NPCs are pore scaffolds devoid of most transport channel nucleoporins
- NE-openings enable AL insertion, yet the permeability barrier remains unperturbed
- AL-NPC insertion operates only before gastrulation

CrossMark

Hampoelz et al., 2016, Cell *166*, 664–678
July 28, 2016 © 2016 The Author(s). Published by Elsevier Inc.
http://dx.doi.org/10.1016/j.cell.2016.06.015

CellPress

Article

Pre-assembled Nuclear Pores Insert into the Nuclear Envelope during Early Development

Bernhard Hampoelz,[1] Marie-Therese Mackmull,[1] Pedro Machado,[2] Paolo Ronchi,[2] Khanh Huy Bui,[1,5] Nicole Schieber,[4] Rachel Santarella-Mellwig,[2] Aleksandar Necakov,[1] Amparo Andrés-Pons,[1] Jean Marc Philippe,[3] Thomas Lecuit,[3] Yannick Schwab,[2,4] and Martin Beck[1,4,*]
[1]European Molecular Biology Laboratory, Structural and Computational Biology Unit, 69117 Heidelberg, Germany
[2]European Molecular Biology Laboratory, Electron Microscopy Core Facility, 69117 Heidelberg, Germany
[3]Aix-Marseille Université, CNRS, IBDM UMR 7288, 13009 Marseille, France
[4]European Molecular Biology Laboratory, Cell Biology and Biophysics Unit, 69117 Heidelberg, Germany
[5]Present address: Department of Anatomy and Cell Biology, Groupe de Recherche Axé sur la Structure des Protéines (GRASP), McGill University, Montreal, Quebec H3A 0C7, Canada
*Correspondence: martin.beck@embl.de
http://dx.doi.org/10.1016/j.cell.2016.06.015

SUMMARY

Nuclear pore complexes (NPCs) span the nuclear envelope (NE) and mediate nucleocytoplasmic transport. In metazoan oocytes and early embryos, NPCs reside not only within the NE, but also at some endoplasmic reticulum (ER) membrane sheets, termed annulate lamellae (AL). Although a role for AL as NPC storage pools has been discussed, it remains controversial whether and how they contribute to the NPC density at the NE. Here, we show that AL insert into the NE as the ER feeds rapid nuclear expansion in *Drosophila* blastoderm embryos. We demonstrate that NPCs within AL resemble pore scaffolds that mature only upon insertion into the NE. We delineate a topological model in which NE openings are critical for AL uptake that nevertheless occurs without compromising the permeability barrier of the NE. We finally show that this unanticipated mode of pore insertion is developmentally regulated and operates prior to gastrulation.

INTRODUCTION

In eukaryotes, the double membranous nuclear envelope (NE) encloses the nucleoplasm and separates it from the cytoplasm. The inner nuclear membrane (INM) provides contact with chromatin and the outer nuclear membrane (ONM) is continuous with the endoplasmic reticulum (ER). The two bilayers are fused at nuclear pore complexes (NPCs) that form aqueous channels through which regulated transport of macromolecules occurs. NPCs consist of multiple copies of ~30 different nucleoporins (Nups) that are organized into biochemically distinct sub-complexes (Figures S1A, S1A′, and S1B). Two such modules, the inner ring complex (also called Nup93 complex) and the Y-complex (also called Nup107 complex) constitute the NPC scaffold that is symmetric across the NE plane. FG-Nups (containing phenylalanine-glycine rich intrinsically disordered protein domains) dock onto the scaffold. They constitute the permeability barrier and interact with translocating cargo complexes. Some of them (e.g., Nup214/88, Nup358 [RanBP2], and Nup153) introduce asymmetry by specifically binding to the cytoplasmic or nuclear face of the NPC, respectively (reviewed in Grossman et al., 2012) (Figure S1B).

Obviously, the sheer size and compositional complexity of NPCs renders its assembly and membrane insertion a very intricate task. Two distinct NPC assembly pathways that are temporally separated during the cell cycle have been described. First, during interphase, NPCs are assembled de novo onto an enclosed NE (D'Angelo et al., 2006). Interphase assembly occurs ubiquitously throughout eukaryotes and strictly requires the fusion of the INM and ONM by a mechanism that is only partially understood (Doucet and Hetzer, 2010). Second, no membrane fusion is required for NPC assembly at mitotic exit. This so-called postmitotic assembly mode is restricted to eukaryotes that disassemble their NPCs during mitosis into soluble sub-complexes after phosphorylation by mitotic kinases (Laurell et al., 2011). In anaphase, de-phosphorylation of Nups is thought to trigger the ordered re-assembly onto the separated chromatids before or while membranes enclose daughter nuclei (Doucet et al., 2010; Dultz and Ellenberg, 2010; Dultz et al., 2008). Both insertion mechanisms rely on the stepwise recruitment of pre-assembled sub-complexes. An insertion of pre-assembled NPCs into the NE has (to the best of our knowledge) not yet been described.

NPCs not only reside within the NE but are also found in stacked cytoplasmic membranes termed annulate lamellae (AL) that are a subdomain of the ER (Figure S1C) (Cordes et al., 1996; Daigle et al., 2001). Based on two-dimensional (2D) transmission electron micrographs these membrane stacks have been perceived as parallel membrane sheets decorated with NPCs (hereafter called AL-NPCs) that morphologically appear similar to their counterparts on the nuclear envelope (NE-NPCs) (Kessel, 1983). AL appear in some but not all transformed cell lines (Cordes et al., 1996; Daigle et al., 2001) and are highly abundant in germ cells and early embryos throughout

 © 2016 The Author(s). Published by Elsevier Inc.
This is an open access article under the CC BY license (http://creativecommons.org/licenses/by/4.0/).

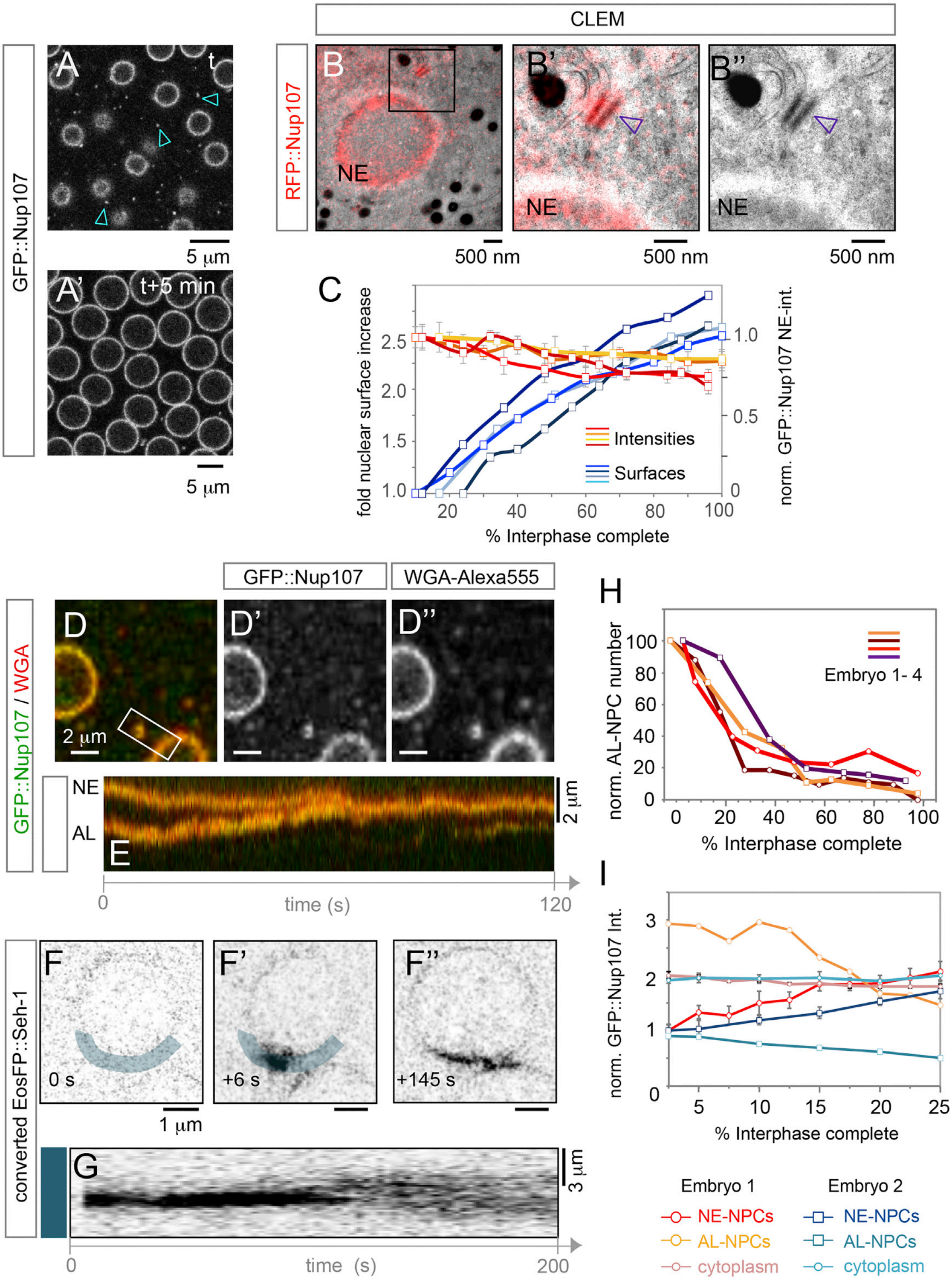

Figure 1. AL-NPCs Insert into the Nuclear Envelope

(A and A′) Nuclear growth and NPC distribution during interphase in the *Drosophila* syncytium: Stills from a time-lapse movie recorded from a GFP::Nup107 expressing embryo imaged right after interphase onset (A) and 5 min later (A′). GFP::Nup107 localizes to the NE and to cytoplasmic foci (arrowheads) in (A), which disappear at t + 5 min (A′).

(B–B″) Cytoplasmic foci of Nup107 fluorescence localize to AL-NPCs. Correlative light and electron microscopy (CLEM) of a RFP::Nup107 expressing interphase embryo. RFP::Nup107 is concentrated along the NE and at foci (boxed in B), that correspond to NPCs along ER membranes (arrowheads in B′ and B″).

(legend continued on next page)

animal phyla, including *Xenopus*, *Caenorhabditis elegans*, sea urchin, *Drosophila*, and also humans (Soupart and Strong, 1974). A role of AL as a storage compartment for maternally deposited Nups that can be made available for meiosis and the rapid cell cycles during early embryogenesis has been suggested (Lénárt and Ellenberg, 2003; Longo and Anderson, 1968; Spindler and Hemleben, 1982) but not experimentally proven. Despite these fundamental and long-standing pretensions the function of AL remains elusive and controversial, primarily for two reasons: (1) it has been difficult to conceive how the insertion of parallel stacked membrane sheets containing pre-assembled and possibly pre-oriented NPCs is topologically possible; and (2) direct experimental evidence for a contribution of AL-NPCs to the pool of NE-NPCs has never been obtained. On the contrary a previous study in *Drosophila* embryos has detected large soluble pools of transport channel Nups and concluded that NPC insertion likely proceeds from soluble cytosolic Nups (Onischenko et al., 2004).

Here, we address the function of AL in the physiological context of the *Drosophila* blastoderm embryo that is rich in AL, while it undergoes a series of 13 synchronized mitoses in a syncytium (Figure S2A). Subsequently, the plasma membranes enclose the cortically aligned somatic nuclei in the extended 14th interphase, forming the first epithelial cell layer before the embryo initiates gastrulation (Schejter and Wieschaus, 1993). This occurs concomitantly with the broad onset of transcriptional activity on the zygotic genome, a major developmental transition present in all metazoan (Newport and Kirschner, 1982). In the syncytial blastoderm, cell-cycle progression is very rapid, with interphase durations of ~10 min during the early cell cycles. At least in mammalian cells, de novo NPC interphase assembly has been described to proceed with markedly slower kinetics (Dultz and Ellenberg, 2010). This led us to hypothesize that NPC assembly into a closed NE in *Drosophila* embryos might occur by a different, faster mechanism. By tracking NPCs in living embryos, we demonstrate direct uptake of AL-NPCs into the NE, as the ER feeds nuclear expansion. We derive a topological model that explains how the INM becomes continuous with inserting membrane sheets from the ER. We conclude that AL insertion to the NE is a previously unanticipated mode of NPC insertion that relies on pre-assembled, yet immature NPC scaffolds and operates prior to gastrulation.

RESULTS

Nuclear Pores Insert from the ER into the NE

To investigate whether AL-NPCs contribute to the pool of NE-NPCs, we conducted live-imaging experiments in *Drosophila* blastoderm embryos before formation of the first somatic cell layer (Figure S2A). During this stage of development, AL are abundant and thus could potentially serve as a reservoir for NE-NPCs. To track NPCs throughout early embryogenesis, we expressed functional GFP or RFP fusions of Nup107 (Katsani et al., 2008) to image scaffold Nups and injected sub-critical concentrations of the fluorescently labeled lectin wheat germ agglutinin (WGA) to label FG-Nups. In interphase, GFP::Nup107 localized to the NE and to prominent foci throughout the cytoplasm (Figure 1A), similar to structures that were previously characterized as AL (Cordes et al., 1996; Daigle et al., 2001; Onischenko et al., 2004, 2005). As expected these foci also stained positive for transport channel Nups (Figures 1D–1D″) and always localized to membranes (Figures S2C–S2E″). To further confirm that these foci are morphologically identical to AL, we performed correlative light and electron microscopy. RFP::Nup107 fluorescence was strongly enriched along the NE and at AL (Figures 1B–1B″, S2B, and S2B′). We conclude that fluorescence imaging in life embryos is well-suited to study the spatiotemporal dynamics of annulate lamellae.

Image quantification revealed a 2.5- to 3-fold increase in nuclear surface during interphases, indicating a considerable uptake of ER membranes within a few minutes (Figures 1A, 1A′, and 1C). Thus, the surface area of the nucleus just before division is more than twice as large as the combined surfaces of the two daughter nuclei after division. This finding implies an excess of nucleoporins with respect to the available nuclear surface after mitosis. Both NE-NPCs and AL-NPCs disassemble during mitosis (Cordes et al., 1996; Onischenko et al., 2005; Stafstrom and Staehelin, 1984b). This leaves a fenestrated NE that, unlike in vertebrates, stays throughout mitosis around the separating sister chromatids and the mitotic spindle, except at centrosomes

(C) NPC density stays constant as nuclei grow. Quantification of normalized nuclear surface increase (blue curves) and the mean GFP::Nup107 fluorescence intensity ± SD at the NE (red curves) during interphases. Values on both graphs are normalized to the earliest measured time point for each movie (n = 71 nuclei in four embryos).

(D–E) The Y-complex protein Nup107 and WGA-labeled transport Nups co-localize at the NE and at AL-NPCs that insert to the NE. Top view still (D–D″) and kymograph (E) from a time-lapse movie imaging a WGA-Alexa555 injected syncytial blastoderm embryo expressing GFP::Nup107 (see also Movie S2). Insertion is captured in the kymograph (E) that spans the region of interest (ROI) boxed in (D).

(F–G) Stills (F–F″) and kymograph (G) of the blue shaded ROI from a time-lapse movie recording photo-converted EosFP::Seh-1 before (0 s, F) or after (6 s, F′, and 145 s, F″) photo-conversion of AL-NPCs adjacent to the NE. NPC transfer from AL to the NE is documented by the lateral dispersion of the converted signal, which starts after ~100 s (G).

(H) AL-NPC number drops during early interphases. Quantification of the relative AL-NPC number inferred from GFP::Nup107 fluorescence for four embryos live-imaged over interphases. AL-NPCs were counted from 1 μm distant z sections spanning nuclear height in a constant field of view comprising ~10 nuclei for each embryo.

(I) GFP::Nup107 fluorescence intensity shifts from AL to the NE in the first 25% of interphases, when AL number drops the most (H). Fluorescence intensities were integrated over consecutive confocal slices covering the entire nuclear height at the NE (NE-NPCs) and at AL (AL-NPCs). Cytoplasmic GFP::Nup107 was determined from the mean fluorescence intensity. Analysis was done on two embryos (n = 13 and 11 nuclei, respectively). See Figure 1A for representative image; error bars represent ± SD over multiple ROIs. All images in Figure 1 are acquired from embryos in cycles 10–13 of the syncytial blastoderm stage.
See also Figure S1 and Movies S2 and S4.

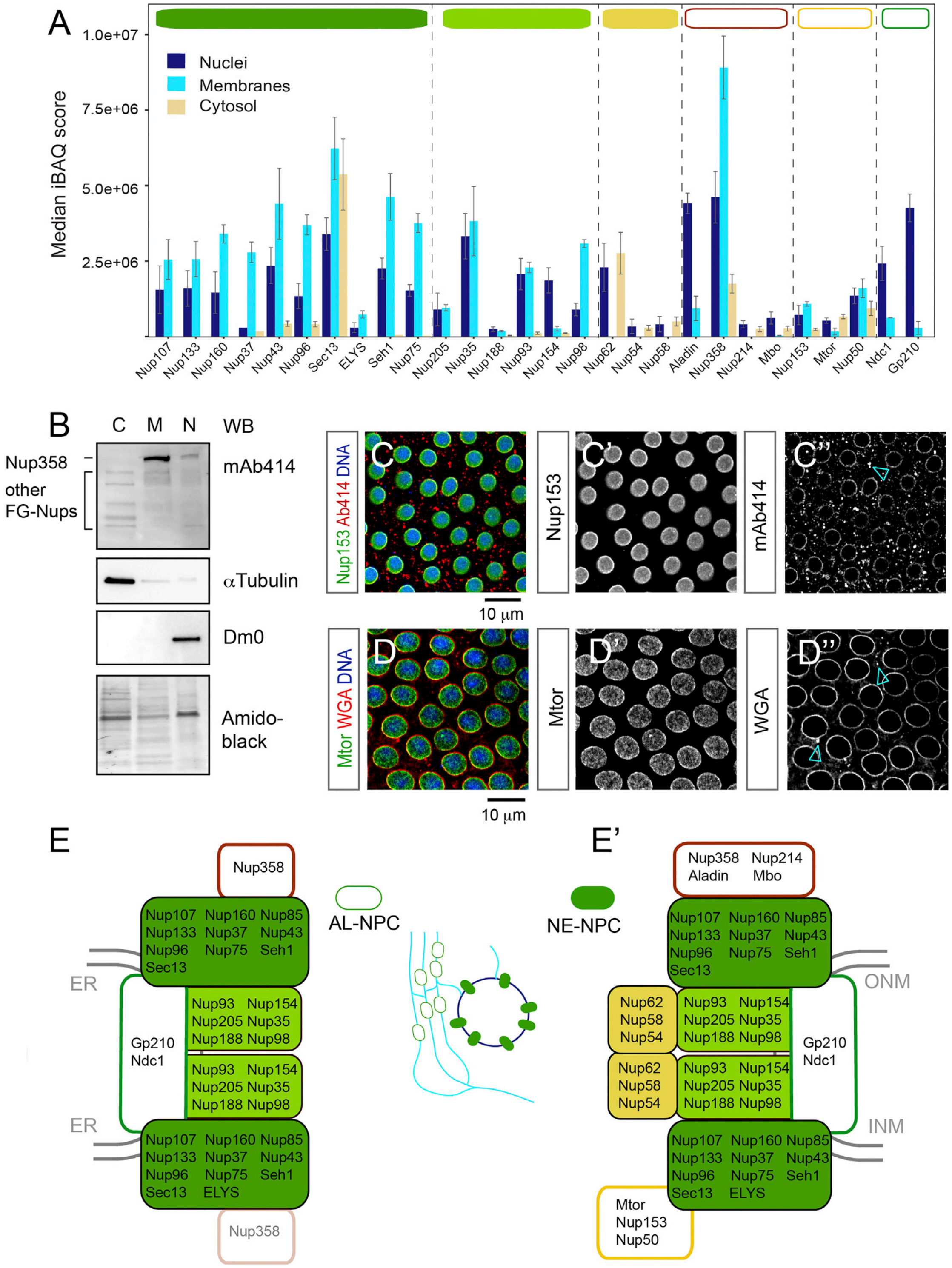

Figure 2. AL-NPCs Resemble Pore Scaffolds

(A) Composition of NE-NPCs and AL-NPCs. Median intensity-based absolute quantification (iBAQ) scores of Nups detected in nuclei containing NE-NPCs, microsomal membranes containing AL-NPCs and cytosol after fractionation of *Drosophila* embryos in the syncytial blastoderm stage (n = 3 biological replicates). Nups are grouped into known subcomplexes and color-coded as represented in (E′).

(B) Western blot analysis of fractionated *Drosophila* syncytial blastoderm embryos. The Lamin Dm0 is exclusively nuclear (N), while α-tubulin is strongly enriched in the cytoplasm (C), confirming fractionation quality. Detection with mAb414 recognizing a panel of FG-Nups reveals Nup358 predominantly in membranes (M) and nuclei but absent from the cytosol. Other FG Nups are mostly soluble (see text). Amido black shows equal loading.

(legend continued on next page)

(Movie S1). Simultaneous with NE-NPC re-assembly at the daughter nuclei in late mitosis, AL-NPCs appeared first at membranes at the spindle (Figures S2C–S2C″; Movie S1), consistent with the idea of excess nucleoporins at late mitosis/early interphase.

To address if AL-NPCs contribute to the NE-NPC pool during the following interphase, we first determined if NE-NPC density decreases during nuclear surface expansion. We quantified the mean fluorescence intensities of GFP::Nup107 at the NE and found that it stayed almost constant (Figure 1C). As a consequence, NPCs have to insert constantly into the NE as its surface increases. Conversely, AL-NPCs were highly abundant in early but not late interphase (Figures 1A and 1A′). We therefore investigated their fate during interphase progression by tracking AL-NPCs in living embryos and found that they insert into the NE (Figures 1D–1G and S2E–S2E″; Movies S2 and S3), along ER membranes (Figures S2E–S2E″; Movie S3). To directly confirm the transfer of Nups from the observed cytoplasmic foci to the NE, we imaged embryos expressing photo-convertible Seh1-EosFP. After photo-conversion of a fluorescent spot close to a nucleus, the signal remained locally constrained for ~100 s before it laterally resolved into the proximal NE over roughly the same time frame (Figures 1F–1F″ and 1G; Movie S4), suggesting a critical event prior to lateral diffusion. We conclude that AL-NPCs insert into the NE during blastoderm interphases.

Previous EM-based morphometry suggested that the total number of AL-NPCs stays constant in the syncytial blastoderm, i.e., during the first 90 min of *Drosophila* embryogenesis (Onischenko et al., 2004). However, if AL-NPCs considerably contribute to the pool of NE-NPCs by inserting into the NE while it expands, their number should decrease at least temporarily on much shorter timescales, namely during the ~10 min of each interphase. Indeed, we found that AL-NPCs diminished as interphases progressed (Figure 1H). This reduction was particularly strong in the first half of interphase, when the rate of nuclear growth and thus NPC insertion was highest (Figure 1C). To estimate if the reduction of AL-NPC number reflects NPC redistribution from AL to the NE, we measured the respective GFP::Nup107 fluorescence levels at both compartments in three dimensions over time. For all quantified nuclei, the integrated NE fluorescence intensities of GFP::Nup107 increased between 70% and 100% within the first quarter of interphase, while inversely intensities at AL strongly decreased, mirroring AL disappearance (Figure 1I). This supports a scenario in which the pool of NE-NPCs is predominantly fed by integration of AL-NPCs. Alternatively, AL could disassemble and soluble Nups could add to pore formation at the growing NE from the cytoplasm. Yet this is unlikely since the GFP::Nup107 background intensity in the cytoplasm remained constant as AL disassembled (Figure 1I). We conclude that our data rather support a scenario in which pre-assembled NPCs insert from AL into the NE.

There are, however, major impediments that challenge the notion that intact NPC can insert into the NE: first, NPCs have an inherent compositional directionality across the NE plane (Figure S1B). If AL-NPCs were identical to NE-NPCs, they would be assembled asymmetrically in the absence of a nuclear compartment providing a directionality cue. They also had to be inserted into the NE in the correct orientation. Second, the integration of NPC-containing ER sheets into an intact NE poses striking topological obstacles. In particular, how an AL membrane sheet can become continuous with the INM of a sealed, intact NE is far from obvious. How is AL-NPC insertion thus possible?

AL-NPCs Resemble Pore Scaffolds

The asymmetry of NPCs derives from sets of FG-Nups that are found exclusively either at the nucleoplasmic or cytoplasmic side of the NPC, in contrast to the symmetrically embedded scaffold Nups of the inner ring and Y-complexes (Figures S1A and S1B). We therefore explored if NPC composition was preserved in AL. We subjected blastoderm embryos to subcellular fractionation and comparatively analyzed fractions enriched for nuclei, microsomal membranes containing AL (devoid of the NE) and soluble cytosolic proteins by quantitative mass spectrometry (Figures S3A–S3D″, S4A, and S4B). We found that AL-NPCs contain the full set of NPC scaffold components, namely all the members of the inner ring and Y-complexes (Figures 2A and S4B). In contrast, their levels were low or undetectable in the cytosol, with the exception of Sec13, a known member of the cytosolic coatomer complex (Fath et al., 2007) (Figures 2A and S4B). These data further support the above-proposed scenario in which soluble pools of scaffold Nups cannot significantly contribute to the maintenance of NE-NPC number during interphase (see also Figure 1I).

The FG-Nups 358 and 98 displayed a subcellular distribution that was similar to scaffold Nups (Figures 2A and S4B). Both have been recently shown to critically contribute to NPC scaffold formation (Fischer et al., 2015; Stuwe et al., 2015; von Appen et al., 2015). Notably, the presence of Nup98 in AL-NPCs, a protein that is the essential constituent of the NPC permeability barrier (Hülsmann et al., 2012), suggests that NPCs are impermeable for larger molecules at all times. In contrast, the members of the Nup62/58/54 and Nup214/88 (the latter called Mbo in flies) complexes as well as the nuclear basket components Tpr (called Mtor in flies) and Nup153 were absent in AL-NPCs (Figures 2A–2D and S4B). Instead, the Nup214/88 and Nup62/58/54 complexes were highly abundant in the cytosol (Figures 2A, 2B, and S4B), in agreement with previous biochemical results that identified certain FG-Nups to be predominantly soluble and excluded from ER-membranes (Onischenko et al., 2004).

(C–D″) Top views onto a fixed *Drosophila* syncytial blastoderm embryo in interphase. The nuclear basket components Nup153 (C and C′) and Mtor (D and D′) are absent from AL-NPCs (arrowheads in C″, D″), which stain positive for mAb414 (C and C″) or WGA (D and D″), both labeling FG-Nups (including Nup358).
(E and E′) AL-NPCs (E) are pore scaffolds made of transmembrane Nups, the inner ring, and Y-complex nucleoporins, extended by Nup358, which might or might not be attached symmetrically across ER membranes. NE-NPCs (E′) recruit soluble Nups to construct the mature pore that is asymmetric across the NE.
See also Figures S3 and S4.

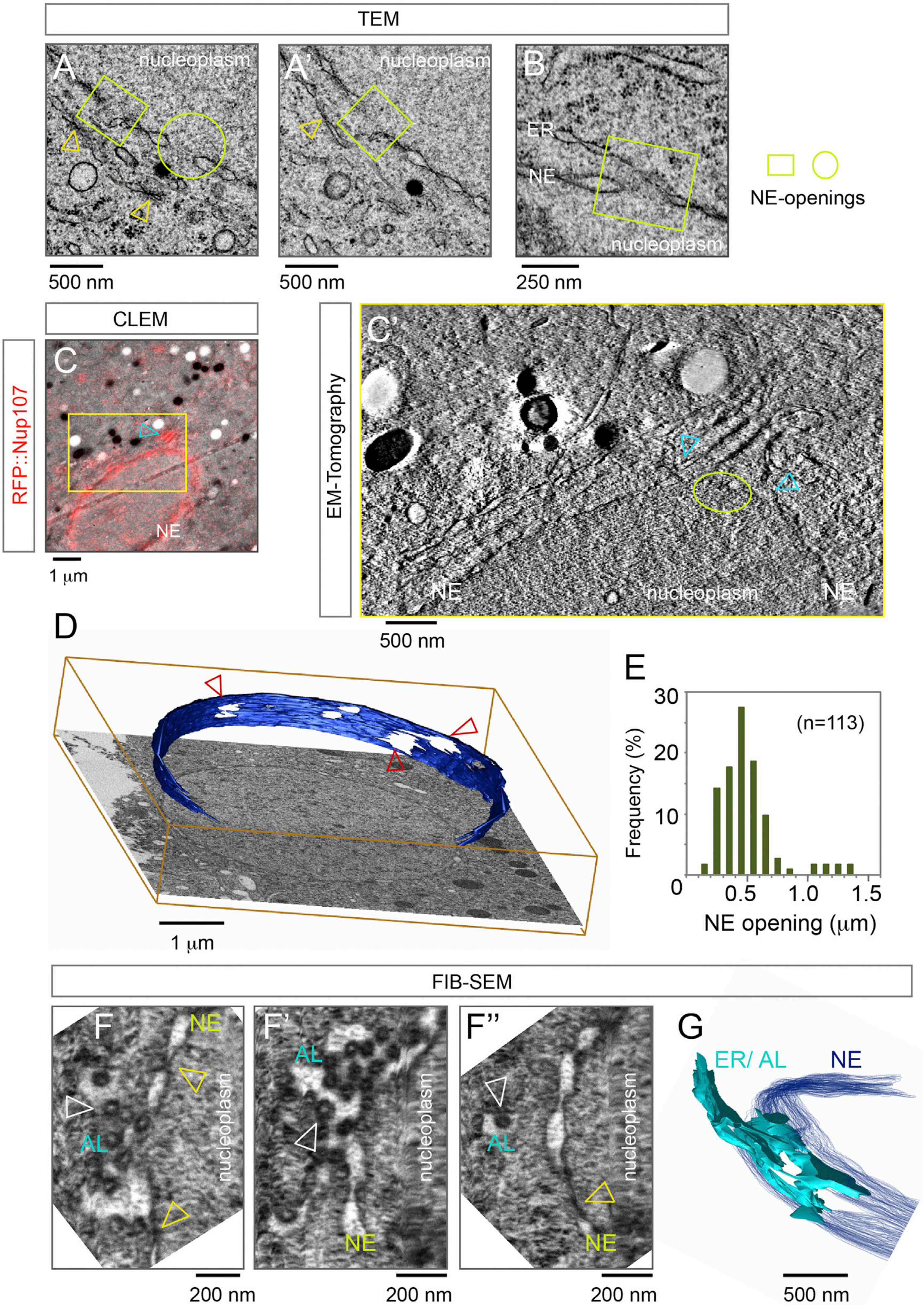

(legend on next page)

One might thus surmise that NPC assembly is completed after insertion into the NE by recruiting soluble Nups from the cytosol in order to establish directionality and transport competence. Indeed, the nuclear accumulation of nls::GFP was delayed as compared to the burst phase of AL-NPC insertion (Figures S3E and S3F). We conclude that AL-NPCs are pore scaffolds devoid of most FG-Nups and all nuclear basket components. With the exception of Nup358, Nups that asymmetrically distribute across the NE-plane in NE-NPCs are absent from AL-NPCs (Figures 2E and 2E′).

Topology of AL Insertion

To address how AL-NPC insertion is topologically possible, we sought to identify putative steps of AL insertion by ultrastructural analysis. We first analyzed sections through staged blastoderm embryos by transmission electron microscopy after high-pressure freezing and freeze substitution. AL were apparent in the cytoplasm throughout the embryo as interconnected stacks of membranes containing NPCs (Figures S5A, S6A, and S6B–S6B″). AL that were close to the NE and thus potentially could engage in an insertion event, often appeared continuous with the NE (Figures 3A, 3B, S5B″, and S5C′). Strikingly, as evident in multiple sections, these AL-NE fusion sites often were adjacent to apparent openings within the NE (Figures 3A and 3B). At the edges of these openings, the INM and ONM were seen to be fused in the electron micrographs, emphasizing that the gaps are not sample preparation artifacts (Figures 3A and 3B). We used correlative light and electron microscopy as described above and recorded an electron tomogram at a site where a fluorescent spot of RFP::Nup107 close to the NE indicated a potentially ongoing insertion event (Figures 3C, 3C′, and S5B–S5B″). Although the observed membrane topology in this region was complex, it clearly showed the critical features: a patch of AL engaged with the NE in direct proximity to NE openings. In conclusion, the unanticipated discovery of NE openings offers a topological explanation for how the INM becomes continuous with AL (see below).

A limitation of the aforementioned analysis is that it resolves membranes only when they are roughly aligned parallel to the electron optical axis and thus manifest as a projection in the electron micrographs. Because AL assume various orientations with respect to the NE it is relatively unlikely to capture both in a favorable two-dimensional projection. To better resolve inserting AL sheets in 3D, we used the slice and view technique, in which a low angle focused ion beam is used to mill away thin (5–10 nm) layers of an embedded specimen alternating with image acquisition by focused ion beam-scanning electron microscopy (FIB-SEM). The resulting volumes have an almost isotropic resolution and can be virtually rotated to obtain slices in basically any spatial direction. We first confirmed that the parallel AL-NPC decorated ER sheets are indeed highly interconnected in three dimensions and link to the NE close to openings of the nuclear membrane (Figure S5C″). NE-openings were frequent and surprisingly large (Figures 3D and 3E). By tangentially slicing the NE in volumes obtained by FIB-SEM, we could resolve inserting AL-sheets as part of an ER compartment that enclosed large parts of the respective NE-opening and contained NPCs (Figures 3F–3F″ and 3G). We conclude that AL insertion typically occurs in proximity to NE openings.

The three-dimensional data allowed us to deduce putative topological intermediates of AL-insertion. Those involve establishing membrane connections from the adjacent sheet to the nuclear membranes and in proximity to openings of the existing NE. NE-openings could either form de novo or persist from the previous mitosis (Figures 4A and 4A′). Importantly their existence suggests a model for AL uptake that elegantly resolves the topological puzzle: NE openings link the INM to the inserting sheet and convert the latter into NE (Figures 3F, 3G, and 4A). Driven by nuclear expansion, both the adopted "novel" and the underlying "previously present" NE sheet laterally slide away from each other and augment nuclear surface (Figures 4A and 4B′). These topological intermediates can be viewed as part of a spatiotemporal continuum of AL and NE membranes. This model would predict that redundant pieces of NE should result from AL insertion (Figures 4A and 4B′). We indeed could confirm the existence of NPC-decorated, redundant NE membranes in both micrographs (Figures S5D–S5F) as well as nucleoplasmic GFP::Nup107 foci in live microscopy (Figure 6C). Such redundant NE could be resolved either by fission or re-insertion in an equivalent way as AL insertion from the cytoplasm.

The Permeability Barrier of the NE Is Maintained during AL Insertion

The apparent NE openings suggest a compromised permeability barrier between the nucleoplasm and the cytosol, except

Figure 3. Topology of AL Insertion

(A–B) NE-openings accompany AL insertions. (A and A′) Transmission electron micrographs (TEM) of two consecutive serial sections of a nucleus with closely aligned AL-NPCs (arrowheads). Note the clear INM-ONM connection of the encircled opening (A), which is replaced by intact NE in (A′), while the boxed opening is seen in both sections. (B) Electron micrograph of a nucleus where the NE is opened (boxed) and continuous with an ER stretch.

(C) Capture of an AL-insertion into the NE by correlative light and electron microscopy (CLEM) and tomography (C′). (C) RFP::Nup107 localizes to the NE and is enriched at proximal AL-NPCs (arrowhead).

(C′) Single slice through a tomogram recorded at the region depicted by the yellow box in (C). Arrowheads point to a membrane connection of the inserting AL to the NE, adjacent to a NE-opening.

(D) Multiple NE openings (arrowheads) are apparent in a volume containing a nucleus that was obtained by focused ion beam-scanning electron microscopy (FIB-SEM; a single slice is shown superimposed with the NE that is isosurface-rendered in blue).

(E) Histogram of NE-opening diameters measured in TEMs as represented in (A, A′, and B). Most NE-openings are in the range of 400–800 nm.

(F–G) FIB-SEM visualizes the continuity of AL membrane sheets with the NE. Slices through a FIB-SEM volume tangential to the nucleus (F–F″) and isosurface rendering of the same region (G). (F–F″) Slices at slightly different angles. Note that cross-sectioned NE with NPCs in side view (yellow arrowheads in F–F″) are perpendicular to an AL membrane sheet that has a branched topology and contains multiple NPC in top view (white arrowheads in F–F″).

See also Figures S5 and S6.

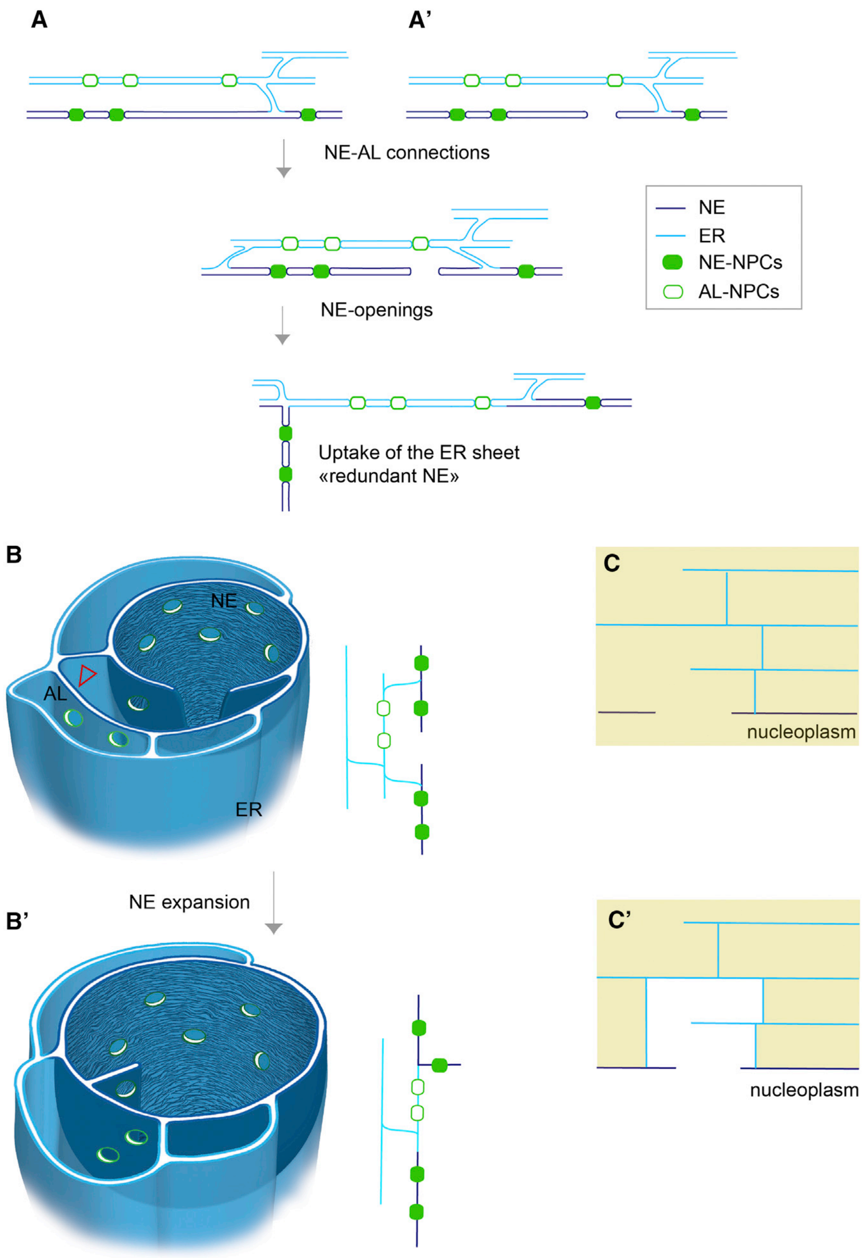

Figure 4. Model of AL-NPC Insertion

(A–B′) Planar (A and A′) or three-dimensional (B and B′) model for AL-NPC insertion, as inferred from electron micrographs. Insertion involves the establishment of membrane connections from the inserting ER sheet containing AL-NPCs to the NE and opening of the NE adjacent to the connected ER sheet. NE openings could emerge de novo by an unknown mechanism (A) or alternatively could remain from the previous round of mitosis (A′). The connected ER sheet (arrowhead in B) becomes part of the NE while nuclear surface increases by lateral dissipation within linked membrane planes. Because pores limit lateral membrane dissipation, NE-NPCs located in between the NE opening and the AL-sheet connection predict the formation of transient redundant NE stretches (A and B′).

(C and C′) The permeability barrier of the NE would be compromised if inserting ER sheets fail to entirely surround (or sufficiently "shield") the NE opening against the cytoplasm (C). Alternatively no cytoplasmic influx through the NE opening would occur in a concealed compartment (C′).

that the NE openings would reside in a compartment that is entirely surrounded by ER-membranes (Figures 4C and 4C′). The electron microscopy data (Figures 3C′ and 3G) highlight the topological complexity of AL insertion and indicate that the same event might span a considerable fraction of the nuclear surface area. As such, it is not ultimately possible to conclude from the three-dimensional data whether or not the NE remains topologically closed during AL insertion. We therefore set out to experimentally test if the NE permeability can be maintained despite AL fusion. We imaged GFP::Nup107-expressing blastoderm embryos that were injected with fluorescently labeled dextrans of different molecular weight (Lénárt and Ellenberg, 2006). Small dextrans of 10 and 25 kDa were not excluded by NPCs and diffused into the nucleoplasm during interphase (Figures 5A and 5A′). In contrast, nuclei were impermeable to 155 kDa dextran (Figure 5A″), suggesting an intact barrier. Importantly, 155 kDa dextran did not even leak into the nucleoplasm as AL inserted to the NE (Figures 5B–5B″ and 5E; Movie S5), demonstrating that insertion of AL does not interfere with the permeability barrier of the nuclear membranes.

To test if an NE opening of the observed size would in principle cause dextran influx, we artificially ruptured the nuclear membranes by performing laser nanosurgery on the NE. Using GFP::Nup107, we targeted the NE with a 950 nm Titan Sapphire Laser and punctured the nuclear membranes (Figures 5C–5C″; Movie S6). Successful puncture was reflected by a strong mechanical response of the entire nucleus apparent as NE folding and tumbling (Movie S6). Strikingly, 155 kDa-dextran accumulated in the nucleoplasm of punctured nuclei within tens of seconds (Figures 5C′ and 5C″; Movie S6) with kinetics that did not depend on the size of the punctured region (Figure 5C″). These experiments conclusively demonstrate that the permeability barrier of the NE was disrupted after laser-induced rupture while it was not impaired when AL inserted (Figure 5E), despite comparable dimensions of the respective NE openings apparent in electron micrographs (Figure 3E). These findings are in line with our topological model and suggest that the NE-openings are entirely surrounded by ER membrane sheets.

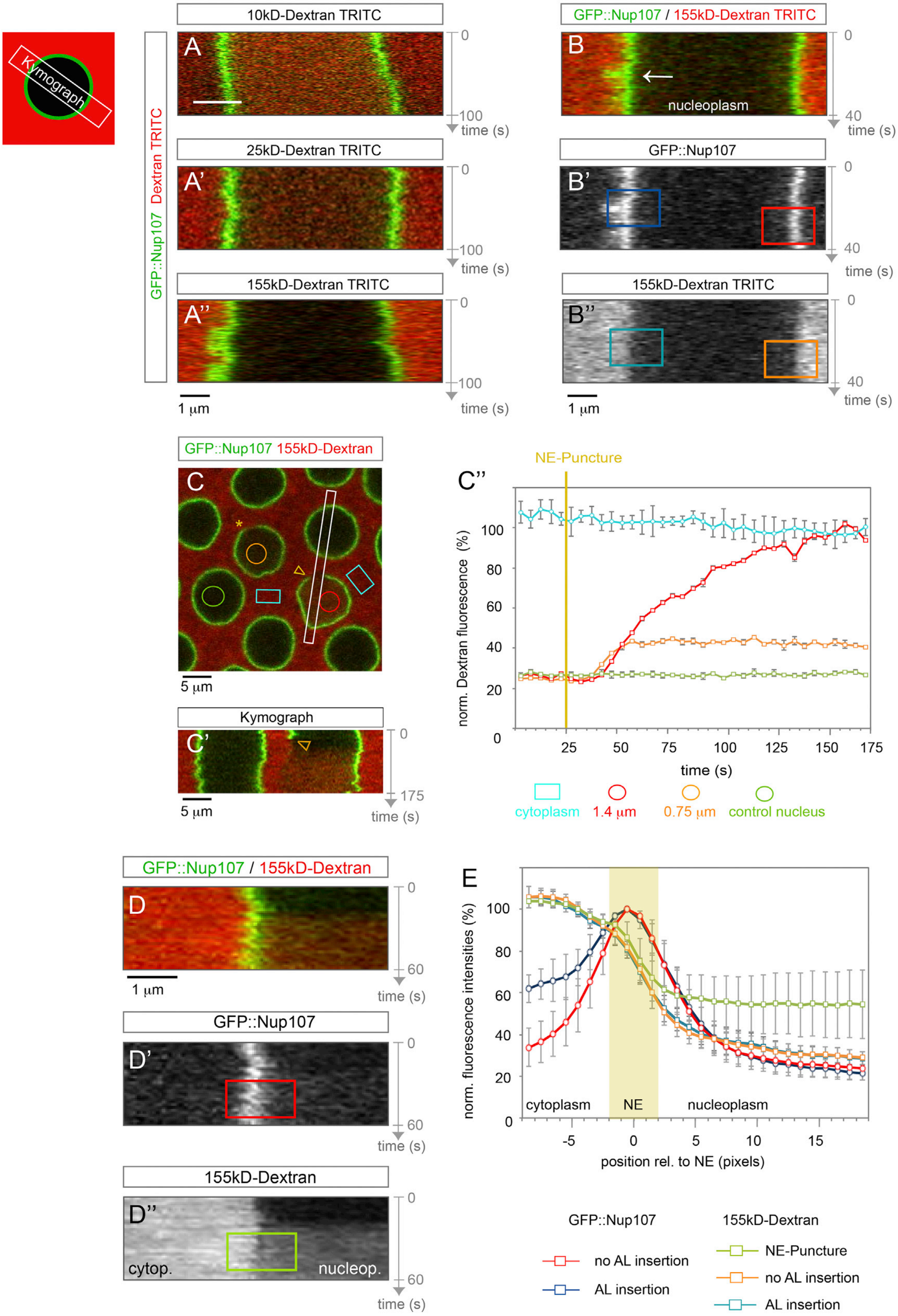

(legend on next page)

NPC Organization and Insertion Mode Change during Development

In mammalian cell lines, NPCs are stationary embedded within the NE but mobile along ER membranes in AL (Daigle et al., 2001). Our results demonstrate frequent AL insertions in blastoderm embryos and indicate that AL-NPCs predominantly contribute to an increased NE-NPC number during nuclear expansion. Lateral mobility of NPCs within the NE could facilitate their re-distribution following AL insertion. We thus performed FRAP experiments on GFP::Nup107 expressing embryos to test whether AL-NPC became immobile upon NE insertion. Strikingly, we observed fast recovery of GFP::Nup107 all along the rim after photobleaching (Figures 6A and 6A′). Together with our finding that NPC material laterally dispersed after AL insertion to the NE (Figures 1F–1F″), we conclude that in the *Drosophila* syncytial blastoderm NPCs are mobile within the NE. In contrast, GFP::Nup107 fluorescence did not recover after photo-bleaching of nuclei at the onset of gastrulation, suggesting that dispersion of pores within the envelope was abolished (Figures 6B and 6B′). The impaired NE-NPC mobility in those nuclei was reflected by distinct principles of pore organization along the NE when compared to nuclei in younger embryos. GFP::Nup107 distributed uniformly along the NE of blastoderm embryos but appeared clustered into distinct steady foci just before embryos started to gastrulate (Figures 6C and 6D). The switch in NPC mobility and organization coincides with the activation of the zygotic genome (Figure 7A). Thus pore organization at the NE could be controlled by zygotic genes or by transcription-associated changes in chromatin. Consistent with both hypotheses, injection of the RNA polymerase inhibitor α-amanitin prevented clustering of NPCs on later stage nuclei (Figure 6E).

The nuclear lamina, a meshwork of intermediate filament proteins that underlies the NE and projects into the nucleoplasm in metazoa is critical for NPC organization and mobility within the NE (Daigle et al., 2001), but other NE proteins could also be important for NPC mobility. Comparative analysis of the proteomes of isolated nuclei from either blastoderm or gastrulating embryos revealed a significant ($p = 0.00024$) ~2.5-fold enrichment of a very prominent INM protein, lamin B receptor (LBR), in older nuclei (data not shown). In mammalian cells LBR is recruited by the Y-complex member ELYS/Mel28 to specific NE-subdomains and could thus directly link to NPC distribution (Clever et al., 2012). Strikingly, LBR was absent from the NE in syncytial embryos but became localized to the rim of somatic nuclei during cellularization in interphase 14 (Figures S7A and S7B). Notably, the protein remained undetectable at the NE of nuclei from pole cells, which are the posteriorly localized germ cell progenitors (Figures S7C and S7D). To address whether LBR is sufficient to alter NPC organization at the NE, we ectopically expressed the protein in WGA-injected syncytial blastoderm embryos. In these embryos pores appeared clustered within the NE and nuclei acquired an irregular morphology (Figure 6F). Both phenotypes are reminiscent of wild-type nuclei at gastrulation onset. Strikingly, ectopic expression of LBR in the early embryo also induced larger AL sizes (Figures 6F′and 6G), implying that LBR expression and NPC clustering counteracts AL-insertion.

At last, we detected striking differences in the NPC insertion mode between the different developmental stages. In contrast to the early embryo (compare to Figure 1C), the mean fluorescent intensities of either GFP::Nup107 or fluorescently labeled WGA at the nuclear rim strongly decreased as interphase 14 proceeded (Figure 6H), while nuclei significantly increase their surface area (Fullilove and Jacobson, 1971). Interestingly, the switch in NPC organization and insertion is concomitant with the reported (Onischenko et al., 2004; Stafstrom and Staehelin, 1984a) and observed (Figures 6D and 6I) disappearance of AL-NPCs from the cortical nuclei layer at early gastrulation. At the same time AL remained abundant in pole cells (Figure 6I), suggesting that nuclei from the prospective soma and germline have different NE organizations, compatible with our results on differential LBR localization (Figure S7). Jointly, our data suggest that AL insertion to the NE is an "early developmental" program that is reduced or lost as the embryo matures (Figure 7).

Figure 5. The Permeability Barrier of the NE Is Maintained during AL Insertion

(A) Kymographs from time-lapse movies recorded in syncytial blastoderm embryos expressing GFP::Nup107 injected with fluorescently labeled dextrans of different molecular weight. Regions of interest (ROIs) for kymographs span entire nuclei as schematically depicted. The NE is permeable for 10 kDa (A) and 25 kDa dextran (A′), but not for 155 kDa dextran (A″).

(B–B″) The NE stays impermeable upon AL-insertion. Kymographs (scheme) of a time-lapse movie imaging an embryo expressing GFP::Nup107 (B and B′) injected with dextran-155 kDa-TRITC (B and B″). Dextran stays cytoplasmic upon insertion of GFP::Nup107-labeled AL-NPCs (arrow). Colored ROIs refer to the graph in (E).

(C–E) Laser puncture of the NE compromises its permeability barrier. (C) Top view still of a time-lapse movie imaging a GFP::Nup107 expressing syncytial embryo injected with dextran-155 kDa-TRITC, where the NE of two nuclei was laser punctured simultaneously (indicated by the arrowhead and asterisk, respectively). (C′) Kymograph of the respective movie along the ROI (white box in C). Dextran leaks into the nucleoplasm of the punctured nucleus (arrowheads in C and C′), but not in the neighboring control nucleus. (C″) Quantification of the mean dextran-TRITC fluorescence intensities ± SD for the respective ROIs color-coded in (C). Dextran accumulates in the nucleoplasm of nuclei within ~20 s after puncture with an initial kinetics that is independent of the size of the punctured region. (D) Representative kymograph for GFP::Nup107 (D′) and dextran-155 kDa-TRITC (D″) after NE-puncture; colored ROIs refer to the graph in (E). (E) Dextran leaks into the nucleoplasm upon NE puncture, but not when AL insert to the NE. Quantitation of dextran influx upon NE-puncture and AL insertion. GFP::Nup107 and 155 kDa-dextran-TRITC levels were inferred from their respective fluorescence intensities, determined from line scans on ROIs in kymographs as exemplified in (B′), (B″), (D′), and (D″). Mean fluorescence intensities ± SD are plotted as a function of the distance from the NE for control nuclei (no insertion, n = 10 nuclei), nuclei with AL-NPC insertions (n = 7) or punctured nuclei (n = 7). Experiments were aligned by the respective maximal GFP::Nup107 intensity value, delineating the position of the NE.

See also Movies S5 and S6.

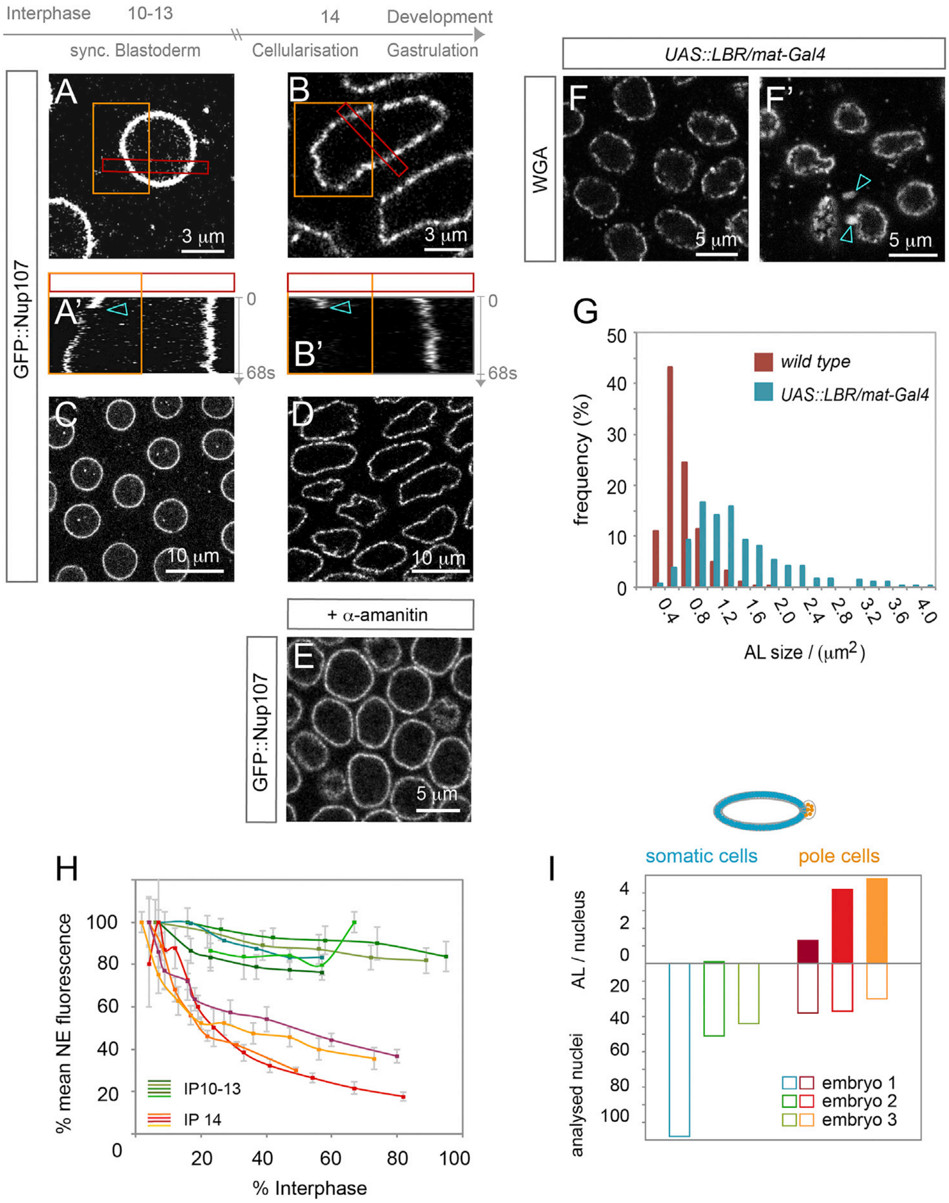

Figure 6. Developmental Regulation of NPC Organization and Insertion

(A–D) Lateral mobility and organization of NPCs change during development. Representative top view stills (A–D) and kymographs (A′ and B′) of time-lapse movies imaging GFP::Nup107 in syncytial embryos (A, A′, and C) or before gastrulation onset in interphase 14 (B, B′, and D). GFP::Nup107 at the NE was photobleached (arrowheads in A′ and B′) at the depicted orange regions of interest (ROIs) (A and B) and recovered at syncytial blastoderm nuclei (A′) but not at nuclei from interphase 14 embryos (B′). For both kymographs the respective ROIs are boxed in red in (A) and (B). (C and D) GFP::Nup107 distributes evenly along the NE at the spherical nuclei of syncytial embryos (C) but appears clustered at the rim of the irregularly shaped nuclei at gastrulation onset (D).

(E) Pore clustering is zygotically induced. Top view still from a movie recording a GFP::Nup107 expressing embryo in interphase 14, injected with α-amanitin, where nuclei stay round and pores fail to cluster due to inhibition of zygotic gene activation.

(F–G) The zygotically induced gene LBR is sufficient to cluster NPCs and increase AL size. (F and F′) Top view stills from two syncytial blastoderm embryos where LBR expression was maternally induced. WGA labeled NPCs appeared clustered along the NE (F), similar to wild-type embryos in interphase 14 (B and D) and accumulate in larger AL-NPCs (F′) (arrowheads). (G) Histogram of AL-NPC sizes measured from images as shown in (C and F′). AL-NPCs are larger in blastoderm embryos where LBR expression was ectopically induced (n = 282 AL), compared to control embryos (n = 282).

(H) The mode of NPC insertion is developmentally controlled. NPC density along the NE was inferred from the mean fluorescence intensities of GFP::Nup107 or fluorescently labeled WGA, which were normalized and plotted as a function of interphase progression. NPC densities stayed almost constant in syncytial interphases but strongly decayed in interphase 14 (see also Figure 1C). Plotted are mean fluorescence intensities ± SD for ~10 nuclei from each imaged syncytial blastoderm (n = 5) or cellularizing (n = 4) embryo.

(I) During interphase 14, AL-NPCs were diminished from the somatic nuclear layer at the embryo's cortex but not from pole cells.

See also Figure S7.

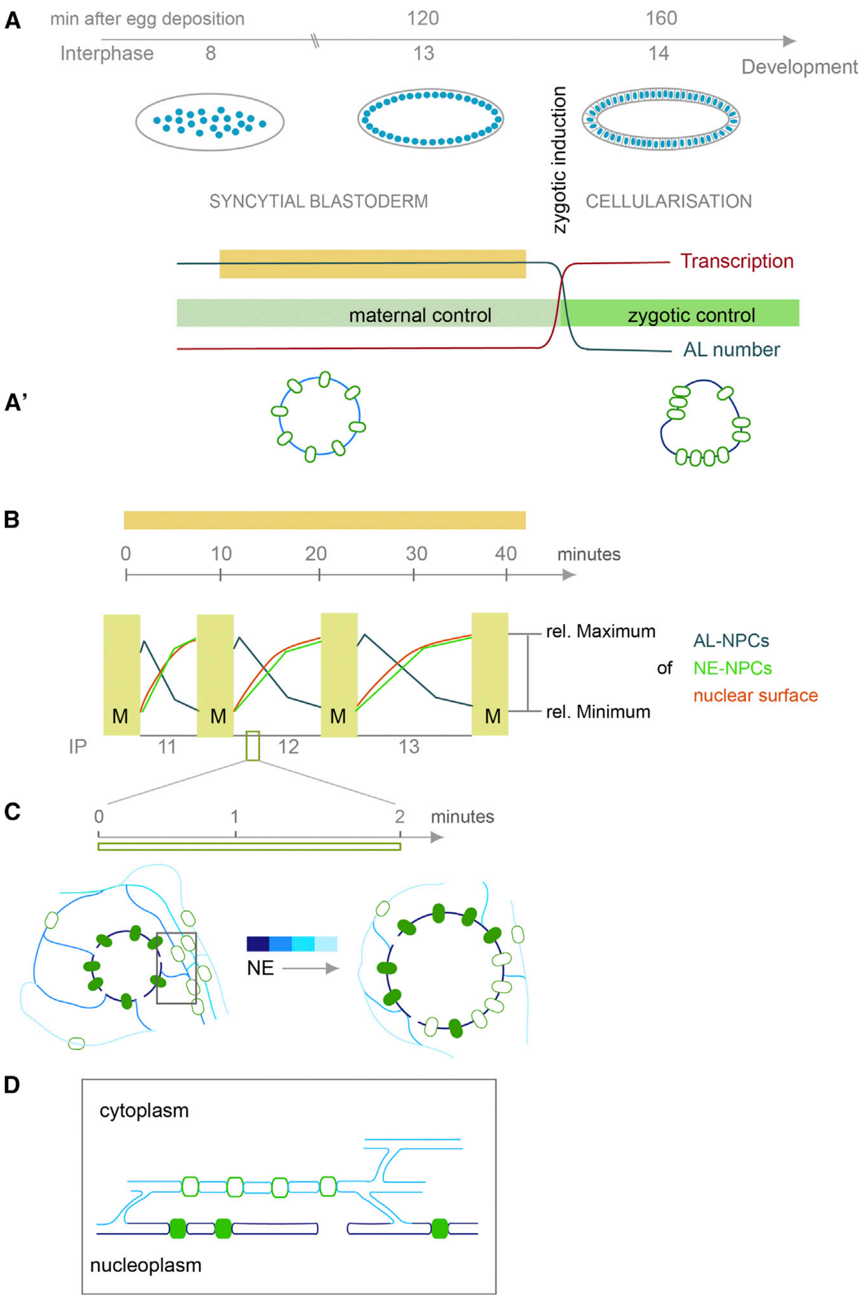

Figure 7. NPC Insertion in the Context of Embryonic Development

(A) AL are abundant throughout the ~120 min of syncytial development but diminish from the cortical layer in interphase 14, concomitant with cellularization and the transcriptional activation at zygotic induction.

(A′) NE-NPCs are laterally mobile and all along the NE in the syncytial blastoderm, but immobilize and cluster at the NE starting with cellularization.

(B) In each precedent interphase of the syncytial blastoderm, AL number oscillates within the cortical nuclear layer on a timescale of ~10 min, with increasingly longer interphases in each cycle. Inverse to AL-NPC number, which decreases in each interphase, NE-NPC number increases together with nuclear surface expansion.

(C and D) AL-NPCs insertion to the NE occurs in the range of 1–2 min. It involves an open NE and batch insertion of AL-NPCs within a proximal ER sheet (D). Lateral mobility allows NE-redistribution of inserted NPCs. Insertion of subsequent ER sheets augments nuclear surface (C).

DISCUSSION

Collectively, the following scenario emerges from our data. AL are abundant in early *Drosophila* embryos and predominantly contribute to maintain the constant NE-NPC density in the expanding NE during interphase. The abundance of AL at the cortical nuclei layer thereby oscillates together with the progression of the consecutive interphases until the start of global transcription when AL disappear and the mode of NPC insertion changes (Figures 7A and 7B). During each onset of early interphases, AL-NPCs are assembled similarly to NE-NPCs but since the combined nuclear surface of the two daughter nuclei is smaller as compared to the parental nucleus, they remain in the cytoplasm. As interphases progress, AL-NPCs feed into the pool of NE-NPCs alongside ER membranes that augment NE surface during rapid nuclear expansion (Figure 7C). AL insertion is enabled by NE openings that might either persist from previous mitosis or form de novo by an unknown mechanism (Figure 7D). Upon AL insertion, the NE permeability barrier remains unperturbed, likely because the NE openings are entirely surrounded by the ER network. The inserting NPCs comprise pre-assembled NPC scaffolds that recruit the full set of Nups only subsequent to insertion and only then establish transport competence.

Why do the expanding nuclei of the syncytial blastoderm maintain a constant number of NPCs per surface area despite their transcriptional inactivity? One might surmise that this is due to mechanical properties but also temporal constraints. The insertion of NPCs might be crucial to enable the massive influx of material into the nucleoplasm during nuclear expansion (volume increase). Indeed, the strained configuration of nuclei is reflected by their strong mechanical response (NE tumbling) upon disruption of the NE and permeability barrier after laser puncture. Second, the batch transfer of entire NPC scaffolds as inherent parts of membrane sheets overcomes the described kinetic constrains of interphase assembly in mammalian cells, that are not compatible with the short interphases in the *Drosophila* syncytium (Dultz and Ellenberg, 2010). Given the abundance of AL-NPCs and the reported high insertion rate of NPCs into the NE of *Xenopus*

leavis oocytes (D'Angelo et al., 2006) it appears likely that similar mechanisms operate in vertebrates. It remains unclear how sufficient amounts of AL are generated to globally feed nuclear surface expansion over multiple cell cycles until the start of transcription. However, Nups are maternally provided and AL are abundant not only at the cortical layer of nuclei but also within the interior of the embryo (Figures S6A and S6B–S6B″). Therefore, a possibility that needs to be considered is that a source of AL-NPCs already generated during oogenesis feeds nuclear growth throughout the syncytial blastoderm.

In addition to their eminent role in transport, NE-NPCs organize the nuclear periphery by delineating zones of active euchromatin as compared to transcriptionally repressed heterochromatin in between pores (Ptak et al., 2014). Crucial to this is that NPCs are laterally immobile within the NE, which was shown to depend on the nuclear lamina (Daigle et al., 2001). Lamins are nuclear intermediate filament proteins and come in two major types: B-type Lamins are ubiquitous, while A-type Lamins are expressed exclusively when cells differentiate. Both proteins engage in distinct meshworks and also impact on NPC insertion rate (Lenz-Böhme et al., 1997; Liu et al., 2000). Our work puts NPC organization and the mode of pore insertion into a developmental context. We propose that in *Drosophila* AL insertion is innate to earliest embryogenesis and diminishes when pores get laterally restricted and cluster at the NE. There are no A-type lamins expressed at that stage, and specifically expressed INM proteins could be crucial. Intriguingly, the formation of immobile pore clusters coincides with the transcriptional upregulation of hundreds of genes at zygotic induction, a developmental transition present in all metazoan that is accompanied by characteristic changes in chromatin signatures (Rudolph et al., 2007; Vastenhouw et al., 2010). We reveal that the zygotically upregulated INM protein LBR, a developmentally controlled INM tether of peripheral heterochromatin (Solovei et al., 2013), is sufficient to prematurely aggregate NPCs in blastoderm interphases, when artificially expressed earlier in embryogenesis. This also leads to larger AL likely because LBR counteracts AL insertion for which lateral NPC mobility is required. Our data suggest a zygotically induced regulation that links pore insertion and organization, NE composition and ultimately also chromatin organization at the nuclear periphery. All of these events eventually contribute to the commitment of originally pluripotent somatic nuclei into distinct lineages.

EXPERIMENTAL PROCEDURES

Detailed experimental procedures are available in the Supplemental Information.

Embryo Injections, Live Imaging, and Immunostainings

Staged embryos were treated according to standard protocols and injected with Alexa488 or Alexa555 conjugated WGA (100 μg/ml, Life Technologies), α-amanitin (100 μg/ml, Sigma) or TRITC/FITC conjugated dextrans of different molecular weight (Lénárt and Ellenberg, 2006). Subsequently embryos were imaged on an inverted Zeiss LSM780 confocal microscope equipped with a 63×/1,4 NA oil immersion objective. For immunostainings, embryos were fixed in 4% formaldehyde and processed according to standard protocols.

Sub-cellular Fractionation and Protein Identification by Mass Spectrometry

Dechorionated embryos were lysed and nuclei were isolated by centrifugation at 5,000 rpm for 13 min and stripped from attached membranes by centrifugation (45 min, 12,000 rpm) through a 1 M sucrose cushion. Microsomal membranes were isolated by spinning the supernatant from the nuclear precipitate at 40,000 rpm for 45 min. Samples were further processed and analyzed by shotgun mass spectrometry as previously described (Mackmull et al., 2015). Raw files for quantitative label free analysis were analyzed using MaxQuant (Cox and Mann, 2008) and the MS/MS spectra were searched against the *Drosophila* Swiss-Prot entries using the Andromeda search engine (Cox et al., 2011). Protein differential expression was evaluated using the Limma package. Differences in protein abundances were statistically determined using the Student's t test moderated by the empirical Bayes method. Significant regulated proteins were defined by a cut-off of log2 fold change ≤ -1 or ≥ 1 and p value $\leq$ 0.01.

Transmission Electron Micrograph, FIB-SEM, and Correlative Light and Electron Microscopy Imaging

Embryos were high pressure frozen, freeze-substituted and infiltrated with resin. Blocks were subsequently trimmed for FIB-SEM or cut into 300-nm sections for correlative light and electron microscopy (CLEM) analysis using an ultramicrotome. For serial transmission electron microscopy (TEM), the resin-embedded embryos were trimmed and consecutive 100 nm distant sections were obtained with a section thickness of ~80 nm. TEM imaging was carried out on a FEI Tecnai F30 equipped with Gatan US4000 CCD camera, operated at 300 kV or a FEI Biotwin equipped with an Olympus Keen View G2 camera operated at 120 kV, respectively. The fluorescence microscopy (FM) imaging was carried out as previously described (Avinoam et al., 2015; Kukulski et al., 2011). Tomography was performed in 1° increments at 4,700× magnification on a FEI Tecnai F30 electron microscope. FIB-SEM imaging was carried out on an Auriga 60 (Zeiss) using the Atlas3D software. Datasets were acquired with 5 nm pixel size and 5 nm steps in z and aligned using TrakEM (Fiji).

ACCESSION NUMBERS

The accession number for the mass spectrometry proteomics data reported in this paper is ProteomeXchange Consortium: PXD004120.

SUPPLEMENTAL INFORMATION

Supplemental Information includes Supplemental Experimental Procedures, seven figures, and six movies and can be found with this article online at http://dx.doi.org/10.1016/j.cell.2016.06.015.

AUTHOR CONTRIBUTIONS

B.H. conceived the project, designed and performed experiments, analyzed data, and wrote the manuscript. M.T.M. designed and performed experiments and analyzed data. P.M., P.R., K.H.B., N.S., R.S.M., A.A.P., A.N., and J.M.P. performed experiments. T.L. designed experiments. Y.S. designed experiments and oversaw the project. M.B. conceived the project, designed experiments, analyzed data, oversaw the project, and wrote the manuscript.

ACKNOWLEDGMENTS

We thank Drs. Iain Mattaj and Peter Lenart for critical reading of the manuscript and Dr. Yannick Azou-Gros for experimental assistance. We are grateful to our colleagues Drs. V. Doye, N. Dostatni, A. Ephrussi, S. deRenzis, M. Mavrakis, A. Akhtar, G. Krohne, I. Mattaj, and J. Ellenberg for reagents. Stocks obtained from the Bloomington *Drosophila* Stock Center (NIH P40OD018537) were used in this study. We gratefully acknowledge support by the European Molecular Biology Laboratory (EMBL) advanced light microscopy (ALMF), electron

microscopy (EMCF), and proteomic (PCF) core facilities and Petra Riedinger for graphical design. We are grateful to Drs. P. Paul-Gilloteaux, X. Heiligenstein, and S. Mosalaganti for assistance and expertise in correlative light and electron microscopy analysis. B.H. was supported by the Agence Nationale de la Recherche (ANR) (Programme Blanc) NeMo (to T.L) and the European Molecular Biology Organisation (EMBO). K.H.B. was supported by postdoctoral fellowships from the Swiss National Science Foundation (SNF), EMBO, and Marie Curie Actions. M.B. acknowledges funding by EMBL and the European Research Council (309271-NPCAtlas).

Received: October 25, 2015
Revised: April 15, 2016
Accepted: June 3, 2016
Published: July 7, 2016

REFERENCES

Avinoam, O., Schorb, M., Beese, C.J., Briggs, J.A., and Kaksonen, M. (2015). ENDOCYTOSIS. Endocytic sites mature by continuous bending and remodeling of the clathrin coat. Science *348*, 1369–1372.

Clever, M., Funakoshi, T., Mimura, Y., Takagi, M., and Imamoto, N. (2012). The nucleoporin ELYS/Mel28 regulates nuclear envelope subdomain formation in HeLa cells. Nucleus *3*, 187–199.

Cordes, V.C., Reidenbach, S., and Franke, W.W. (1996). Cytoplasmic annulate lamellae in cultured cells: composition, distribution, and mitotic behavior. Cell Tissue Res. *284*, 177–191.

Cox, J., and Mann, M. (2008). MaxQuant enables high peptide identification rates, individualized p.p.b.-range mass accuracies and proteome-wide protein quantification. Nat. Biotechnol. *26*, 1367–1372.

Cox, J., Neuhauser, N., Michalski, A., Scheltema, R.A., Olsen, J.V., and Mann, M. (2011). Andromeda: a peptide search engine integrated into the MaxQuant environment. J. Proteome Res. *10*, 1794–1805.

D'Angelo, M.A., Anderson, D.J., Richard, E., and Hetzer, M.W. (2006). Nuclear pores form de novo from both sides of the nuclear envelope. Science *312*, 440–443.

Daigle, N., Beaudouin, J., Hartnell, L., Imreh, G., Hallberg, E., Lippincott-Schwartz, J., and Ellenberg, J. (2001). Nuclear pore complexes form immobile networks and have a very low turnover in live mammalian cells. J. Cell Biol. *154*, 71–84.

Doucet, C.M., and Hetzer, M.W. (2010). Nuclear pore biogenesis into an intact nuclear envelope. Chromosoma *119*, 469–477.

Doucet, C.M., Talamas, J.A., and Hetzer, M.W. (2010). Cell cycle-dependent differences in nuclear pore complex assembly in metazoa. Cell *141*, 1030–1041.

Dultz, E., and Ellenberg, J. (2010). Live imaging of single nuclear pores reveals unique assembly kinetics and mechanism in interphase. J. Cell Biol. *191*, 15–22.

Dultz, E., Zanin, E., Wurzenberger, C., Braun, M., Rabut, G., Sironi, L., and Ellenberg, J. (2008). Systematic kinetic analysis of mitotic dis- and reassembly of the nuclear pore in living cells. J. Cell Biol. *180*, 857–865.

Fath, S., Mancias, J.D., Bi, X., and Goldberg, J. (2007). Structure and organization of coat proteins in the COPII cage. Cell *129*, 1325–1336.

Fischer, J., Teimer, R., Amlacher, S., Kunze, R., and Hurt, E. (2015). Linker Nups connect the nuclear pore complex inner ring with the outer ring and transport channel. Nat. Struct. Mol. Biol. *22*, 774–781.

Fullilove, S.L., and Jacobson, A.G. (1971). Nuclear elongation and cytokinesis in Drosophila montana. Dev. Biol. *26*, 560–577.

Grossman, E., Medalia, O., and Zwerger, M. (2012). Functional architecture of the nuclear pore complex. Annu. Rev. Biophys. *41*, 557–584.

Hülsmann, B.B., Labokha, A.A., and Görlich, D. (2012). The permeability of reconstituted nuclear pores provides direct evidence for the selective phase model. Cell *150*, 738–751.

Katsani, K.R., Karess, R.E., Dostatni, N., and Doye, V. (2008). In vivo dynamics of Drosophila nuclear envelope components. Mol. Biol. Cell *19*, 3652–3666.

Kessel, R.G. (1983). The structure and function of annulate lamellae: porous cytoplasmic and intranuclear membranes. Int. Rev. Cytol. *82*, 181–303.

Kukulski, W., Schorb, M., Welsch, S., Picco, A., Kaksonen, M., and Briggs, J.A. (2011). Correlated fluorescence and 3D electron microscopy with high sensitivity and spatial precision. J. Cell Biol. *192*, 111–119.

Laurell, E., Beck, K., Krupina, K., Theerthagiri, G., Bodenmiller, B., Horvath, P., Aebersold, R., Antonin, W., and Kutay, U. (2011). Phosphorylation of Nup98 by multiple kinases is crucial for NPC disassembly during mitotic entry. Cell *144*, 539–550.

Lénárt, P., and Ellenberg, J. (2003). Nuclear envelope dynamics in oocytes: from germinal vesicle breakdown to mitosis. Curr. Opin. Cell Biol. *15*, 88–95.

Lénárt, P., and Ellenberg, J. (2006). Monitoring the permeability of the nuclear envelope during the cell cycle. Methods *38*, 17–24.

Lenz-Böhme, B., Wismar, J., Fuchs, S., Reifegerste, R., Buchner, E., Betz, H., and Schmitt, B. (1997). Insertional mutation of the Drosophila nuclear lamin Dm0 gene results in defective nuclear envelopes, clustering of nuclear pore complexes, and accumulation of annulate lamellae. J. Cell Biol. *137*, 1001–1016.

Liu, J., Rolef Ben-Shahar, T., Riemer, D., Treinin, M., Spann, P., Weber, K., Fire, A., and Gruenbaum, Y. (2000). Essential roles for Caenorhabditis elegans lamin gene in nuclear organization, cell cycle progression, and spatial organization of nuclear pore complexes. Mol. Biol. Cell *11*, 3937–3947.

Longo, F.J., and Anderson, E. (1968). The fine structure of pronuclear development and fusion in the sea urchin, Arbacia punctulata. J. Cell Biol. *39*, 339–368.

Mackmull, M.T., Iskar, M., Parca, L., Singer, S., Bork, P., Ori, A., and Beck, M. (2015). Histone deacetylase inhibitors (HDACi) cause the selective depletion of bromodomain containing proteins (BCPs). Mol. Cell. Proteomics *14*, 1350–1360.

Newport, J., and Kirschner, M. (1982). A major developmental transition in early Xenopus embryos: II. Control of the onset of transcription. Cell *30*, 687–696.

Onischenko, E.A., Gubanova, N.V., Kieselbach, T., Kiseleva, E.V., and Hallberg, E. (2004). Annulate lamellae play only a minor role in the storage of excess nucleoporins in Drosophila embryos. Traffic *5*, 152–164.

Onischenko, E.A., Gubanova, N.V., Kiseleva, E.V., and Hallberg, E. (2005). Cdk1 and okadaic acid-sensitive phosphatases control assembly of nuclear pore complexes in Drosophila embryos. Mol. Biol. Cell *16*, 5152–5162.

Ptak, C., Aitchison, J.D., and Wozniak, R.W. (2014). The multifunctional nuclear pore complex: a platform for controlling gene expression. Curr. Opin. Cell Biol. *28*, 46–53.

Rudolph, T., Yonezawa, M., Lein, S., Heidrich, K., Kubicek, S., Schäfer, C., Phalke, S., Walther, M., Schmidt, A., Jenuwein, T., and Reuter, G. (2007). Heterochromatin formation in Drosophila is initiated through active removal of H3K4 methylation by the LSD1 homolog SU(VAR)3-3. Mol. Cell *26*, 103–115.

Schejter, E.D., and Wieschaus, E. (1993). Functional elements of the cytoskeleton in the early Drosophila embryo. Annu. Rev. Cell Biol. *9*, 67–99.

Solovei, I., Wang, A.S., Thanisch, K., Schmidt, C.S., Krebs, S., Zwerger, M., Cohen, T.V., Devys, D., Foisner, R., Peichl, L., et al. (2013). LBR and lamin A/C sequentially tether peripheral heterochromatin and inversely regulate differentiation. Cell *152*, 584–598.

Soupart, P., and Strong, P.A. (1974). Ultrastructural observations on human oocytes fertilized in vitro. Fertil. Steril. *25*, 11–44.

Spindler, M., and Hemleben, C. (1982). Formation and possible function of annulate lamellae in a planktic foraminifer. J. Ultrastruct. Res. *81*, 341–350.

Stafstrom, J.P., and Staehelin, L.A. (1984a). Are annulate lamellae in the Drosophila embryo the result of overproduction of nuclear pore components? J. Cell Biol. *98*, 699–708.

Stafstrom, J.P., and Staehelin, L.A. (1984b). Dynamics of the nuclear envelope and of nuclear pore complexes during mitosis in the Drosophila embryo. Eur. J. Cell Biol. *34*, 179–189.

Stuwe, T., Bley, C.J., Thierbach, K., Petrovic, S., Schilbach, S., Mayo, D.J., Perriches, T., Rundlet, E.J., Jeon, Y.E., Collins, L.N., et al. (2015). Architecture of the fungal nuclear pore inner ring complex. Science *350*, 56–64.

Vastenhouw, N.L., Zhang, Y., Woods, I.G., Imam, F., Regev, A., Liu, X.S., Rinn, J., and Schier, A.F. (2010). Chromatin signature of embryonic pluripotency is established during genome activation. Nature *464*, 922–926.

von Appen, A., Kosinski, J., Sparks, L., Ori, A., DiGuilio, A.L., Vollmer, B., Mackmull, M.T., Banterle, N., Parca, L., Kastritis, P., et al. (2015). In situ structural analysis of the human nuclear pore complex. Nature *526*, 140–143.

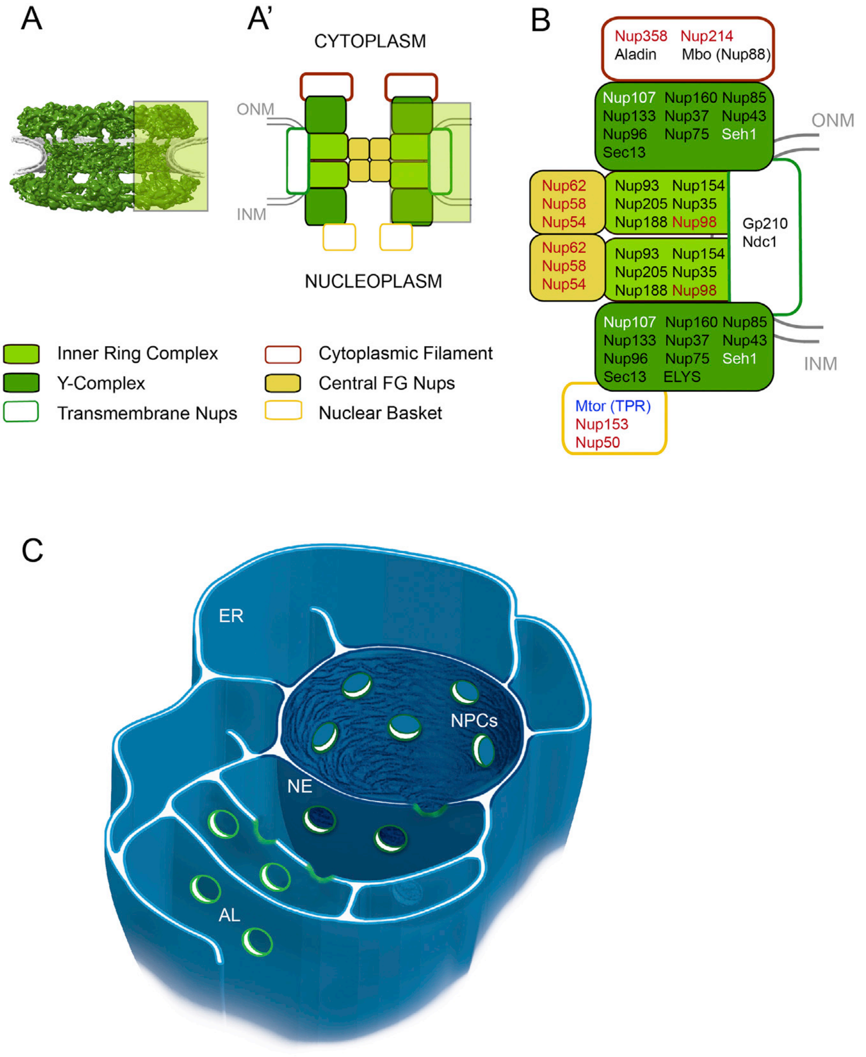

Figure S1. NPC Composition, Related to Figure 1

(A) Cryo-EM structure of the human NPC (von Appen et al., 2015) together with nuclear membranes (gray).

(A′) Schematic representation of NPC sub-complexes. Transmembrane Nups, inner ring- and Y-complex are symmetric while distinct sub-complexes create asymmetry across the nuclear envelope.

(B) Constituents of the *Drosophila* NPC. Nups that were used in imaging experiments in this study are highlighted in white (Y-complex Nups), red (FG-Nups) and blue (Mtor).

(C) Nuclear envelope (NE), endoplasmic reticulum (ER) and annulate lamellae (AL) constitute one membrane continuum and share a common lumen.

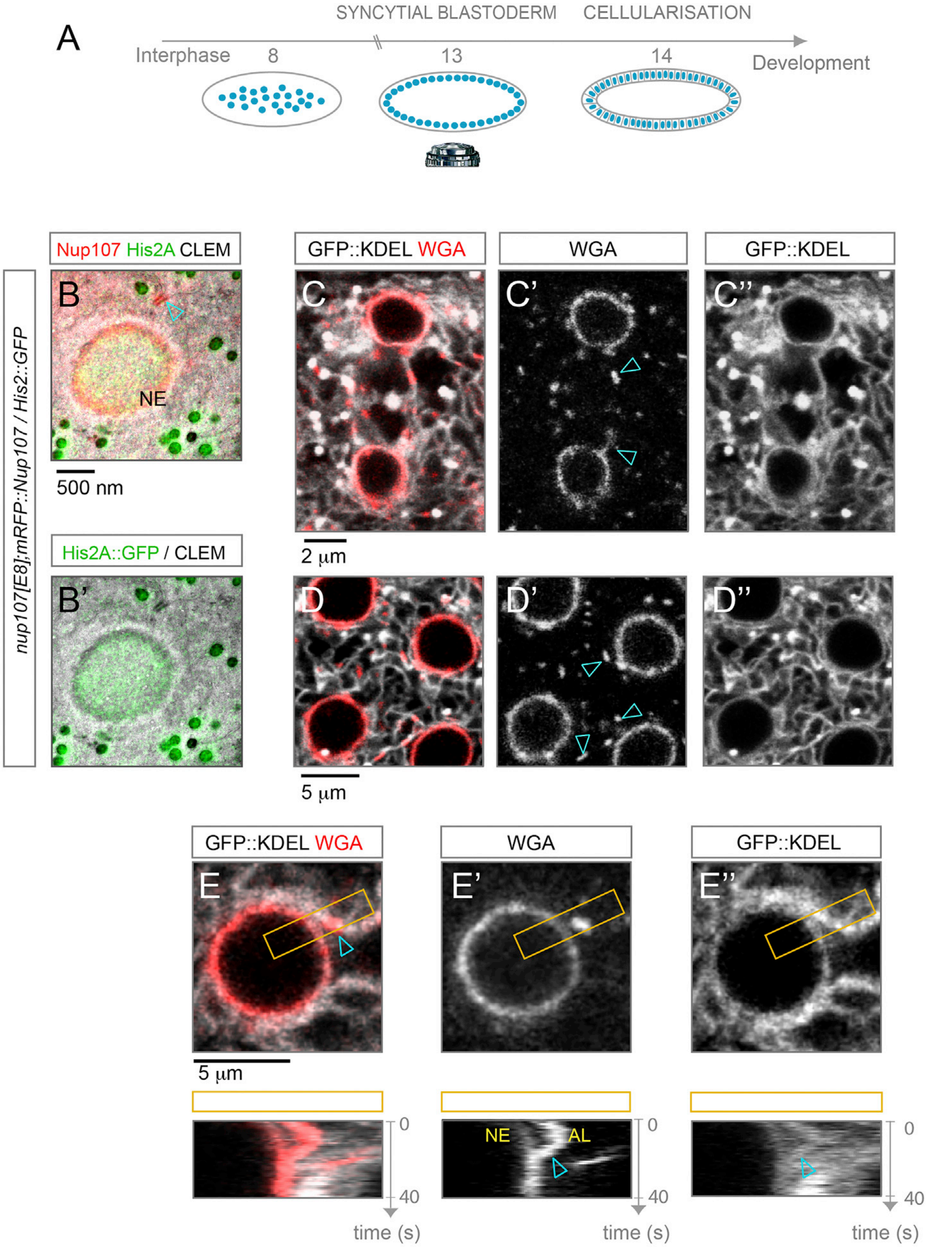

Figure S2. AL-NPCs Insert into the Nuclear Envelope, Related to Figure 1

(A) Schematic representation of early *Drosophila* embryogenesis. The somatic nuclei undergo 13 mitotic cycles in a common cytoplasm before formation of the first epithelial cell layer (cellularization) in the prolonged 14th interphase. Most of the presented experiments are performed in the syncytial blastoderm stage, where nuclei can be easily imaged by confocal microscopy due to their proximity to the microscope's objective adjacent to the surface of the embryo.

(B) Correlative light and electron microscopy (CLEM) of an embryo of the indicated genotype (see also Figures 1B–1B″). RFP::Nup107 fluorescence is concentrated along the NE and at AL-NPCs (arrowhead in B). Light microscopy images and electron micrographs of the same section are correlated by His2A::GFP in the nucleus and the auto-fluorescence of mitochondria in the GFP-channel (B′).

(C–E) AL-NPC's localize and insert along ER membranes. (C and D) Top view stills from a time lapse movie recording blastoderm embryos expressing the ER resident fusion protein GFP::KDEL (C″and D″), injected with WGA-Alexa555 labeling FG Nups (C′ and D′) in telophase (C–C″) or interphase (D–D″). AL-NPC reform on membranes at the spindle region in telophase (arrowheads in C′) and co-localize to ER membranes during interphase (arrowheads in D′). See also Movie S1.

(E) AL-NPCs insert to the NE along ER membranes. Top view still and kymograph covering the boxed region of interest (ROI) in (E–E″) from a movie of a KDEL::GFP expressing embryo injected with fluorescently labeled WGA. WGA labeled AL-NPCs insert along ER membrane into the NE (arrowheads). See also Movie S3.

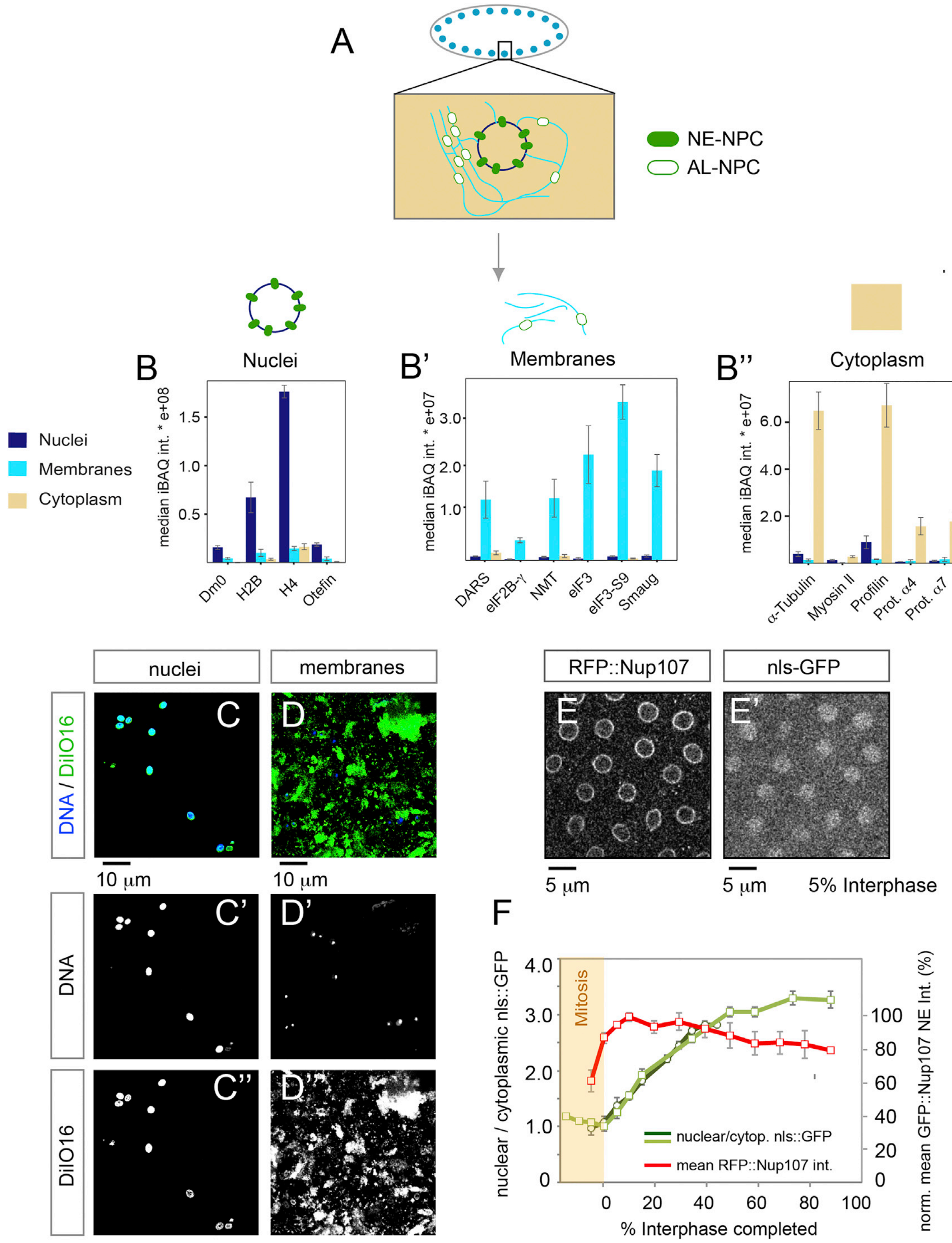

Figure S3. Sub-cellular Fractionation of Embryos, Related to Figure 2

(A) Biochemical fractionation of syncytial blastoderm *Drosophila* embryos.

(B–D) Quality controls of fractionation. (B–B″) Median intensity based absolute quantification (iBAQ) scores of selected control proteins representative for either nuclei, membranes or cytoplasm, measured in the three respective fractions (n = 3 biological replicates). (C and D) Immunofluorescence of nuclear and membrane fractions, stained for DNA and with the membrane dye DiIO16, respectively. Nuclear integrity is preserved in the nuclear fraction (C and C′), where DNA is enclosed by DiIO16 labeled NE. No further membranes are attached to nuclei (C″). No nuclei are in the membrane fraction (D and D′).

(E and F) Nuclear import of nls-GFP is delayed compared to NPC scaffold accumulation at the NE. Early interphase stills from a time lapse movie recording RFP::Nup107 (E) and nls-GFP (E′). (F) Mean fluorescence intensities of nuclear/cytoplasmic nls-GFP and of RFP::Nup107 at the NE were quantified in regions of interest (ROIs) on images as in (E and E′). Mean Intensities ± STDV (n = 23 nuclei in 2 embryos) are plotted as function of % interphase progression.

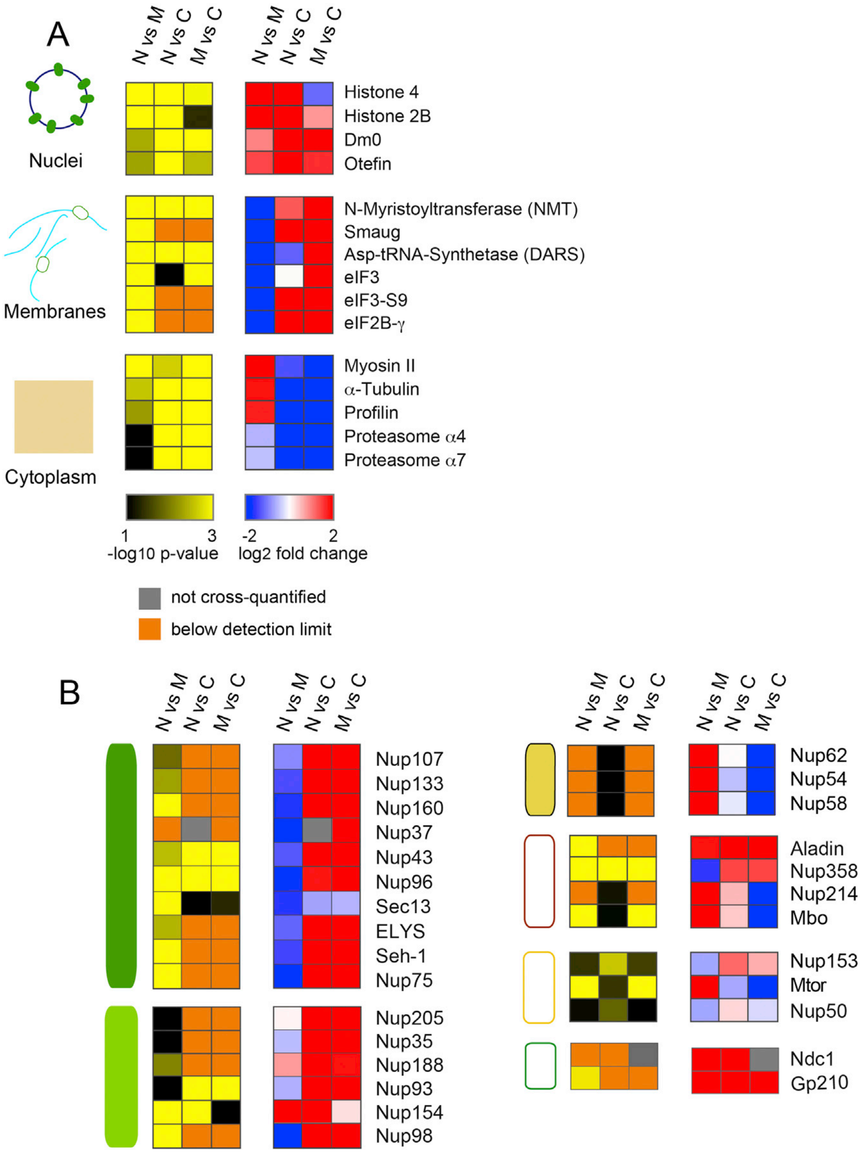

Figure S4. AL-NPCs Resemble Pore Scaffolds, Related to Figure 2

Alternative analysis and representation of the mass spectrometry data shown in Figures 2A and S3B–S3B″. Protein abundance fold-changes and respective p-values for control proteins (A) or Nups (B) are shown color-coded (n = 3 biological replicates). Orange color refers to cases in which the protein was consistently detected in three biological replicates of one fraction but was below the detection limit in all replicates of the other fraction (here, the fold change was set to 2). Nups are shown grouped into known subcomplexes; color-code according to Figure 2A.

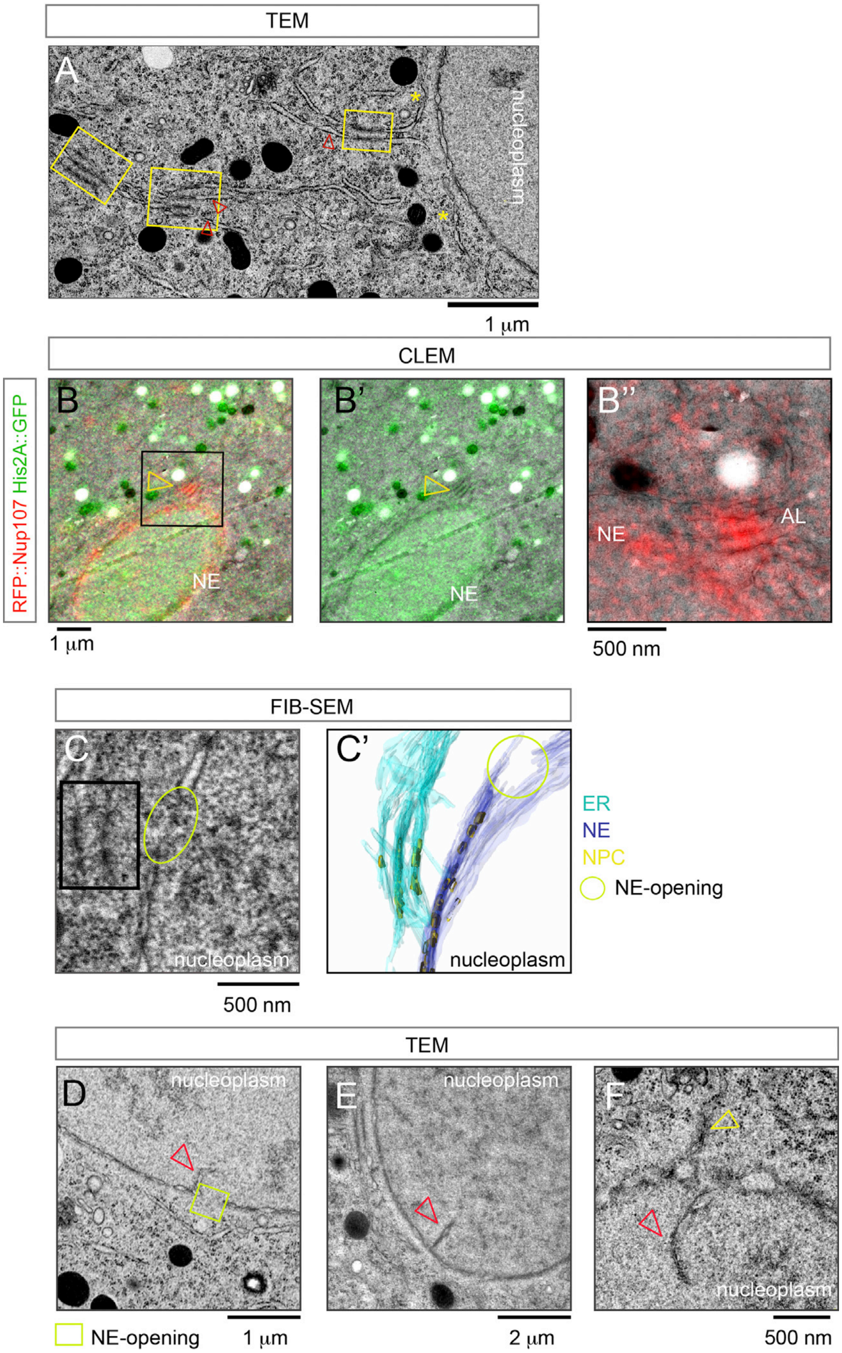

Figure S5. Topology of AL-Insertion, Related to Figure 3

(A) Transmission electron micrograph (TEM) of a *Drosophila* syncytial blastoderm embryo in interphase, showing the NE and adjacent AL (yellow boxes). AL appear as ribosome decorated (arrowheads) parallel ER stretches containing NPCs that morphologically resemble NPCs at the NE at the given resolution.

(B–B″) Overview (B and B′) and higher magnification (B″) micrographs obtained by correlative light and electron microscopy (CLEM) showing AL-insertion into the NE (see also Figure 3C). RFP::Nup107 fluorescence is concentrated along the NE and at AL-NPCs (arrowhead in B and B′, AL in B″). Light microscopy images and electron micrographs on the same section are correlated by His2A::GFP in the nucleus and the auto-fluorescence of mitochondria in the GFP-channel (B′).

(C–C′) Single slice (C) and isosurface-rendering (C′) of a volume obtained by Focused Ion Beam-Scanning Electron Microscopy (FIB-SEM), showing inter-connectivity of stacked, AL membrane sheets (black box in C) that are oriented parallel to the NE (C and C′). AL-membranes connect to the NE adjacent to NE-openings.

(D–F) TEM-sections depicting redundant NE membrane patches that are branched to the nuclear interior (red arrowheads in D–F). Note the redundant NPC containing membrane sheet close to an NE opening in (D). In (F) an inserting NPC containing ER stretch (yellow arrowhead) is close to redundant NE branching to the nucleoplasm (red arrowhead).

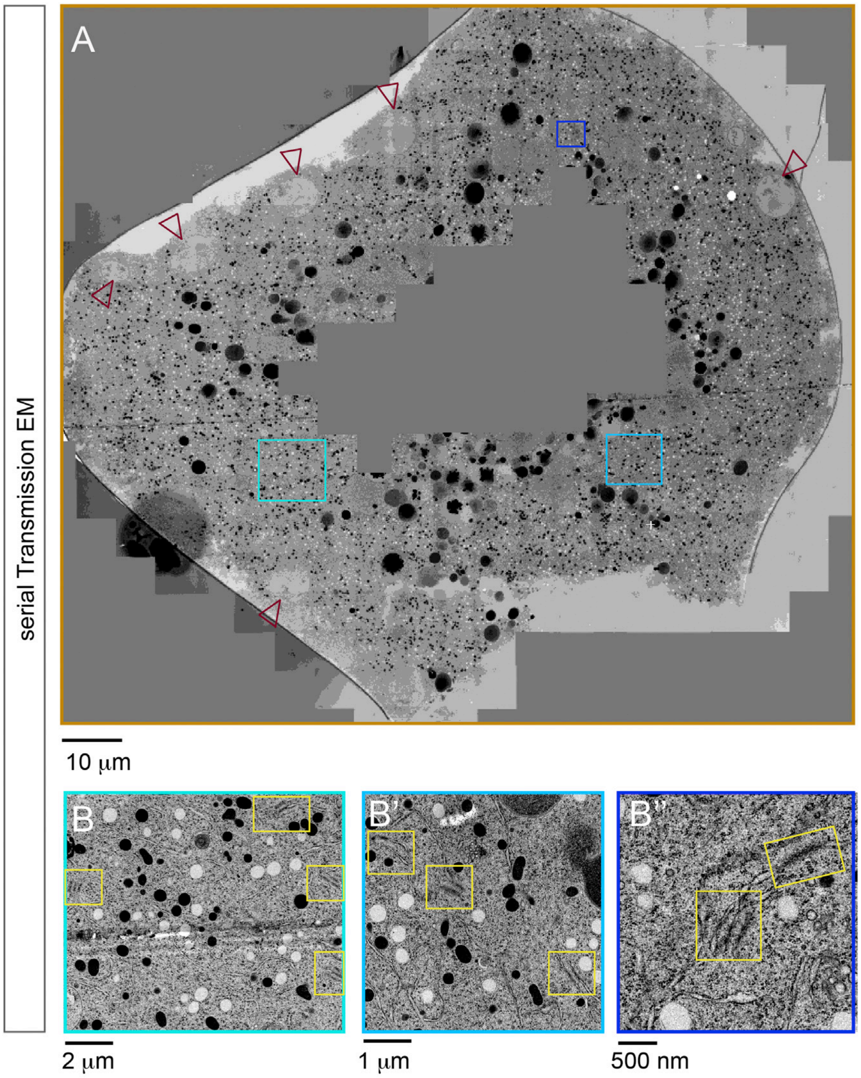

Figure S6. Annulate Lamellae Occur throughout the Embryo, Related to Figure 3

(A) Stitched Transmission electron micrographs (TEM) of an entire *Drosophila* syncytial blastoderm embryo in interphase, showing peripheral nuclei (arrowheads) and interior regions close to the yolk nuclei. AL occur close to cortical nuclei but also distant from dividing cortical nuclei (blue boxes).

(B–B″) Higher magnifications of the blue-boxed areas in (A), showing AL with NPCs on parallel membrane sheets (yellow boxes).

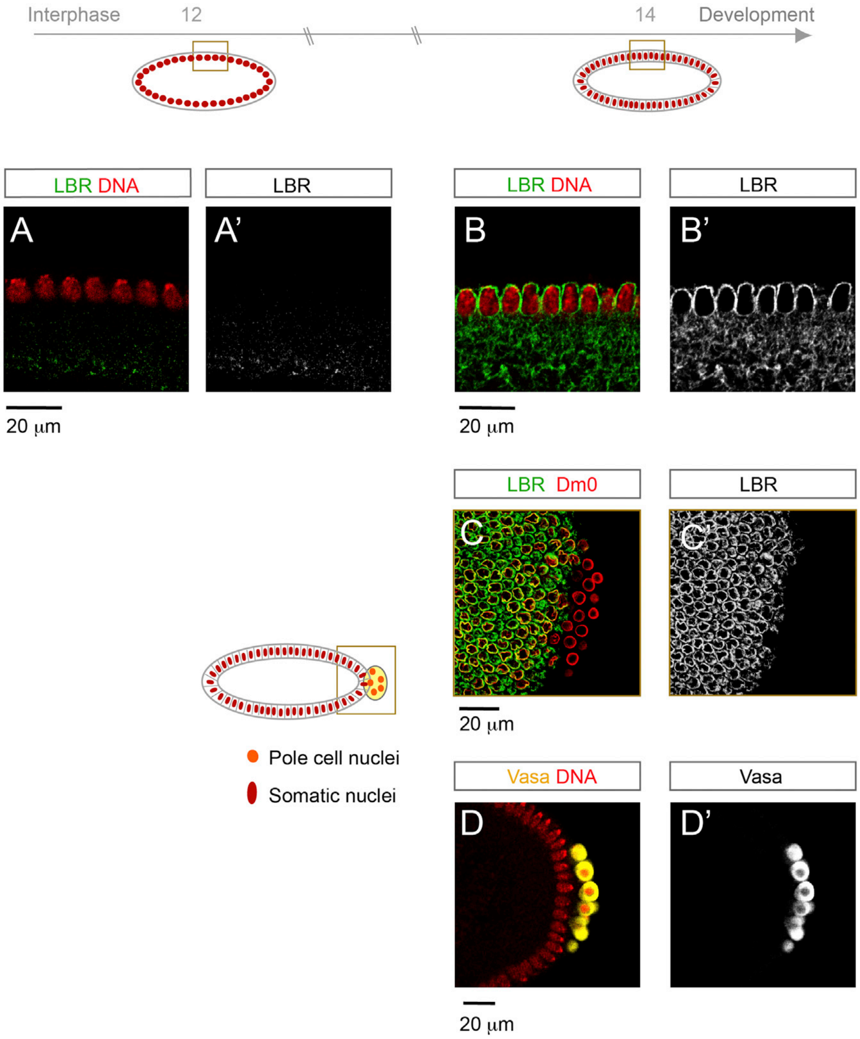

Figure S7. NE Targeting of LBR Is Developmentally Regulated, Related to Figure 6

Sagittal views (A, A′, B, B′, D, and D′) or optical cross section (C and C′) of fixed *Drosophila* embryos in the syncytial blastoderm stage (A and A′) or during cellularization in interphase 14 (B–D′). LBR does localize to the NE in the elongated prospective somatic nuclei during interphase 14 (B and B′) but not before (A and A′). (C and D) LBR targeting to the NE is cell type specific. Optical cross section of a fixed interphase 14 embryo stained with antibodies against the *Drosophila* Lamin Dm0 and LBR. Dm0 localizes to the periphery of somatic nuclei and the posteriorly located nuclei of pole cells, the germ cell progenitors. LBR is excluded from pole cell nuclei. Posterior pole cells stain positive for the Vasa protein, a specific pole cell marker (D and D′). Posterior is to the right in (C and D).

Cell, Volume *166*

Supplemental Information

Pre-assembled Nuclear Pores Insert

into the Nuclear Envelope

during Early Development

Bernhard Hampoelz, Marie-Therese Mackmull, Pedro Machado, Paolo Ronchi, Khanh Huy Bui, Nicole Schieber, Rachel Santarella-Mellwig, Aleksandar Necakov, Amparo Andrés-Pons, Jean Marc Philippe, Thomas Lecuit, Yannick Schwab, and Martin Beck

Supplemental Experimental Procedures

Fly strains and antibodies

The following fly lines were used in this study: *w*wg^{Sp-1}/CyO; P{mGFP-Nup107.K}9.1 (Katsani et al, 2008)* (BL-35514); *w*wg^{Sp-1}/CyO; P{mRFP-Nup107.K}7.1 (Katsani et al., 2008)* (BL-35517); *w*; Nup107^{E8}/CyO; P{mRFP-Nup107.K}7.1, P{His2Av$^{T:Avic\GFP-S65T}$}62A (Katsani et al., 2008),* (BL-35518); *w^{118}; P{w^{+},UASp-Lys::GFP-KDEL}; P{w^{+}, nanosGal4:VP16}*; *y^{1}w^{67c23} , P{w^{+mC}=Ubi-GFP.nls}ID2; P{Ubi-GFP.nls}ID3* (DGRC-106455). *yw* flies were used as wild type controls.

Embryo injections, Live Imaging and Immunostainings

For live-imaging analysis, staged syncytial blastoderm embryos of the respective genotype were treated as described (Cavey and Lecuit, 2008) and injected with Alexa488 or Alexa555 conjugated WGA (100 µg/ml, Life Technologies), α-amanitin (100 µg/ml, Sigma) or TRITC/FITC conjugated Dextrans of different molecular weight (Lenart and Ellenberg, 2006). Subsequently embryos were imaged on an inverted Zeiss LSM780 confocal microscope equipped with a 63x/1.4 NA oil immersion objective. Photo-bleaching was performed on GFP::Nup107 expressing embryos with a 488 nm Argon laser (100%, 5.2 mW photonic output) and 10 iterations. For laser-ablation of the NE, syncytial blastoderm embryos expressing GFP::Nup107 injected with 155kD-Dextran-TRITC were mounted and imaged on an inverted Zeiss LSM 780 NLO confocal microscope. Embryos were imaged using a 63x /1.3 NA oil objective and ROIs along the NE of individual nuclei were punctured by targeting a 950 nm Two Photon Laser at 100% laser power with one iteration. For photo-conversion

experiments, staged embryos expressing Eos-FP::Seh1 were processed in the dark and mounted on a Zeiss LSM510 inverted confocal microscope. Photo-conversion was done with a 405 nm laser with 5% laser power with 40 iterations. For Immunofluorescence experiments, staged embryos were dechorionated and fixed with 4 % Formaldehyde (FA) for 20 min, followed by Methanol de-vitellinization. Fixed embryos were proceeded for incubation with the following primary antibodies: mouse anti-Dm0 (1:500, DSHB), rat anti-Vasa (1:2000), rat anti-Mtor (1:500) (Mendjan et al., 2006), rabbit anti-Nup153 (1:500) (Mendjan et al., 2006), guinea pig anti-LBR (1:1000) (Wagner et al., 2004), mouse-anti Ab414 (1:500, Covance). Secondary antibodies were Alexa conjugates (1:500, Life Technologies), and DNA was stained with Hoechst dye (1:1000) Mounted stained embryos were imaged on Zeiss LSM780 or LSM510 confocal microscopes respectively. For Western Blot analysis antibodies were used in the following concentrations: mouse-anti Dm0 (1:100, DSHB), mouse-anti Ab414 (1:1000, Covance), mouse-anti α-Tubulin (1:5000, Sigma). To control the quality of biochemical fractionation, 10 ml of separated nuclear or membrane fraction respectively were incubated with Hoechst (1:5000) and DiIO16 (0.03 μg/ml, Thermo Fisher) for 10 min on poly-Lysin coated coverslips and imaged on a Zeiss LSM780 inverted confocal microscope.

Image analysis and quantifications

Imaging data was processed and analyzed using ImageJ or Fiji respectively. Kymographs were generated in Fiji. Generally, due to the increasing durations of interphases in the syncytial blastoderm stage, time courses were converted to and plotted as '% interphase completed'. Nuclear surfaces were determined as ellipsoidal approximations by measuring the maximal length of the axis and the nuclear height for each nucleus at every imaged time point for each respective movie. Mean fluorescence intensities of GFP::Nup107, RFP::Nup107 or

fluorescently labeled WGA was determined from manually designed ROIs around the NE or in the cytoplasm in consecutive z-planes of confocal stacks for multiple nuclei in an imaged embryo. Most apical and most basal sections were eliminated for quantifications and the residual planes were used to calculate the mean NE intensities for every nucleus at any given time point. To investigate the potential transfer of fluorescent material from AL-NPCs to NE-NPCs, integrated GFP::Nup107 intensities at the NE or in AL foci respectively were summed over consecutive, 1 μm distant z planes comprising ~10 nuclei. The field of view was kept constant over the time course of recording the respective embryo. Import capacity of nuclei was determined from averaging the ratios of nucleoplasmic to cytoplasmic mean GFP intensities measured in manually designed ROIs from multiple nuclei and adjacent cytoplasmic regions in embryos expressing Ubi::GFP.nls. To quantitatively assess the NE's permeability barrier, kymographs spanning the NE were generated from movies recorded in embryos expressing GFP::Nup107 that have been injected with TRITC labeled Dextran-155kD. Same-length linescans were computed using a custom Fiji plugin on the separated channels of the kymographs. The maximum value of the GFPNup107 along the linescan, representing the position of the NE, was used to align multiple ROIs relative to each other. NE-permeability was then assessed from the dextran-TRITC intensities within 20 pixels nucleoplasmic to the NE. To correct for differences in ROIs or embryos, these intensities were normalized by the mean of the Dextran fluorescence in the 10 pixels cytoplasmic to the NE for each respective measurement. For all quantifications pooled data was presented as means +/- STDV.

Constructs and generation of transgenic flies

To generate EosFP::Seh1 transgenes, the ORF of *Drosophila* Seh1 (Nup44) (CG8722) was cloned into pDONR221 (Life Technologies) using Gateway. A pUASp destination vector containing an EGFP tag upstream a Gateway cassette, obtained from Terence Murphy (Carnegie Institute), was modified by replacing EGFP with mEos-FP. The pUASp-EosFP-Seh1 cassette was used for standard *Drosophila* transgenesis and F_2 transgenes were identified by eye color. Maternal expression was driven by crossing EosFP::Seh1 males to *matatu>GAL4VP16* females. The cDNA from *Drosophila* LBR (CG17952) was obtained from the EST pOT2-LD38760 and cloned into pDONR221 (Life Technologies) and subsequently to pUASp using Gateway. Transgenes were generated using pUASp-LBR in standard transgenesis.

Sub-cellular fractionation and protein identification by mass spectrometry

Approximately 300 mg wet staged *Drosophila* embryos were dechorionated washed and lysed in Lysis buffer (200 mM Sucrose, 10 mM Tris pH 7.5, 25 mM NaCl, 5 mM $MgCl_2$). Nuclei were isolated by centrifugation at 5000 rpm for 13 min and stripped from attached membranes by centrifugation (45 min, 12000 rpm) through a 1 M Sucrose cushion. Microsomal membranes were isolated by spinning the supernatant from the nuclear precipitate at 40000 rpm for 45 min. The remaining supernatant was used as cytosolic fraction. Subsequently, fractions were lysed or supplemented by addition of Rapigest (Waters) and urea to a final concentration of 0.2% (v/v) and 4 M, respectively, and sonicated for 3x 30 sec. Samples were stored at -80°C before being further processed and analyzed by shot-gun mass spectrometry as previously described (Mackmull et al., 2015). The intensity-based absolute quantification (iBAQ) score of the proteinGroups.txt output of MaxQuant was used for further analysis. All comparative analyses were performed using R version 3.0.1. (The R Development Core Team

(2012) R: A Language and Environment for Statistical Computing, http://www.R-project.org/ Ed). Only proteins identified in at least 2 replicates were considered when comparing protein abundances between different fractions. To reduce technical variation data was quantile-normalized using the preprocessCore library (Gentleman et al., 2004). Protein differential expression was evaluated using the Limma package. Differences in protein abundances were statistically determined using the Student's t-test moderated by the empirical Bayes method. Significant regulated proteins were defined by a cut-off of log2 fold change ≤ -1 or ≥ 1 and p-value ≤ 0.01. The proteomics data have been deposited to the ProteomeXchange Consortium (http://proteomecentral.proteomexchange.org) (Vizcaino et al., 2014) via the PRIDE partner repository (Vizcaino et al., 2013) with the dataset identifier PXD004120.

Focused Ion Beam Scanning Electron Microscopy (FIB-SEM)

Dechorionated *Drosophila* embryos were staged by morphological criteria, cryo-immobilized by high-pressure freezing using a HPM010 (Abra Fluid) and freeze substituted at -90°C for 48 h using an EM-AFS2 (Leica Microsystems, Vienna, Austria) in a solution of 1% osmium tetroxide, 0.1% uranyl acetate and 5% water dissolved in anhydrous acetone. The temperature was increased 5°C per hour up to -30°C and the samples incubated for 3 h, followed by warming 5°C per hour up to 20°C and subsequent 5 h incubation. Samples were rinsed in acetone and stepwise infiltrated in durcupan resin (Sigma). Polymerization was done for 48 h at 60°C. Embedded embryos were trimmed and sectioned for TEM and inspected by EM to select ROIs in embryos of the correct cell cycle stage. Positions of ROIs relative to the section were noted and the sample was then trimmed for FIB-SEM. The TEM imaged surface becomes the imaging surface in the FIB-SEM, however the top surface of the sample where the platinum will be deposited needed to also be trimmed (Maco et al., 2014). Using the

position of the ROI relative to the section shape, the block was trimmed with a 90° diamond knife to position the ROI to within 3-10 µm of the block surface. The surface opposite to the exposed ROI was also trimmed to be parallel for mounting to the SEM stub (Agar Scientific). The sample was gold sputter coated (Quorum) and placed into the FIB-SEM for acquisition (Zeiss, Auriga 60). After ROI location a protective 1µm layer of platinum was deposited on the top face above the ROI using a gas deposit system operated under SmartSEM (Carl Zeiss Microscopy GmbH). Acquisition was performed with Atlas3D software (Fibics). Datasets were acquired with 5 nm pixel size and 5 nm steps in z and aligned using TrakEM (ImageJ). Segmentation and isosurface rendering were done in 3dmod 4.5.8.

Correlative Light and Electron Microscopy (CLEM)

For CLEM analysis, the embryos were high-pressure frozen (HPM010 AbraFluid), using 20% dextran as cryoprotectant. The embryos were pierced with a needle in a cryo-microtome chamber (Leica EM FC6) at -160˚C to facilitate freeze substitution (Eltsov et al., 2015). Embryos were then freeze-substituted (EM-AFS2 - Leica Microsystems) with 0.1% Uranyl Acetate (UA) in acetone at -90˚C for 48h. The temperature was then raised to -45˚C at 3.5˚C/h and samples were further incubated for 5h. After rinsing in acetone, the samples were infiltrated in Lowicryl HM20 resin, while raising the temperature to -25˚C and left to polymerize under UV light for 48 hours at -25°C and for further 9 hours while the temperature was gradually raised to 20˚C (5˚C/h). Thick sections (300 nm) were cut from the polymerized resin block and picked up on carbon coated mesh grids. Tomography was performed in 1degree increments at 4700x magnification on a FEI Tecnai F30 electron microscope.

The fluorescence microscopy (FM) imaging of the sections was carried out as previously described (Avinoam et al., 2015; Kukulski et al., 2011) using a widefield fluorescence microscope (Olympus IX81) equipped with an Olympus PlanApo 100X 1.40 NA oil immersion objective. Images were collected with mcherry-specific settings as well as in the green channel, to visualize the autofluorescence of heavily UA-stained mitochondria in the sample, which will serve as anchor points for the correlation.

TEM images were acquired with a FEI CM120 electron microscope. Correlation between light and electron micrographs was carried out with the plugin ec-CLEM (http://icy.bioimageanalysis.org/plugin/ec-CLEM) of the software platform Icy (de Chaumont et al., 2012). The position of mitochondria was manually assigned by clicking on the FM (green channel) and EM images. The coordinate of pairs in the two imaging modalities were used to calculate a linear transformation, which allowed to map the coordinates of the fluorescent spot of interest (red channel) and to overlay it on the electron micrograph.

Transmission Electron Microscopy (TEM)

Drosophila embryos were dechorionated, staged by morphology and cryo-immobilized by high-pressure freezing using a HPM010 (Abra Fluid). Freeze substitution of the embryos was done using a EM-AFS2 (Leica Microsystems, Vienna, Austria) in a solution of 0.3% glutaraldehyde, 0.3% uranyl acetate and 3% water dissolved in anhydrous acetone. The substitution was done at -90°C for 48 h. The temperature was increased at a 5°C per hour up to -45°C and the samples incubated for 16 h. The samples were rinsed in acetone and infiltrated in a graded series of acetone and HM20 resin, polymerization was done under UV light at -45°C for 48 h. The temperature was increased at a 5°C per hour up to 20°C and the polymerization was finished for 24h. For serial EM, the resin-embedded embryos were

trimmed and consecutive 100 nm distant sections were obtained with a section thickness of ~80 nm. Electron micrographs of these sections were recorded on a FEI Tecnai F30 equipped with Gatan US4000 CCD camera, operated at 300 kV or a FEI Biotwin equipped with an Olympus Keen View G2 camera operated at 120kV, respectively.

Supplementary References

Avinoam, O., Schorb, M., Beese, C.J., Briggs, J.A., and Kaksonen, M. (2015). ENDOCYTOSIS. Endocytic sites mature by continuous bending and remodeling of the clathrin coat. Science *348*, 1369-1372.

Cavey, M., and Lecuit, T. (2008). Imaging cellular and molecular dynamics in live embryos using fluorescent proteins. Methods Mol Biol *420*, 219-238.

de Chaumont, F., Dallongeville, S., Chenouard, N., Herve, N., Pop, S., Provoost, T., Meas-Yedid, V., Pankajakshan, P., Lecomte, T., Le Montagner, Y.*, et al.* (2012). Icy: an open bioimage informatics platform for extended reproducible research. Nat Methods *9*, 690-696.

Eltsov, M., Dube, N., Yu, Z., Pasakarnis, L., Haselmann-Weiss, U., Brunner, D., and Frangakis, A.S. (2015). Quantitative analysis of cytoskeletal reorganization during epithelial tissue sealing by large-volume electron tomography. Nat Cell Biol *17*, 605-614.

Gentleman, R.C., Carey, V.J., Bates, D.M., Bolstad, B., Dettling, M., Dudoit, S., Ellis, B., Gautier, L., Ge, Y., Gentry, J.*, et al.* (2004). Bioconductor: open software development for computational biology and bioinformatics. Genome Biol *5*, R80.

Katsani, K.R., Karess, R.E., Dostatni, N., and Doye, V. (2008). In vivo dynamics of Drosophila nuclear envelope components. Mol Biol Cell *19*, 3652-3666.

Kukulski, W., Schorb, M., Welsch, S., Picco, A., Kaksonen, M., and Briggs, J.A. (2011). Correlated fluorescence and 3D electron microscopy with high sensitivity and spatial precision. J Cell Biol *192*, 111-119.

Lenart, P., and Ellenberg, J. (2006). Monitoring the permeability of the nuclear envelope during the cell cycle. Methods (San Diego, Calif) *38*, 17-24.

Mackmull, M.T., Iskar, M., Parca, L., Singer, S., Bork, P., Ori, A., and Beck, M. (2015). Histone Deacetylase Inhibitors (HDACi) Cause the Selective Depletion of Bromodomain Containing Proteins (BCPs). Mol Cell Proteomics *14*, 1350-1360.

Maco, B., Cantoni, M., Holtmaat, A., Kreshuk, A., Hamprecht, F.A., and Knott, G.W. (2014). Semiautomated correlative 3D electron microscopy of in vivo-imaged axons and dendrites. Nat Protoc *9*, 1354-1366.

Mendjan, S., Taipale, M., Kind, J., Holz, H., Gebhardt, P., Schelder, M., Vermeulen, M., Buscaino, A., Duncan, K., Mueller, J.*, et al.* (2006). Nuclear pore components are involved in the transcriptional regulation of dosage compensation in Drosophila. Mol Cell *21*, 811-823.

Vizcaino, J.A., Cote, R.G., Csordas, A., Dianes, J.A., Fabregat, A., Foster, J.M., Griss, J., Alpi, E., Birim, M., Contell, J.*, et al.* (2013). The PRoteomics IDEntifications (PRIDE) database and associated tools: status in 2013. Nucleic Acids Res *41*, D1063-1069.

Vizcaino, J.A., Deutsch, E.W., Wang, R., Csordas, A., Reisinger, F., Rios, D., Dianes, J.A., Sun, Z., Farrah, T., Bandeira, N., *et al.* (2014). ProteomeXchange provides globally coordinated proteomics data submission and dissemination. Nat Biotechnol *32*, 223-226.
von Appen, A., Kosinski, J., Sparks, L., Ori, A., DiGuilio, A.L., Vollmer, B., Mackmull, M.T., Banterle, N., Parca, L., Kastritis, P., *et al.* (2015). In situ structural analysis of the human nuclear pore complex. Nature *526*, 140-143.
Wagner, N., Weber, D., Seitz, S., and Krohne, G. (2004). The lamin B receptor of Drosophila melanogaster. J Cell Sci *117*, 2015-2028.
